# Global predictors of language endangerment and the future of linguistic diversity

**DOI:** 10.1038/s41559-021-01604-y

**Published:** 2021-12-16

**Authors:** Lindell Bromham, Russell Dinnage, Hedvig Skirgård, Andrew Ritchie, Marcel Cardillo, Felicity Meakins, Simon Greenhill, Xia Hua

**Affiliations:** 1 Macroevolution and Macroecology, Research School of Biology, Australian National University, Canberra, Australian Capital Territory Australia; 2 ARC Centre of Excellence for the Dynamics of Language, Australian National University, Canberra, Australian Capital Territory Australia; 3 Department of Linguistic and Cultural Evolution, Max Planck Institute for the Science of Human History, Jena, Germany; 4 ARC Centre of Excellence for the Dynamics of Languages, School of Languages and Cultures, University of Queensland, Brisbane, Queensland Australia; 5 Mathematical Sciences Institute, Australian National University, Canberra, Australian Capital Territory Australia

**Keywords:** Language and linguistics, Macroecology

## Abstract

Language diversity is under threat. While each language is subject to specific social, demographic and political pressures, there may also be common threatening processes. We use an analysis of 6,511 spoken languages with 51 predictor variables spanning aspects of population, documentation, legal recognition, education policy, socioeconomic indicators and environmental features to show that, counter to common perception, contact with other languages per se is not a driver of language loss. However, greater road density, which may encourage population movement, is associated with increased endangerment. Higher average years of schooling is also associated with greater endangerment, evidence that formal education can contribute to loss of language diversity. Without intervention, language loss could triple within 40 years, with at least one language lost per month. To avoid the loss of over 1,500 languages by the end of the century, urgent investment is needed in language documentation, bilingual education programmes and other community-based programmes.

## Abstract (web summary)

Using a global analysis of 6,511 spoken languages with 51 predictor variables spanning aspects of population, documentation, legal recognition, education policy, socioeconomic indicators and environmental features, the authors identify predictors of current and future language endangerment and loss.

## Main

As with global biodiversity, the world’s language diversity is under threat. Of the approximately 7,000 documented languages, nearly half are considered endangered^1–8^. In comparison, around 40% of amphibian species, 25% of mammals and 14% of birds are currently threatened with extinction^9^. The processes of endangerment are ongoing^10^, with rates of loss estimated as equivalent to a language lost every one to three months^7,11,12^, and the most pessimistic predictions suggesting that 90% of the world’s languages will be lost within a century^13^. However, unlike biodiversity loss^14^, predictions of language loss have not been based on statistically rigorous analysis. Here we provide a global analysis to model patterns of current and future language endangerment, and compare the predictive power of variables representing some of the potential drivers of language loss. Our analysis has three key features. First, we examined a broader set of influences than previous studies, encompassing demographic factors, linguistic resources, socioeconomic setting, language ecology, connectivity, land use, environment, climate and biodiversity (Table 1). Second, we addressed major statistical challenges of large-scale comparative analyses, by simultaneously accounting for phylogenetic non-independence, spatial autocorrelation and covariation among variables. Third, our models incorporated demographic and environmental variables that can be projected into the future, allowing us to make predictions of future patterns of language endangerment in time and space.

**Table 1:** List of variables analysed in this study (see also Supplementary Fig. 3), with the names given to the variables in the raw data available in Supplementary Data 1

	Variable name	Level	Tr.	Sources
Response variable				
Endangerment level	EGIDS	Language		Glottolog V4.2.1
Independent variable				
0. Intercepts				
Region	Region	Language		naturalearthdata.com
Predictors				
1. Language				
L1 speaker population size	L1 pop	Language	L	WLMS e17, e16; worldgeodatasets.com
Area	Area	Language	L	WLMS e17, e16; worldgeodatasets.com
Island	Island	Language		See Supplementary Methods 2.1.3
Official status	Official status	Language		See Supplementary Methods 2.1.4
Level of language documentation	Documentation	Language		Glottolog V4.2.1
2. Diversity				
L1 Speakers as proportion of number of people in the neighbourhood	L1 pop prop	Language	L	WLMS and Glottolog
Number of languages in contact	Bordering language richness	Language	L	WLMS e17, e16; worldgeodatasets.com
Number of languages in contact per km perimeter	Bordering language richness per km	Language	L	WLMS e17, e16; worldgeodatasets.com
Evenness of languages in contact	Bordering languages evenness	Language	SR	WLMS e17, e16; worldgeodatasets.com
Number of languages	Language richness	Neighbourhood	L	WLMS e17, e16; worldgeodatasets.com
Language evenness	Language evenness	Neighbourhood	SR	WLMS e17, e16; worldgeodatasets.com
Number of endangered languages	Endangered languages	Neighbourhood	L	Glottolog V4.2.1
Proportion of languages that are endangered	Endangered prop languages	Neighbourhood	SR	Glottolog V4.2.1
3. Education				See Supplementary Tables 5 and 6
Recognized language of education	Language of education	Language		
Average years of schooling	Years of schooling	National	SR	Barro–Lee Educational Attainment database^77^; United Nations Development Programme 2018
Policy affirming minority language education	Minority education	National		L’aménagement linguistique dans le monde^78^
Education spending as % of GDP	Education spending	National	SR	World Bank 2019
4.Socioeconomic				See Supplementary Table 5
Gross Domestic Product per capita	GDPpc	National	L	World Bank 2019
GINI	GINI	National	S	Standardized World Income Inequality Database (SWIID)^79^
Life Expectancy at age 60	Life expectancy 60	National	L	World Bank 2019
5. Land use				
Population density	Pop density	Polygon	L	Gridded Population of the World (GPW) v4
Cropland	Cropland	Polygon	SR	Venter et al.^69^
Built environment	Built	Polygon	L	Venter et al.^69^
Pasture	Pasture	Polygon	SR	Venter et al.^69^
Human footprint	Human footprint	Polygon	SR	Venter et al.^69^
6. Environment				
Mean growing season	Growing season	Polygon		Global Agro-ecological Zones (GAEZ v3.0)^80^
Mean annual temperature	Temperature	Polygon	C	Worldclim v2
Temperature seasonality	Temperature seasonality	Polygon	L	Worldclim v2
Precipitation seasonality	Rainfall seasonality	Polygon	SR	Worldclim v2
7. Biodiversity loss				
Threatened species	Threatened species	Polygon	L	IUCN^9^
Proportion of species that are threatened	Threatened prop species	Polygon	L	IUCN^9^
8. Connectivity				
Road distance score	Roads	Neighbourhood	SR	Venter et al.^69^
Navigable waterways distance score	Waterways	Neighbourhood	SR	Venter et al.^69^
Landscape roughness	Roughness	Neighbourhood	S	SRTM30 elevation dataset
Altitudinal range	Altitude range	Neighbourhood	L	Worldclim v2
9. Shift				
Increase in urbanization	Urban change	National	SSR	World Bank 2019
Rate of change in population density	Pop density change	Polygon	SSR	GPW v4
Change in human footprint (score per year)	Footprint change	Polygon	SSR	Venter et al.^69^
Change in croplands (proportion of area per year)	Cropland change	Polygon	SSR	Venter et al.^69^
Change in pasture (proportion of area per year)	Pasture change	Polygon	SSR	Venter et al.^69^
Change in built environment (proportion of area per year)	Built change	Polygon	SSR	Venter et al.^69^
10. World language as official language				Supplementary Tables 2 and 3
Any	World language	National		
Arabic	Arabic	National		
Malay (including Indonesian)	Malay	National		
English	English	National		
French	French	National		
Hindustani (Hindi+Urdu)	Hindustani	National		
Mandarin	Mandarin	National		
Portuguese	Portuguese	National		
Russian	Russian	National		
Spanish	Spanish	National		

Level describes unit of estimation, whether based on information available for each language (‘language’), averaged over gridded data within the language polygon/s (‘polygon’), averaged over all gridded data for a 10,000 km^2^ circle centred on the language polygon (‘neighbourhood’), or information available at the national level, as a weighted average for the territories or nation states overlapped by each language polygon (‘national’). Endangerment level is based on EGIDS (Expanded Graded Intergenerational Disruption Scale) score from Glottolog V4.2.1^8^, analysed as an ordered 7-level scale (see Supplementary Table 1). Languages were assigned to regions as described in the Supplementary Methods (section 2.1.3). Language polygons are derived from World Language Mapping System (WLMS) as described in Supplementary Methods (section 2). Details of all variables are given in Supplementary Methods. The column ‘Tr.’ lists transformations applied to each variable following the procedure described in Supplementary Information (section 4.1; log (L), square (S), square root (SR), signed squared root (SSR), cube (C)).

While language change and shift are natural processes of human cultural evolution, the loss of global language diversity has been massively accelerated by colonization and globalization. Many factors contribute to language endangerment, some of which are specific to particular regions, language groups or languages. The historical context of each language, such as patterns of colonial expansion, and particular political climates, such as support for bilingual education, are expected to have substantial impacts on language endangerment patterns^10^. In addition to specific historical and local influences, there may also be widespread general factors that contribute to language endangerment, which can be used to identify languages that may come under increasing threat in the future. For a dataset containing 6,511 languages (over 90% of the world’s spoken languages), we analysed 51 predictor variables that target different aspects of language maintenance^15^, including language transmission (for example, whether a language is actively learned by children or used in education), language shift (for example, connectivity, urbanization, world languages) and language policy (for example, provision for minority language education, official language status). We also included variables that have been associated with language diversity, including features of climate and landscape. Clearly, any list of threatening processes will be incomplete, and the requirement for globally consistent data will fail to capture important influences on language vitality that operate at regional or local levels. Our aim is not to provide a comprehensive picture of language endangerment but a useful exploration of the influence of a selection of potential impacts. Broad-scale quantitative studies are therefore a complement to more focused qualitative studies on language endangerment and loss.

Understanding global threats to language diversity requires that we develop a macroecology of language endangerment and loss^16^. A macroecological approach has many advantages: it allows evaluation of a large range of factors that influence language vitality; formal testing of general patterns above the signal of individual language trajectories; statistical comparison of the explanatory power of different models, accounting for covariation of cultural, socioeconomic and environmental factors; and a way of avoiding the confounding effects of spatial distribution and relationships between languages^17^. Although threats to linguistic diversity, shaped by social, cultural, political and economic influences, often differ from processes threatening biodiversity^18^, the analytical challenges associated with studying global patterns of endangerment are common to biologists and linguists^17,19–21^. Here we use global analysis to illuminate some of the complex interactions of extrinsic factors threatening language diversity, and use this understanding to predict the fate of the world’s languages over the next century.

## Results and discussion

### Current patterns of endangerment

**Fig. 1:**
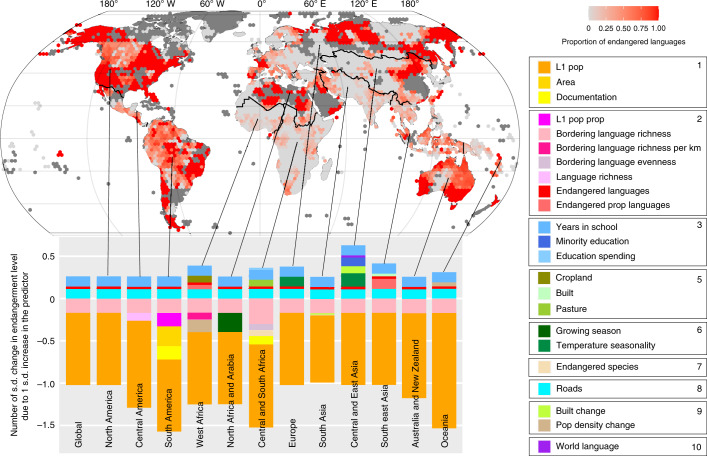
Current patterns of language endangerment expressed as the proportion of languages overlapping each hex grid that are currently rated threatened or above (EGIDS 6b–10; see Supplementary Information Table 1). Each hexagon represents approximately 415,000 km^2^. The coloured bars show the predictors of level of endangerment identified in the best model for a global language database of 6,511 languages, and for each of 12 regions any additional influences on patterns of language endangerment (see Supplementary Data 3). Dark grey areas on the map do not have data for all the independent variables in the best model for language endangerment level. Language distribution data are from WLMS 16 (worldgeodatasets.com).

**Extended Data Fig. 1:**
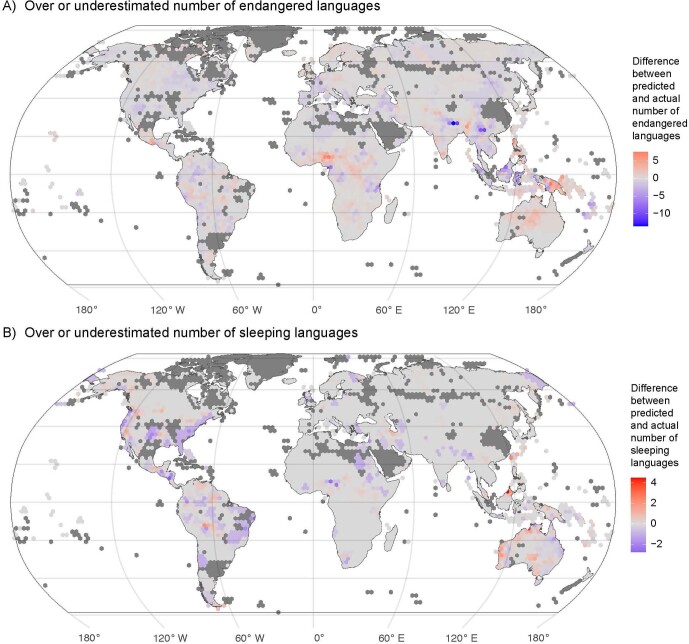
Residual in the best model for language endangerment level. Residuals of the model predicting number of endangered languages (**a**) and Sleeping languages (**b**), calculated, for each hex grid, as the predicted number of languages with distribution in the hex grid and with (A) predicted endangerment level above ‘Stable’ (corresponding to EGIDS 6b-10) and (B) predicted to be no longer spoken (ie EGIDS 9-10) minus the number of languages with distribution in the hex grid and with reported EGIDS from 6b-10 (A) and from 9-10 (B). The predicted number of languages in each category is calculated by using the best model to estimate the probability distribution of endangerment level for each language with distribution in the hex grid, sampling from the probability distribution the endangerment level of each language, repeating the sampling 1000 times, and averaging the number of languages with sampled endangerment level of endangered or Sleeping over the 1000 times. A negative value (blue) indicates that the model estimates fewer endangered or Sleeping languages than the reported EGIDS score from Ethnologue (e17/e16). A positive value (red) indicates the model estimating a greater number of endangered or Sleeping languages than observed. In some cases, this could indicate higher ‘latent risk’, for languages that have many of the predictors of high endangerment but are currently rated as stable or at a lower level of endangerment. Dark grey areas do not have data for all the independent variables in the best model for language endangerment level. Language distribution data from WLMS 16 worldgeodatasets.com.

**Extended Data Fig. 2:**
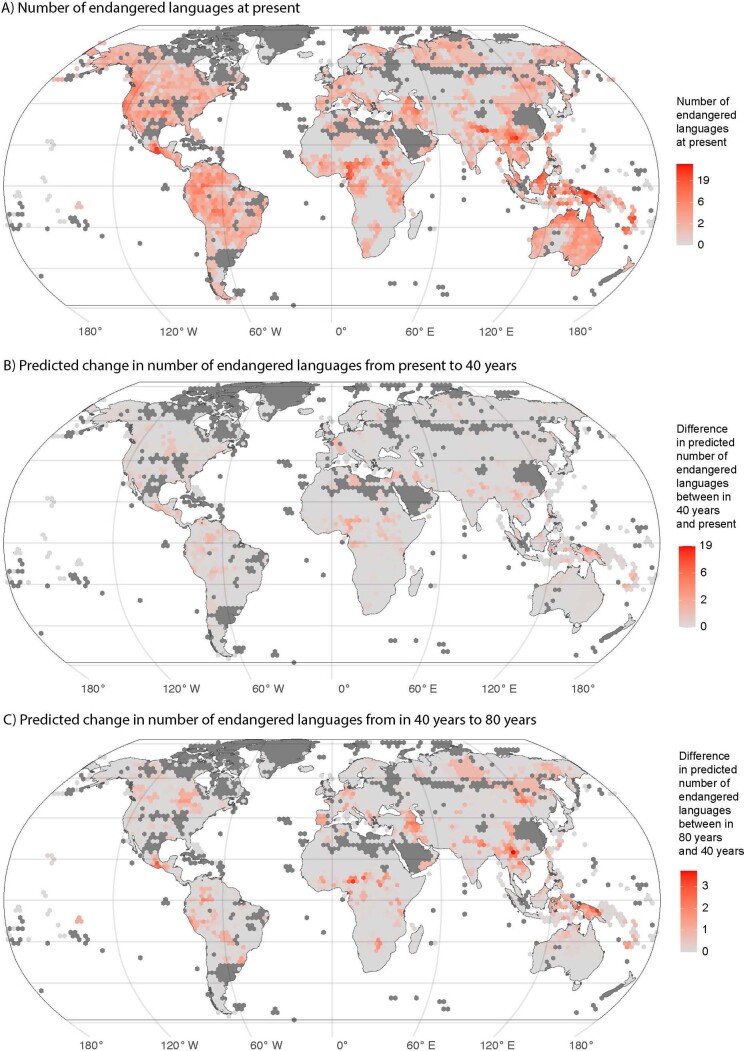
Current and future predicted distribution of endangered languages. The current patterns of language endangerment are plotted as absolute number of languages with a reported EGIDS score of 6b-10 with distribution in each hex grid. **a**) the number of languages with observed EGIDS from 6b to 10 at present. **b**) the predicted number of languages with EGIDS from 6b to 10 in 40 years minus the predicted number of languages with EGIDS from 6b to 10 at present. **c**) the predicted number of languages with EGIDS from 6b to 10 in 80 years minus the predicted number of languages with EGIDS from 6b to 10 in 40 years. The predicted number of languages is calculated in the same way as Supplementary Fig. 7. Dark grey areas have no data for independent variables in the best model for language endangerment level. Language distribution data from WLMS 16 worldgeodatasets.com.

We use an endangerment scale based on EGIDS, which incorporates a range of factors including domains of use and intergenerational transmission^22,23^. We describe languages that are losing first-language (L1) speakers as ‘Threatened’, those with only adult speakers and no child learners as ‘Endangered’ or those with only elderly speakers as ‘critically endangered’, and languages with no L1 speakers as ‘Sleeping’ (the term preferred by many speakers of endangered languages^1,24,25^: Supplementary Table 1). Of the 6,511 languages in our database, 37% are considered threatened or above (which we will refer to generally as ‘endangered languages’); 13% of these are no longer spoken (sleeping). The areas of greatest absolute number of endangered languages are in New Guinea, Central America, Himalayas and Yunnan, and regions between Central and West Africa (Extended Data Figs. 1 and 2), but this pattern may largely reflect diversity^17^: where there are more languages, speaker populations and geographic ranges tend to be smaller, potentially resulting in more endangered languages. Areas with the highest proportion of their languages endangered include Australia, North China, Siberia, North Africa and Arabia, North America, and parts of South America (Fig. 1). Areas with the greatest language loss to date are in Australia, South America and USA (Extended Data Fig. 2).

### Predictors of language endangerment

Our analysis seeks the best set of variables, from 51 candidate variables, to explain variation in endangerment level (the dependent variable), over and above covariation due to relationships between languages, spatial autocorrelation and contact between language distributions, and allowing for interactions between predictor variables and region. We reduced the number of variables by grouping variables according to their pairwise correlations, identified independent variables with significant predictive power on a proportion of the data (training dataset), then evaluated the fit of the model on the remaining data (test dataset). We then estimated model parameters on the full dataset (see Methods for details).

Our best-fit model explains 34% of the variation in language endangerment (comparable to similar analyses on species endangerment^26–28^). These variables cannot provide a full picture of the processes threatening language diversity, as there will be many other important factors that cannot be included due to lack of appropriate and consistent data with global coverage, or because of the idiosyncratic nature of processes of language endangerment and the influence of historical factors that cannot be captured in a broad-scale model. For example, patterns of human migration and past episodes of population expansion and contraction will not be captured fully in contemporary language distribution data. Furthermore, language endangerment and loss is an ongoing process, and there may be historical factors that caused dramatic reduction in L1 speakers that will not be captured in current values of socioeconomic variables, such as massacres of Indigenous populations or ethnic groups, punishing people for speaking their language and separating children from parents. Patterns of language endangerment may at least partially reflect past influences, such that current predictors might not fully capture important processes that resulted in the current endangerment status (a phenomenon known in conservation biology as extinction filter effect^29^). Because of these unavoidable limitations, no study of this kind can aim to comprehensively describe factors affecting vitality of all of the world’s languages. But by identifying contemporary factors that are significant predictors of current patterns of endangerment at a global scale, we contribute to the understanding of the complex interaction of factors contributing to language endangerment.

Five predictors of language endangerment are consistently identified at global and regional scales: L1 speakers, bordering language richness, road density, years of schooling and the number of endangered languages in the immediate neighbourhood. Each of these predictors highlights a different process in language endangerment; taken together, they paint a picture of the way interactions between languages shape language vitality.

Number of first-language (L1) speakers is the greatest predictor of endangerment. It is important to emphasize that not all small languages are endangered, and that language loss does not necessarily result from a reduction in number of people in a particular culture or population, but often occurs when people shift from using their heritage language to a different language^1,30^. Therefore the multilingual setting in which each language is embedded (referred to as the language ecology) plays a key role in endangerment, by influencing whether speakers shift to another language or adopt additional languages in their multilingual repertoires^31^. Our results suggest that direct contact with neighbouring languages, as reflected in the number languages with overlapping or touching distributions, is not in itself a threatening process. In fact, languages whose distributions are directly in contact with a greater number of other autochthonous languages have lower average endangerment levels (Fig. 1). This may reflect a common observation that communities in regular contact with speakers of other Indigenous languages may be multilingual without necessarily giving up their L1 language^31^. If ongoing language contact was a threat to language vitality, then we might expect that more isolated languages, such as those on islands, would be less endangered, but this is not the case (Supplementary Fig. 7). Similarly, we find no evidence that barriers to human movement that might be expected to reduce contact between nearby speaker populations, such as steep or rough terrain, are associated with reduced endangerment. We conclude that being in regular contact with speakers of another language does not in itself usually endanger Indigenous language vitality. Instead there are other more complex social, economic and political dynamics influencing language endangerment that may co-occur with language contact but are not synonymous with it.

A language is more likely to be endangered if a higher proportion of languages in the region are also endangered, suggesting that, in addition to language-specific threats, there are also widespread factors that influence language vitality across a region. One such factor is the density of roads in the neighbourhood surrounding each language (Fig. 1). One interpretation of the association between road density and language endangerment is that roads increase human movement and thus bring people into contact with speakers of other languages, and this may result in language shift. However, our results suggest that the association between language endangerment and roads is unlikely to simply reflect language contact. If language contact always generated language shift and loss, then we would expect languages with a high degree of contact with other languages to be more endangered. In fact, we find the opposite: languages whose distribution overlaps or meets many other languages are less endangered (Fig. 1 and Supplementary Data 3). Furthermore, if contact between speakers of different languages was a driver of language loss, then we would expect landscapes that inhibit movement to reduce language contact and show lower levels of endangerment, but none of the other connectivity variables, such as altitudinal range, landscape roughness or density of waterways, show consistent association with language endangerment. The association with roads is neither simply a result of socioeconomic shift, as other indicators of development (for example, GDP, life expectancy) are not associated with language endangerment, nor is it a reflection of increasing urbanization, land use change or increase in built environment (Supplementary Fig. 7). Instead, road density may reflect connectivity between previously remote communities and larger towns, with increase in the influence of commerce and centralized government. Lack of roads has been cited as a protective factor in maintaining Indigenous language vitality, as it may limit the spread of ‘lingua francas’, such as Tok Pisin in Papua New Guinea^32^. The association between road density and language endangerment may reflect movement of people in two directions, as people move from their traditional homelands into larger population centres, and outsiders move into previously isolated communities, both of which have been implicated as threats to Indigenous language vitality^33^. For example, access to new employment opportunities (such as a shift from rural work to factory or construction work) may result in shift away from heritage languages to dominant languages of commerce^34–36^. Roads can aid the spread of ‘lingua francas’ or languages of central governance^37^.

There is consistent global support for higher average levels of schooling being associated with greater language endangerment (Fig. 1). The association between schooling and language endangerment cannot be interpreted as a side effect of growing socioeconomic development, because years of schooling is a much stronger predictor of endangerment patterns than other socioeconomic indicators. Instead, it is consistent with a growing number of studies showing a negative impact of formal schooling on minority language vitality, particularly where bilingual education is not supported or, in some cases, is actively discouraged^38–40^. Yet having a minority education policy is not globally associated with reduced threats to language diversity, possibly due to variation in the extent and manner of provision of bilingual education for speakers of minority languages. For example, the Bilingual Education Act of the United States (1968) was primarily concerned with improving access to mainstream education for students from non-English speaking backgrounds by using heritage language as a bridge to English acquisition, rather than being designed to allow students to maintain their first language^41^.

The spatial scale of the variables reflecting education policy and outcomes cannot capture variation within countries. Reliable statistics on average years of schooling are, for most parts of the world, only available as national averages, even though years of schooling may vary within a country, particularly between socioeconomic groups, or when comparing rural and urban populations. However, we note that the same effects have been reported in local-scale studies: for example, in a remote northern Australian Indigenous community, increased number of years schooling is associated with reduced use of Indigenous language elements across all generations, from elders to children^42^. Collection of regional data on variation in number of years of schooling would allow the generality of this relationship to be tested at a range of spatial scales.

Similarly, our data on education policy is necessarily coarse grained, which may mask some patterns at local scales: national legal provision may not reflect use of minority languages in schools at a local or regional level. For example, in China, the Regional Ethnic Autonomy Law (1984) promotes learning both regional languages and Mandarin Chinese, but the policy is not translated into educational practice evenly across all regions due to lack of resources in some languages, or local emphasis in some places on students from minorities learning the centralized language of governance and commerce^39^. The same bilingual education policy may invigorate minority languages in some areas, but result in greater emphasis on education in the dominant national language in other places^43^. More fine-grained analysis at regional level is needed to examine the influence of minority languages in classrooms on language diversity and vitality.

Our results not only identify global threats to language vitality, but also reveal differences in threatening processes in different regions. For example, in Africa, language endangerment is associated with greater areas of pasture or croplands, potentially reflecting language shift associated with subsistence change (for example, as hunter–gatherer societies adopt the languages of neighbouring pastoral or agricultural groups^44^). Climate has the strongest association with language endangerment in Europe, with endangerment levels increasing with temperature seasonality, reflecting patterns of language erosion in Arctic regions. These regional patterns are ideal foci for future studies of language endangerment: while the current study is constrained to predictors that are globally relevant and consistently measured for all regions of the world, a targeted study could focus on variables considered important at regional scales, such as land use and subsistence in Africa, population density change in Oceania, or climate in Europe and Central and Eastern Asia (Supplementary Fig. 7).

### Predicting future language loss

If a language is no longer being learned by children, we can use demographic information to predict when, in the absence of interventions to increase language transmission, there will be no more living L1 speakers. We can combine the current L1 speaker population size with endangerment score (which tells us the relative generational distribution of L1 speakers and whether the number of L1 speakers is declining; Supplementary Table 1), and use demographic information on age structure of the population (Supplementary Table 8) to predict how many L1 speakers will be alive in the future (see Supplementary Methods 5 for details). Our analysis is conservative in that it only considers change in L1 speakers in languages identified as having reduced transmission to younger generations (see Supplementary Table 1): we did not model change in speaker number for languages currently considered to be stably transmitted, even though they may become endangered in the future.

**Fig. 2:**
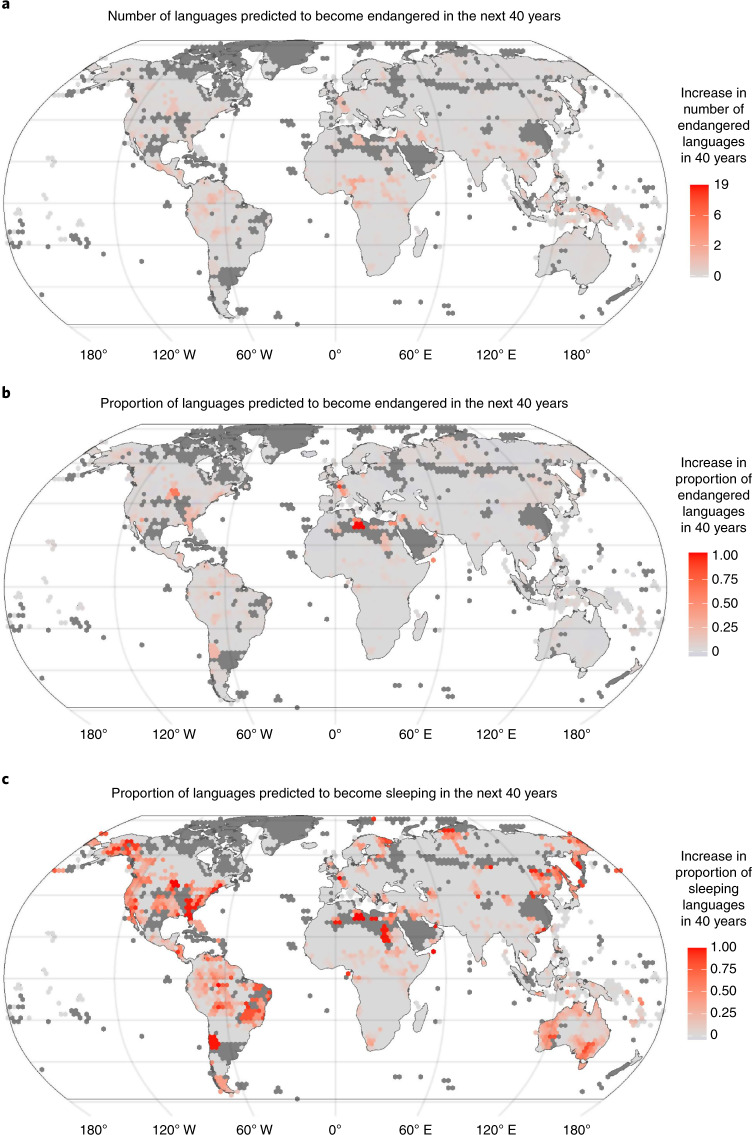
Model predictions for areas where languages are likely to become endangered (EGIDS ≥ 6b) in the next 40 years, given the best model. **a**,**b**, The red shading represents the differences between the predicted values at present and the predicted values in 40 years, for the absolute number (**a**) and proportion of languages (**b**) per hex grid, based on generational shift and demographic transition in L1 speakers. **c**, Proportion of languages predicted to become Sleeping (EGIDS ≥ 9) in the next 40 years. See Supplementary Table 1 for information on endangerment scales. Language distribution data from WLMS 16 (worldgeodatasets.com).

**Fig. 3:**
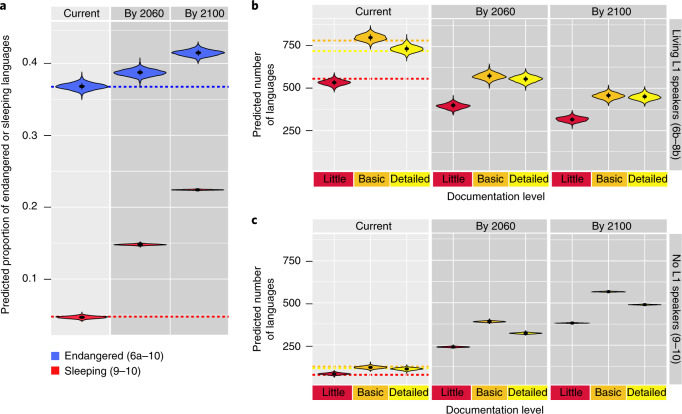
Estimated future loss of linguistic diversity. **a**, Current and predicted proportion of languages that are endangered (EGIDS 6b–8b) or Sleeping (no living L1 speakers, EGIDS 9–10). **b**,**c**, Current and predicted number of endangered (6b–8b) (**b**) and Sleeping (9–10) (**c**) languages according to the current level of language documentation. Each violin gives the probability distribution of the number or proportion of languages that are predicted to be endangered or Sleeping, with the dot showing the mean and the whisker showing the standard deviation. Each dashed line shows the number or proportion of languages that are currently endangered or Sleeping. This figure projects current levels of documentation for each language, hence does not reflect future documentation efforts of threatened languages.

Our model predicts that language loss will at least triple in the next 40 years (Fig. 2). Without intervention to increase language transmission to younger generations, we predict that by the end of the century there will be a nearly five-fold increase in Sleeping languages, with at least 1,500 languages ceasing to be spoken (Fig. 3). Some parts of the world stand out as ‘hotspots’ of future language loss, with the greatest absolute loss of languages predicted to occur in the west coast of North America, Central America, the Amazon rainforest, West Africa, north coast of New Guinea and northern Australia (Extended Data Fig. 4). After 80 years, the model predicts additional areas of language loss in Borneo, southwest China and areas around the Caspian Sea. The greatest proportional loss of languages is predicted to occur in the Arctic, interior plains of Northern America, temperate areas of southern Chile and the Sahara (Extended Data Fig. 5).

**Extended Data Fig. 3:**
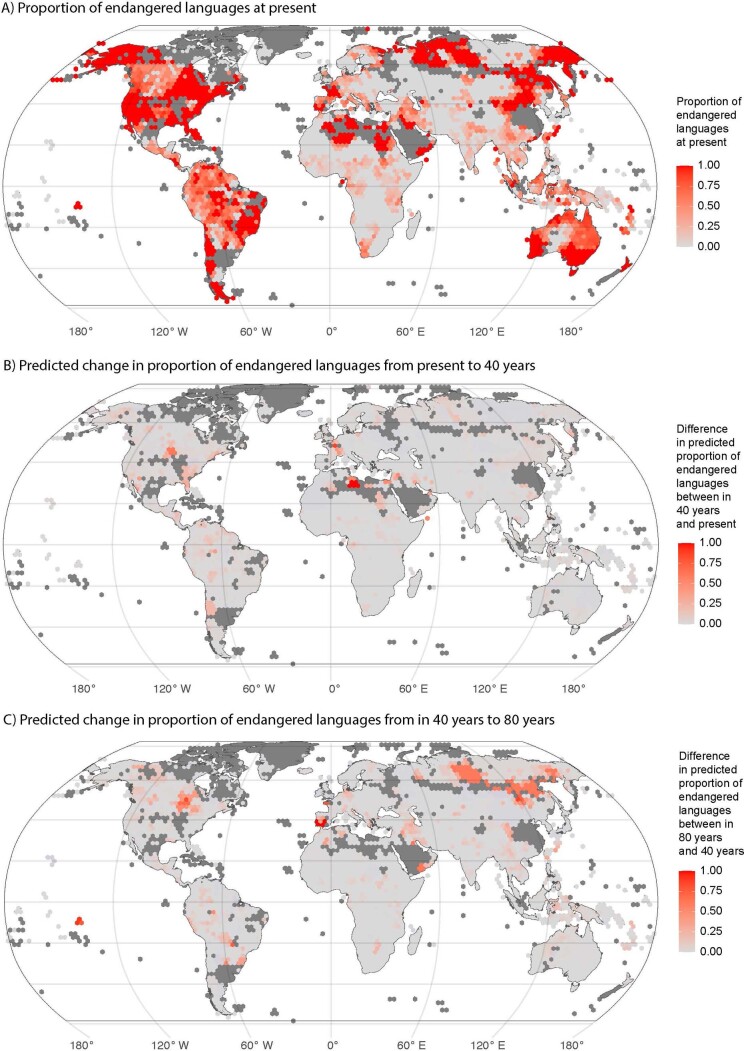
Current and future predicted proportion of endangered languages. **a**) the proportion of languages with observed EGIDS from 6b to 10 at present. **b**) the predicted proportion of languages with EGIDS from 6b to 10 in 40 years minus the predicted proportion of languages with EGIDS from 6b to 10 at present. **c**) the predicted proportion of languages with EGIDS from 6b to 10 in 80 years minus the predicted proportion of languages with EGIDS from 6b to 10 in 40 years. The predicted proportion of languages is calculated as the predicted number of languages divided by the total number of languages with distribution in each hex grid, where the predicted number of languages is calculated in the same way as Fig. 7. Dark grey areas have no data for independent variables in the best model for language endangerment level. Language distribution data from WLMS 16 worldgeodatasets.com.

**Extended Data Fig. 4:**
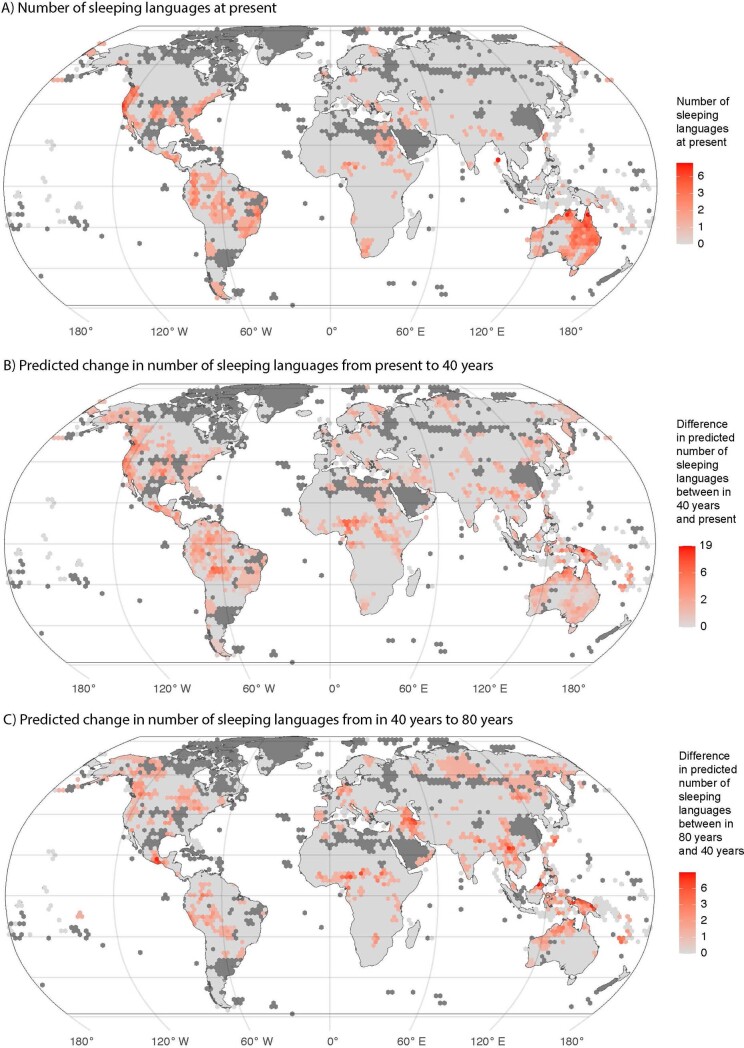
Current and future predicted number of languages no longer spoken. **a**) the number of languages with observed EGIDS from 9 to 10 at present. **b**) the predicted number of languages with EGIDS from 9 to 10 in 40 years minus the predicted number of languages with EGIDS from 9 to 10 at present. **c**) the predicted number of languages with EGIDS from 9 to 10 in 80 years minus the predicted number of languages with EGIDS from 9 to 10 in 40 years. The predicted number of languages is calculated in the same way as Fig. 7. Dark grey areas have no data for independent variables in the best model for language endangerment level. Language distribution data from WLMS 16 worldgeodatasets.com.

**Extended Data Fig. 5:**
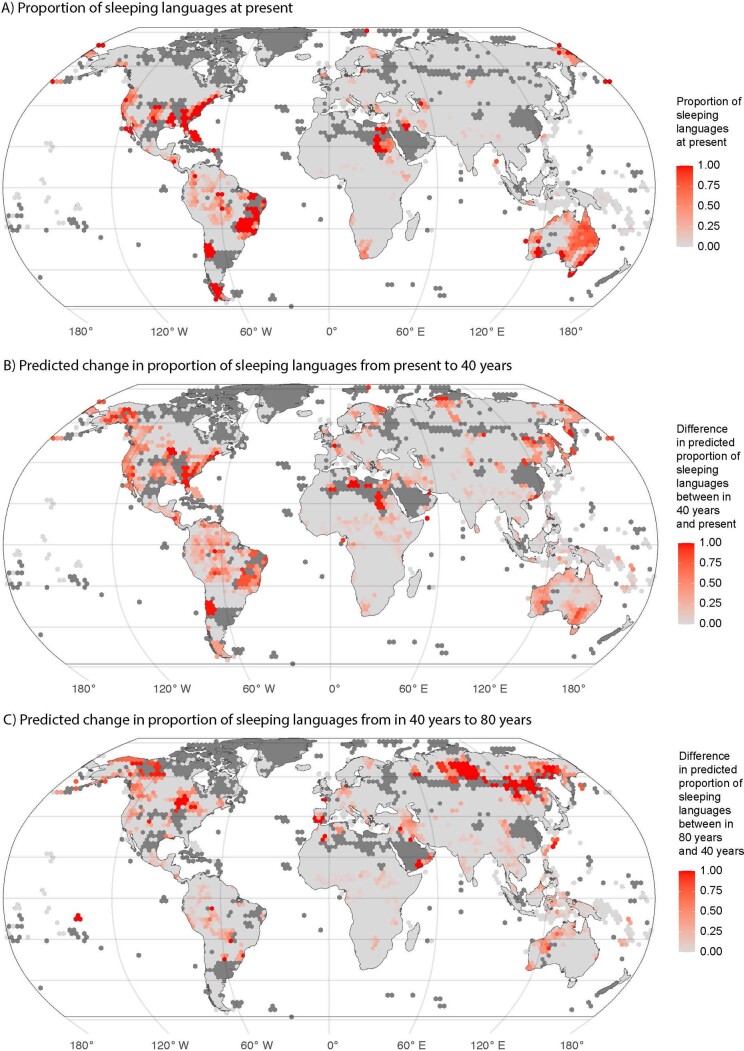
Current and future predicted proportion of languages no longer spoken. The proportion of Sleeping languages with distribution in each hex grid. **a**) the proportion of languages with observed EGIDS from 9 to 10 at present. **b**) the predicted proportion of languages with EGIDS from 9 to 10 in 40 years minus the predicted proportion of languages with EGIDS from 9 to 10 at present. **c**) the predicted proportion of languages with EGIDS from 9 to 10 in 80 years minus the predicted proportion of languages with EGIDS from 9 to 10 in 40 years. The predicted proportion of languages is calculated as the predicted number of languages divided by the total number of languages with distribution in each hex grid, where the predicted number of languages is calculated in the same way as Fig. 7. Dark grey areas have no data for independent variables in the best model for language endangerment level. Language distribution data from WLMS 16 worldgeodatasets.com.

In addition to demographic shift, our model also identifies predictors of language endangerment that are likely to change over time. For some of the variables associated with language endangerment, such as average years of schooling, we lack an adequate predictive model that is global in extent but would allow for regional variation. However, there are some variables identified as significant predictors of language endangerment at regional levels, such as land use and climate, for which we can predict future values on the basis of current trends (see Supplementary Information 5.2). For example, we can use climate change models to predict future values of climate variables at all points of the globe, and we can use information on rates of change in land use in each grid cell to project possible future values for land use variables in that grid cell. Clearly, such predictions should be regarded as possible values only, and all such future projections are subject to caveats: for example, we chose a mid-range climate model so the future values could be higher or lower depending on the effectiveness of global climate change mitigation strategies, and the land-use projections are based on the average rate of change in the last few decades, although local factors may cause those rates of change to either increase or decrease in the future. But it is a useful exercise to add climate and land use to the predictive model to illustrate the potential for forward prediction of variables impacting endangerment status. The results of the predictions based on generational shift and demographic transition are shown in Figs. 2 and 3. Predictions that are additionally adjusted for change in climate and land-use variables show qualitatively the same results (Extended Data Figs. 2–5).

### Safeguarding language diversity

The crisis of language endangerment has prompted worldwide efforts to recognize, document and support language diversity^45^, reflected in the UNESCO International Decade of Indigenous Languages, beginning in 2022. Every language represents a unique expression of human culture, and each is subject to idiosyncratic influences of their specific history and local sociopolitical environment. By identifying general factors that impact language vitality, or areas at greatest risk of language loss, we may be better placed to direct resources for maintenance of language diversity.

In biology, ‘extinction debt’ describes the inevitable loss of species that are currently persisting with inviable populations or insufficient habitat^46,47^. For languages, ‘extinction debt’ arises from reduced intergenerational transmission. Languages currently spoken by adults but not learned as a first language by children will, without active intervention and revitalization, have no more L1 speakers once the current L1 speakers die. Using information on intergenerational transmission for each language combined with demographic information, our model predicts that the greatest increase in endangered languages will coincide with areas of greatest language diversity, in particular New Guinea and Central America (Fig. 2a). However, some regions are predicted to lose a greater proportion of their current language diversity, such as the Great Lakes region of North America, the northern Sahara and eastern Siberia (Fig. 2).

We emphasize that these predictions are not death knells, but possible outcomes in the absence of investment in language vitality. For example, while our model predicts Alutiiq (Pacific Gulf Yupik {ems}) in Alaska to increase in endangerment level, the community has instituted a language revitalization programme that may counter the predicted trend. Identifying external factors associated with language endangerment can focus attention on areas where language vitality might become threatened. For example, some areas with the greatest predicted increase in road density, such as Nigeria, Papua New Guinea and Brazil^48^, are predicted by our model to have the highest potential loss of languages (Extended Data Fig. 4). Since increasing road density also has negative impacts on biodiversity, focusing mitigation efforts on areas of increasing road density may be beneficial for both language vitality and biodiversity^49,50^.

In addition to identifying correlates of language endangerment that are likely to change in the future, such as land use, we also identify factors that are open to intervention to reduce loss of language diversity. Currently, more years of formal schooling are associated with greater rates of language endangerment (Fig. 1). Research suggests that bilingual education, where students learn part or all of the curriculum in their first language, typically results in greater overall academic achievement without sacrificing proficiency in the dominant national language^51^, but emphasis on high-stakes testing for competency in the national language can contribute to erosion of heritage language proficiency^42^. Having provision for bilingual education enshrined in legislation, or official recognition of minority languages in government or in education, is not sufficient to reduce language endangerment (Supplementary Fig. 7). Implementation requires genuine commitment to bilingual education, and support from community members who can bring heritage language to the classroom. The benefits of providing support to enhance Indigenous language vitality, in terms of wellbeing^52,53^ and socioeconomic outcomes^54^, are likely to far outweigh the costs. Implementation of support for Indigenous language vitality at all levels of governance and within speaker communities is urgent, given the predicted loss of L1 speakers who can aid language vitality and transmission (Fig. 3).

We emphasize that our analysis is focused on L1 speakers who learned the language as children, reflecting continuity of language transmission over generations. A language classified as ‘Sleeping’ (no L1 speakers) may be spoken as an acquired (L2) language in a multilingual context, as a reflection of ethnic identity or through revitalization (which may ultimately generate new L1 speakers). Language revitalization benefits from documentation, such as texts, dictionaries and grammars. Our future predictions give cause for concern that within 80 years there could be 1,500 or more languages that will no longer be spoken, yet a third of these currently have little or no documentation (Fig. 3). The majority of these languages currently have living L1 speakers, so there is still time to increase documentation based on the expert knowledge of fluent first-language speakers^55^, and to support communities to re-invigorate intergenerational language transmission^56^.

The loss of language diversity results from a complex network of factors, particularly those associated with colonization, globalization, and social and economic change. While identifying correlates of endangerment does not provide a full picture of the loss or erosion of any particular language, it does contribute to a general ‘theory of language loss’^38,57^. A key difference between species and language endangerment patterns is that while many correlates of species extinction risk are intrinsic features of species biology (such as low reproductive rate or specialist diet^58^), we have considered only ‘external’ factors, which frame the context in which languages persist. But external factors, unlike species traits, are amenable to manipulation. Some identified predictors of language endangerment may act as ‘red flags’, highlighting areas that would benefit from interventions to support language vitality (such as regions where road networks are expanding rapidly) or prompt finer-grained analysis of potential impacts (such as educational policy). Our study highlights the critical level of under-documentation of language diversity (Fig. 3), showing that without intervention, we might lose a substantial proportion of language diversity without having ever adequately documented how those languages represent unique expressions of human cultural diversity^59^. Investing in speaker communities to provide them with the support they need to encourage language diversity and vitality will bring measurable benefits in terms of social justice, community wellbeing and cultural engagement^53–55,60^.

## Methods

### Language data

We used data on L1 speakers, geographic distribution, endangerment level and relationships for 6,511 languages classified as ‘spoken L1 languages’^17,61,62^ (see Supplementary Methods for details of data and variables). We give the standard nomenclature according to the ISO 639-3 three-letter language identifiers in Supplementary Data 1, and throughout this document we give the ISO code in curly brackets at the first mention of a language. Nine ‘world languages’ were included only as factors potentially influencing language vitality (see Supplementary Table 2) but were otherwise excluded from all language-level analyses. There are several schemes for evaluating and categorizing the risk of language loss^63,64^, most of which target indicators of language vitality, such as intergenerational transmission, official recognition, domains of use, and level of documentation and resources^23,65^ (Supplementary Table 1). We based our analysis on EGIDS because it provides the most comprehensive coverage for our data (Supplementary Methods 2.1.2 and Fig. 1). Signed languages were not included in this analysis due to insufficient information on number of L1 signers, distributions and endangerment status for the majority of the world’s signed languages (Supplementary Information section 2.1.6).

Many previous analyses of global patterns of language endangerment relied on speaker population size and geographic distribution as proxies of endangerment status^4,20,66^. While low speaker number is the ultimate outcome of endangerment, current population size may not always provide a reliable indicator of language vitality or risk of loss^67,68^. Small localized languages may be stable and vigorous, for example some Papuan languages are confined to one or a few villages with only hundreds of speakers, yet are not considered endangered (for example, Neko {ISO 639-3: nej}, Mato {met}), and large widespread languages are not secure if they are not being reliably transmitted to younger generations (for example, Domari {rmt}, an endangered Indo-European language with over a quarter of a million speakers). Using population and range size to represent endangerment also conflates endangerment and diversity: range and population size correlate with number of languages per unit area^17^, so an area with more languages may, all things being equal, also contain a larger number of endangered languages^4,20^. Our analysis emphasizes global trends and general patterns over specific language trajectories or local histories. Use of global databases provides an overview of language diversity and vitality, but it is not possible to verify current speaker numbers, endangerment and distributions without expert knowledge of each individual language. Some regions or language families may be less well represented in global databases (for example, Australian languages have patchy representation and would benefit from expert revision on speaker numbers and endangerment levels). Furthermore, there is often no clear line between a dialect and a language, and this can result in variation in assigning L1 speakers to languages (Supplementary Methods 2.1.2). Our results should therefore be interpreted as providing general patterns and broad-brush predictions rather than specific detail on particular languages.

### Predictor variables

We included ten broad categories of variables to describe key extrinsic factors that influence language vitality (Table 1). Variables were either recorded per language, as a weighted average across the language area or national values, or for a 10,000 km^2^ ‘neighbourhood’ around the language (see Supplementary Methods for details). For each language, we recorded the reported number of L1 speakers, endangerment level (Supplementary Table 1), distribution^62^, level of documentation^61^, whether the language has official recognition in any country, or is officially recognized as a language of education. We characterized the ‘language ecology’ by the diversity of languages in the surrounding area, the number and proportion of endangered languages in the area, the relative representation of speakers compared to nearby languages, and whether it occurs in a country (or countries) that has one of nine ‘world languages’ as an official language (Supplementary Table 3). We recorded levels of educational attainment and education spending at national level, as well as the presence of a general provision for the use of minority languages for instruction in all or part of formal schooling, and whether each language is recognized for use in education (Supplemenary Tables 5 and 6). Socioeconomic context is represented by Gross Domestic Product per capita (GDPpc), the Gini index of income inequality and life expectancy at 60 years of age (Supplementary Tables 5 and 7), noting that these national averages do not capture variation between groups or areas within each country (see Supplementary Information 2.4).

To represent the environmental context of each language, we included variables representing population density, climate, land use, biodiversity loss, connectivity and ‘shift’ (that is, the rate of change in land use, population, built environment) (Table 1). Because language loss is often a result of language expansion replacing autochthonous languages, we included measures of connectivity: density of roads and navigable waterways (which encourage human movement) and landscape roughness and altitudinal extent (which discourage human movement). To indicate human impact on the natural environment, we included ‘human footprint’ (which summarizes anthropogenic impacts on the environment^69^) and measures of biodiversity loss. We included factors previously shown to be correlates of language diversity: mean growing season, average temperature, temperature seasonality and precipitation seasonality (we did not include species richness because biodiversity patterns are not significantly associated with language diversity above and beyond these climatic covariables^17^). To model the impact of changing landscape and environment, we included rates of change in urbanization, population density, land use and human footprint^69^.

The variables we included vary in their degree of spatial resolution. For variables concerning legislation and policy (for example, provision for minority language education), data is typically available only at country level. For some socioeconomic variables, such as life expectancy, there is regional data for some countries, but most areas only have country level data, so for consistency we used national averages provided by global organizations such as the World Bank and World Health Organization (Table 1). For environmental variables, such as temperature seasonality, we averaged values over all grid cells in the language distribution area, but for landscape factors influencing human movement, such as mountains and roads, values within the language area are not fully informative because we wish to capture movement between language areas. For these variables, we averaged over all grid cells in a ‘neighbourhood’ centred on the language distribution. For full details of the spatial resolution of each variable, see Supplementary Methods.

The variables included in this study necessarily represent current environments, socioeconomic status and contemporary policy settings. Aside from shift variables (Table 1), which represent change over time, we cannot directly capture historical processes, such as past educational programmes, historical disease epidemics, warfare or genocide. These are important factors in language endangerment but cannot be easily represented in globally consistent, universally available variables, so investigating the impact of these factors is beyond the scope of this analysis.

### Analysis

Previous analyses of global language endangerment included relatively few potential predictors and did not control for the confounding effects of both spatial proximity and relationships between languages^2,4,20,66^. Languages that cluster in space will share many environmental, social and economic features. Related languages may share not only many linguistic features but also many environmental, social and economic factors and shared historical influences^17^. All analyses rest on the assumption that datapoints are statistically independent of each other, so if we find that the residuals of the model show phylogenetic signal, then phylogenetic non-independence (when datapoints are related by descent) violates the assumption of standard statistical tests and can lead to spurious relationships^70,71^. Our method estimates the contribution of relatedness to observed patterns of endangerment, so that if there is little or no influence of relatedness on patterns of endangerment, then the phylogeny will have no effect on the outcomes^17^. A large contribution of phylogeny tells us that languages tend to be more similar to related languages in their endangerment status than they are to randomly selected languages. This does not imply that languages inherit either their endangerment status or threatening processes from their ancestors, but that relatives show patterns of similarity of endangerment^72^. If this is the case, we need to account for this phylogenetic non-independence in our analysis, so that we can identify factors that are significantly associated with endangerment above the association which is expected purely due to their shared relationships (closely related languages having more similar patterns of endangerment).

Failure to account for spatial autocorrelation can lead to false inference of patterns of language endangerment^19^. For example, socioeconomic indicators such as GDP have a strong latitudinal gradient, and so does language diversity and range size, so if range size is associated with endangerment, we would expect a significant correlation between GDP and language endangerment even if there is no direct influence of one on the other^71^. Just as repeatedly sampling two neighbouring areas but counting each observation as a unique datapoint inflates perceived environmental correlations by pseudoreplication^73^, repeatedly sampling related languages with similar cultural traits, linguistic features, historical influences and language ecologies also potentially inflates perceived associations between endangerment and environmental or social factors^19,70^. Both of these sources of covariation in the data must be accounted for to find meaningful correlates of language endangerment.

In our analysis, the dependent variable is the level of endangerment, based on EGIDS rankings (Supplementary Table 1). We are seeking global correlates of language endangerment, but we are aware that some threatening processes may have greater or lesser impact in different regions (Supplementary Methods 2.1.3). Therefore, in addition to the predictors we described above, we included an interaction term between each region and each independent predictor, to account for any region-specific effect of the predictor on endangerment. This interaction term was constructed by taking the product of the predictor and a binary variable recording whether a language belongs to the region. Any interaction term with no variation in the corresponding region was removed. Instead, we included an intercept for each region to account for differences in the average level of language endangerment among regions. In total, we have 51 predictors, 51 by 12 interaction terms, and 12 intercepts in the independent variables (Supplementary Data 3).

The basic steps of our statistical analysis are:

1. applying transformations to the 51 predictors (Supplementary Methods 4.1, Table 1), then calculating their interaction terms;
2. grouping the 51 predictors according to their pairwise correlation (Supplementary Methods 4.2) and grouping interaction terms with their corresponding predictors (Supplementary Data 3);
3. dividing the dataset into two, with two-thirds of the languages assigned to a training dataset and one-third to a test dataset. The training dataset was used to select the independent variables (candidate models) to predict current endangerment level (Supplementary Methods 4.3) and the test dataset was used to evaluate the fit of these candidate models to predict endangerment level (Supplementary Methods 4.4);
4. using the best model, re-estimating the model parameters using all 6,511 languages;
5. using the predicted change in L1 speaker population, environment and climate to generate future values of variables, then using the best model to predict future endangerment given these predicted future values (Supplementary Methods 5).

Because the dependent variable in our analysis (endangerment level) is an ordinal variable, we used ordinal probit regression^74^ to model language endangerment status. To satisfy the parallel regression assumption (that an independent variable has the same effect on threat status across all endangerment levels) for the majority of variables, we grouped recorded EGIDS scores into seven levels by combining levels 1–6a into a ‘stable’ level (Supplementary Methods 4.2 and Table 1). To account for spatial and phylogenetic autocorrelation, we constructed three matrices. The phylogenetic matrix represents relationships between languages as inferred from a taxonomy, with branch lengths scaled to relative divergence depths^17^ (Supplementary Methods 3). The distance matrix captures similarity in nearby languages due to shared environment using an exponentially decreasing function of the great-circle distance between the centroids of polygons of two languages. Since distance between centroids may not reflect on-the-ground language contact, we also used a contact matrix which contains 1 if two language polygons overlap (allowing a buffer of 100 km around each polygon), and 0 otherwise. We do not expect this contact matrix to fully capture the degree of ongoing contact between languages, which may be determined by local factors including modes of transport, form of subsistence or connectivity, but we included it to allow for an influence of close association between language distributions as an influence on patterns of endangerment, above and beyond the great-circle distances between the centres of language distributions. The distance, contact and phylogenetic matrices had zero diagonals and each row was normalized to unity. Because each matrix had its own coefficient, if patterns of autocorrelation due to distance, contact or relatedness were not important in shaping the values of variables, then the model would estimate the coefficient to zero and the matrix would not influence the result.

We then fitted an autoregressive ordinal probit model to the data. We modelled the threat status of a language as a linear function of not only the independent variables but also the threat status of other languages whose associations with the language depend on the distance, contact and phylogenetic matrices. The model was fitted to the data using a two-stage least squares approach^74^ implemented in a custom R code based on the ‘ordinalNet’ package^75^. We used a weighted sum of all the three matrices to describe autocorrelation among languages^17^. The weight was estimated by maximum likelihood using the ‘L-BFGS-B’ method^76^ in the ‘optim’ function in R.

To select the best model to predict endangerment level in our data, we first randomly divided the data into a training dataset (including 2/3 of the languages) and a test dataset (the remaining 1/3 of the languages). Then, we grouped highly correlated independent variables together and applied a stepwise selection procedure to the training dataset (see step 3) to select candidate models (details in Supplementary Methods 4.4). The procedure started with a model of a single independent variable that had the highest likelihood to the training dataset, then goes through each group (see step 2) in a random order by adding a variable of the group to the model that significantly and maximally increased model fit, and removing a variable of the group from the model that had the least and non-significant impact on model fit. These steps were repeated until there were no more variables that could be added that increased model fit, or could be subtracted without reducing model fit. This model selection procedure left us with a set of candidate models. Lastly, we measured the predictive power of each model by predicting the threat status of the languages in the test dataset and constructed the best model on the basis of its predictive power.

The best model was constructed by including predictor variables that were selected in over one-third of the candidate models which did not significantly differ in their predictive power from the model with the highest predictive power. We then estimated the coefficients of predictor variables on the complete dataset. We used this best model to predict, for each language, the probability that the language falls in each of the seven endangerment levels (combining 1–6a into one ‘Stable’ level; Supplementary Table 1). Using these probabilities, we randomly sampled the endangerment level of each language and counted the number of languages with sampled endangerment level of 2 or above (that is, EGIDS 6b–10) as the number of languages predicted to be endangered, or those in the top two levels (that is EGIDS 9–10) as the number of languages predicted to be Sleeping. This procedure was repeated 1,000 times to generate the probability distribution of the number of languages predicted to be endangered or Sleeping. We found that the expected endangerment level tends to be lower than the reported endangerment level for individual languages (Supplementary Fig. 6), but, over all the languages, the model accurately predicted the proportions of languages that are endangered and sleeping (Fig. 3).

In some cases, the mismatch between predicted and observed endangerment levels may reflect ‘latent risk’ in endangerment status^27^: languages that have characteristics typical of an endangered language, such as low L1 speaker population size, yet are rated as stable (Extended Data Fig. 1). These languages may be expected to come under increasing threat in the future. For example, Yindjibarndi {yij}, a language of the Pilbara region of Australia, has an EGIDS rating of 6a (Stable) but has a small L1 speaker population (310) and is in an area where many languages are endangered or no longer spoken. Our model predicts the expected endangerment level of this language as ‘Critically Endangered’ (EGIDS 8) at present, and without intervention to ensure language vitality, it could potentially be no longer spoken within 80 years. The reported endangerment level and the predicted probability of each language falling in each endangerment level at present, in 40 years and in 80 years are listed in Supplementary Data 4.

### Future prediction

We used the best model of current language endangerment status to predict possible future changes in endangerment status for our global database of languages. Current EGIDS levels give us information on intergenerational transmission, so we can use that information to model declining L1 speaker population: if a language is currently only spoken by adults and not transmitted to children, then, without revitalization, there will be no more L1 speakers once the current speakers die. EGIDS also indicates which languages are declining in L1 speaker population so we can model the probable decline in numbers in 40 years (2060) and 80 years (2100; Supplementary Methods 5.2.1). These models predict possible patterns of language loss in the absence of revitalization programmes that might increase the number of L1 speakers, by assuming that without intervention to improve language transmission and vitality, endangered languages will undergo demographic shift that changes endangerment level, as described in Supplementary Methods 5.1 and Table 7. These predictions are conservative in the sense that they assume that languages that are not currently endangered will remain stable into the future. We emphasize that this procedure is specifically modelling the shift in number of first language (L1) speakers of a language, not the population they belong to. A population may thrive and its ethnic identity remain strong even if speakers shift to a different language. To model the L1 speaker population size, we need to consider generational transmission of the language (that is, are children learning it as their first language?), rather than the number of people in the population that they belong to.

For example, if a language with an EGIDS level of 6b (Threatened) is predicted to be Endangered (EGIDS level 7) in the future on the basis of having no child L1 speakers, we adjust the probability distribution of the endangerment level predicted by the model for the language at that timepoint by shifting the probability distribution one level up, setting the probability that the language has an endangerment level lower than Endangered to zero, and renormalizing the probability distribution. We then randomly sample the endangerment level of each language, and count the number of languages overlapping each hex grid that are Endangered or Sleeping. This procedure is repeated 1,000 times to get the probability distribution of the number of languages predicted to be endangered or sleeping in each hex grid. We plot the combined predictions on a map, showing both the expected value of the number of languages per grid that are endangered or sleeping in 40 and 80 years, and also the proportion of languages per grid that are Threatened, Endangered or Sleeping. In the Supplementary Information, we demonstrate how this predictive model can be extended to incorporate future values of predictor variables, such as changing climate or land use.

### Reporting Summary

Further information on research design is available in the Nature Research Reporting Summary linked to this article.

## Supplementary information

Supplementary Information Supplementary Information.

Reporting Summary

Peer Review Information

Supplementary Data Supplementary Data 1–4.

## Data Availability

All variables analysed are provided in Supplementary Data. These variables are derived from a range of sources, as cited in the text and in Table 1 (most of these data are freely available but some are under license).

## Code availability

Code for data preparation is available at https://github.com/rdinnager/language_endangerment. Code for running the analysis is available at https://github.com/huaxia1985/LanguageEndangerment. The custom R code includes functions that modify functions in the ‘ordinalNet**’** R package to correct for autocorrelation in ordinal probit regression.
