# Publisher Correction: Global predictors of language endangerment and the future of linguistic diversity

**DOI:** 10.1038/s41559-022-01684-4

**Published:** 2022-02-03

**Authors:** Lindell Bromham, Russell Dinnage, Hedvig Skirgård, Andrew Ritchie, Marcel Cardillo, Felicity Meakins, Simon Greenhill, Xia Hua

**Affiliations:** 1 Macroevolution and Macroecology, Research School of Biology, Australian National University, Canberra, Australian Capital Territory Australia; 2 ARC Centre of Excellence for the Dynamics of Language, Australian National University, Canberra, Australian Capital Territory Australia; 3 Department of Linguistic and Cultural Evolution, Max Planck Institute for the Science of Human History, Jena, Germany; 4 ARC Centre of Excellence for the Dynamics of Languages, School of Languages and Cultures, University of Queensland, Brisbane, Queensland Australia; 5 Mathematical Sciences Institute, Australian National University, Canberra, Australian Capital Territory Australia

**Keywords:** Language and linguistics, Macroecology

Correction to: *Nature Ecology & Evolution* 10.1038/s41559-021-01604-y, published online December 2021.

This paper was originally published under standard Springer Nature license (© The Author(s), under exclusive licence to Springer Nature America, Inc.). It is now available as an Open Access paper under a Creative Commons Attribution 4.0 International license, © The Author(s). The error has been corrected in the HTML and PDF versions of the article.

Open Access This article is licensed under a Creative Commons Attribution 4.0 International License, which permits use, sharing, adaptation, distribution and reproduction in any medium or format, as long as you give appropriate credit to the original author(s) and the source, provide a link to the Creative Commons license, and indicate if changes were made. The images or other third party material in this article are included in the article’s Creative Commons license, unless indicated otherwise in a credit line to the material. If material is not included in the article’s Creative Commons license and your intended use is not permitted by statutory regulation or exceeds the permitted use, you will need to obtain permission directly from the copyright holder. To view a copy of this license, visit http://creativecommons.org/licenses/by/4.0/.

