# Supplementary material for: Global predictors of language endangerment and the future of linguistic diversity
Source: Nat Ecol Evol. 2021 Dec 16;6(2):163–73. doi: 10.1038/s41559-021-01604-y (PMC8825282; doi:10.1038/s41559-021-01604-y)

nature portfolio

https://doi.org/10.1038/s41559-021-01604-y

Supplementary information

Global predictors of language endangerment and the future of linguistic diversity

In the format provided by the authors and unedited

Global predictors of language endangerment and the future of the world's linguistic diversity

Lindell Bromham*, Russell Dinnage, Hedvig Skirgård, Andrew Ritchie, Marcel Cardillo, Felicity Meakins, Simon Greenhill, Xia Hua

* Corresponding author: Lindell Bromham, Lindell.Bromham@anu.edu.au

---

Supplementary Information

1. Language endangerment scales
2. Language variables
    2.1. Language
        2.1.1 Language identity
        2.1.2 L1 speaker population size and endangerment level
        2.1.3 Region and island languages
        2.1.4 Official languages
        2.1.5 Documentation
        2.1.6 Signed languages
    2.2. Diversity
    2.3. Education
    2.4. Socioeconomic indicators
    2.5. Land Use
    2.6. Environment
    2.7. Biodiversity loss
    2.8. Connectivity
    2.9. Shift
    2.10. World languages
3. Hierarchy of relatedness
4. Statistical analysis
    4.1. Predictor transformations
    4.2. Regression model
    4.3. Candidate models
    4.4. Model selection
    4.5. Model outcomes
5. Future prediction
    5.1. Generational shift
    5.2. Model predictions

5.2.1. Demographic shift in L1 speaker population

5.2.2. Climate change

5.2.3. Environmental change in land-use and built environments

6. Supplementary References

List of Supplementary Tables

Supplementary Table 1: Language endangerment scales

Supplementary Table 2: World languages

Supplementary Table 3: Official languages

Supplementary Table 4: Additional sources of socioeconomic and education data

Supplementary Table 5: Legal provision for minority education.

Supplementary Table 6: World Bank indicators

Supplementary Table 7: Models for predicting patterns of future language endangerment

Supplementary Table 8: Demographic data used in future predictions

Supplementary Figures

Supplementary Figure 1: Relationship between language endangerment scales

Supplementary Figure 2: Endangerment scales and L1 speaker population size

Supplementary Figure 3: Variables included as predictors in our analysis of language endangerment

Supplementary Figure 4: Pairwise correlation coefficients between variables

Supplementary Figure 5: Relative number of languages rated as endangered or Sleeping by region

Supplementary Figure 6. Comparison of observed and predicted levels of endangerment

Supplementary Figure 7: Predictor variables identified in best-fit models

Supplementary Figure 8: Predicted patterns of language endangerment under generational shift, demographic transition, land-use shift and climate change.

1. Language endangerment scales

A number of language endangerment scales have been developed (Supplementary Table 1). No endangerment scale is perfect, but we have chosen to base our analysis on scores on the Expanded Graded Intergenerational Disrupted Scale (EGIDS) because it has the most comprehensive coverage for global languages (Supplementary Figure 1), and it incorporates many factors in its endangerment levels[1]. EGIDS is an ordered 13-point scale, ranked 0 to 10 with subdivisions of levels 6 and 8[2,3]. This scale is based on number of L1 (first language, or "mother tongue") speakers, domains of use (e.g. government, trade, education, home), intergenerational transmission (e.g. whether being actively learned by children), official recognition and stability (whether the language is stable or declining: Supplementary Table 1). We refer to languages with a score of 6b or above as "endangered languages".

UNESCO uses a scale that includes nine different categories of information, including speaker population size, intergenerational language transmission, proportion of speakers within the total population, community attitudes, shifts in domains of use, educational materials and documentation, institutional recognition and government policies, expressed in six levels spanning from safe to vulnerable to endangered to extinct[4]. The UNESCO scale does not have as wide a coverage as EGIDS, with over a quarter of languages in our dataset lacking a UNESCO score (Supplementary Figure 1). In particular, scores are only provided for languages identified as endangered, therefore languages that are not included in the database might either be safe or not assessed (see http://www.unesco.org/languages-atlas/en/statistics.html). Given the potential for some endangered languages to be unassessed and therefore not included in the data, this makes the UNESCO rankings unsuitable for our analyses.

The Language Endangerment Index (LEI) used in the Catalogue of Endangered Languages ElCat[5] is based on four factors: intergenerational transmission, domains of use, absolute number of speakers and whether the population is increasing or decreasing[6]. LEI also incorporates a certainty measure, which reflects on whether reliable information is available in each of the factors[7]. However, ELCat focuses on endangered languages, and does not provide information on languages that are widespread and secure. Therefore LEI has incomplete coverage for our data, with over a quarter of the languages in our dataset lacking a LEI score (Supplementary Figure 1).

The Agglomerated Endangered Scale (AES) used in Glottolog[8] is a conglomerate of the other three scales described above, preferencing the available rankings of LEI first, then UNESCO, then EGIDS[9]. While this has similar coverage to EGIDS, its current implementation in Glottolog makes it inappropriate for use in this analysis. Specifically, language ISO 693-3 codes that contain a number of sub-categories (such as dialects) are in many cases assigned the highest threat level from the daughter nodes. In some cases, this results in languages with large numbers of speakers being classified as extinct or nearly extinct (Supplementary Figure 2).

While the four language endangerment scales considered here use similar information and largely agree on the status of the bulk of languages (Supplementary Figure 1), they do produce different ranks for some languages. For example, the Indo-European language Pangwali [ISO639-3, pgg] was simultaneously rated Vigorous (6a) in Ethnologue (using EGIDS), Shifting (7) in Glottolog (using AES), Threatened in ElCat (using LEI) and Critically Endangered by UNESCO.

We do not use "IUCN-style" endangerment ratings based on population size, range size and decline[10,11] to avoid circularity and conflation with diversity[12]. Instead, we include speaker population size and language area in our analysis as variables.

2. Language data

Table 1 lists the variables included in this study. Some variables are based on available information for each language, such as level of documentation or official status, but most of the variables are estimated using spatial information in the form of language polygons which describe the area that a language is reported to be distributed in World Language Mapping System v17 (WLMS, http://worldgeodatasets.com). In the case of variables collected at country level (see Figure 1, Table 1), the polygons are used to identify the country or countries the language distribution overlaps with. For environmental variables, such as "human footprint" or temperature, the values are averaged over all grids in the polygon/s. For connectivity and language ecology variables, the polygons are used to define a neighbourhood around the language for which the values of the variable are calculated. Further details of all variable estimation given below. We give the standard nomenclature of ISO 639-3 three letter language identifiers throughout this document at the first mention of a language, identified in square brackets, for example Gurindji [gue] or Samoan [smo].

The ISO 639-3 code for every language included in our analysis is included the raw data provided in Supplementary Data 1.

Polygon-aggregated variables were based on data in the form of rasters, that is, two-dimensional grids of cells covering the surface of the Earth, each containing a value representing the area they cover. These rasters were obtained from various sources (see Table 1). Values for individual languages were calculated by taking the mean value of every raster grid-cell that fell within a language's polygon/s. This was done using the R package *velox*[13], which uses an efficient method of extraction that can deal with polygons that are smaller than a raster's grid-size.

Some languages that were characterised as "widespread" have polygons outside their country of origin – these were removed to prevent their environmental variables being skewed by large polygons in countries where the language was not native. An example of this was Tagalog [tgl], an Austronesian language of the Philippines that had polygons representing speakers in Canada and the United States of America. Languages classified this way were rare in the WLMS data, and so this affected only a relatively small number of languages, including languages that we classify as "world languages" (see below) that are not included in the analysis except as independent variables. The ISO 639-3 codes of languages affected are: fui, arb, aii, hye, acq, bho, afr, rmt, ase, nld, por, eng, fra, spa, hau, deu, kdh, nqo, hak, gil, srp, zul, gdq, urd, tam, fue, yue, prs, cmn, nan, rus, yor, swh, pov, hrv, dyu, fuv, vie, ita, bos, rup, pbu, zsm, kor, kvk, ajt, uzn, jpn, ukr, pol, fng, ydd, bel, zlm, tgl, dan, guj. WLMS records a "HUB" variable indicates the region that is considered the language's "home region". We used this to remove any polygons that did not occur in a language's home region. After this filtering, some languages still have multiple polygons, in which case we use the union of all polygons for a language in subsequent analyses. These in most cases were languages which occur in multiple countries, which WLMS splits by country borders, or that occur on multiple islands.

We calculate the area (in km^2) of the language polygon in the WLMS (WLMS 17, worldgeodatasets.com). The World Language Mapping System removed spatial data relating to languages classified as "extinct" from the 17th edition onwards. Therefore, we used data from the 16th edition on the spatial distribution of "extinct" languages, which we combined with spatial data from the 17th edition for all other languages.

Not all languages in the WLMS have polygon data available: some are represented by a single point location. For the 706 languages with only point locations, we derived a polygon using a Voronoi tessellation approach, in order to allow us to derive variables at the polygon, neighbourhood, and national level for all languages with location data. Specifically, we took a single point for each language in the dataset (where that point was the centroid for languages with a full polygon) and generated a Voronoi tessellation based on those coordinates. We then calculated the intersection of each Voronoi cell and global coastlines such that no portion of any language's range fell in the ocean. We then assigned this intersected Voronoi polygon to those languages in WLMS which are missing polygon data, while retaining the original WLMS polygons for languages which already had polygon in WLMS. All subsequent analyses are based on these language polygons (i.e. from WLMS where provided or Voronoi projection where WLMS only gives a point location). Note that national level values are calculated as a weighted average for any countries that a language polygon overlaps, so for languages whose distribution was given only as a point location in WLMS, the national level variables will be calculated as a weighted average over any countries the Voronoi projection of the distribution overlaps.

National-level variables, such as socioeconomic measures, were aggregated to a single value for each language by calculating an area weighted average across a language's polygon(s). More specifically, we calculated the intersection between each language's range polygon(s) and each country's polygon(s), where country polygon data was extracted from the Natural Earth dataset (https://www.naturalearthdata.com/). The language's value was then calculated as the mean value for a variable across all countries it intersected with, weighted by the proportion of its area accounted for by each intersection. National level data was linked to Natural Earth spatial polygons with their ISO alpha-3 codes (ISO 3166-1).

Neighbourhood-based variables, such as road density and landscape roughness, were also based on data in the form of rasters. However, instead of being aggregated across a language's range polygon(s), they were aggregated across a language's "neighbourhood", defined as a 10,000 km^2 circle centred at the language's range polygon centroid (that is, a circle with a 56km radius around the language centroid, or, for languages with multiple polygons, around the centroid of the largest polygon). The neighbourhood was designed to capture aspects of the "ecology" of a language and was used for variables relating to a local language diversity and connectivity. While any such designation must be arbitrary, the size of the neighbourhood was chosen to provide an appropriate spatial distribution and scale

given the granularity of the environmental variables, and the area over which people might be expected to come into contact with neighbouring populations (representing roughly a day's journey on foot from the centre of the neighbourhood to the edge, though this will of course depend on landscape and cultural norms). The circle was created in spherical geometry, such that it was independent of any spatial projection, and to ensure exactly equal area across all parts of the globe, free from projection distortion. Values for Neighbourhood variables had data in the form of rasters, and values for individual languages were calculated as the average value of all grid-cells that fell within the neighbourhood circle, in the same way as described above for polygon-aggregated raster-based variables.

All spatial intersections described above were done by first converting each dataset to a common cylindrical equal-area projection (EPSG:6933), before calculating their intersection using the *sf* package in R[14]. All other spatial operations mentioned in these methods likewise were conducted in this projection, except where it was possible to use spherical geometry operations (e.g. calculation of areas, perimeters and lengths).

2.1 Language

2.1.1 Language identity

Ethnologue uses the ISO 639-3 code standard (which is administered by Ethnologue's publisher Summer Institute of Linguistics). Glottolog applies their own classification schema and language codes (glottocodes). These two sets of language standards, ISO 639-3 and glottocodes, are similar and agree in most instances. Glottolog also maintains mapping between glottocodes and ISO 639-3 codes, making it possible to combine data from Glottolog and Ethnologue. We used Glottolog's mapping schema to match data between the two sources. Both Ethnologue and Glottolog differentiate between languages and dialects.

We record the ISO 639-3 code for each language (see Supplementary Data 1), and use the ISO 639-3 mapping in Glottolog to identify spoken languages[8]. For our analysis, we include only languages classified in Glottolog in the "L1 spoken language" category. This category should exclude sign languages, artificial languages, most pidgins, speech registers, ritual languages, cants and languages within the Glottolog categories unattested, unclassifiable and Bookkeeping. The exclusion of these languages should not be taken as a sign that these categories of languages do not deserve the attention of those concerned with language

diversity and endangerment, but instead is an indication that many of these language types cannot easily be included in a global analysis along with spoken L1 languages. For example, signed languages are an important part of linguistic diversity and insufficient attention has been paid to patterns of endangerment[15], but it would be problematic to include signed languages here they do not have equivalent data to spoken L1 languages that allows us to include them in the same analytical framework: the phylogenetic position is difficult to express within the standard 'genetic' relationships, accurate L1 numbers can be difficult to obtain, and distribution data can be problematic given that many signed languages are spoken by communities embedded with the distribution of other spoken languages. In addition, it is likely that signed languages face a different set of pressures that are distinct from the processes endangering spoken L1 languages, such as access to signed education vs oral-only teaching, whether parents use sign language or not, availability and use of cochlear implants and so on. See section 2.1.6 for a more detailed discussion of the limitations that prevent the inclusion of signed languages in this analysis.

Nine world languages (Table 1) are not included as languages in the analysis, but as variables – see section 2.10 for details. In total, this results in a dataset with 6511 languages.

2.1.2 L1 speaker population size and endangerment status

Number of L1 (first language) speakers is an essential variable for an analysis of language endangerment, but it is also difficult to obtain accurate current estimates. Reported L1 speaker population sizes can be derived from a range of sources and can vary at different points in time, so there may be several conflicting reports for some populations. Census data may sometimes conflate ethnic identity with L1 speakers, as language often aligns with cultural affiliation[15,16]. Given the difficulty of obtaining accurate estimates, no database of reported L1 speaker population sizes will be perfect, and because L1 population sizes for many languages are subject to ongoing change, they can at best represent a "snapshot" of speaker populations at a particular point in time. Furthermore, L1 speakers can be difficult to define for languages that occur within a dialect continuum, and within Ethnologue there are cases where the reported L1 is zero but the EGIDS score is 6a. For example, Shendu [shl], part of the Shö language dialect cluster of Burma and Bangladesh, is rated Vigorous (6a) presumably because there are 50,000 speakers across the dialect cluster, but the number of speakers of the Shendu dialect is not known and is listed as zero in our dataset. L1 speaker population will not always be an adequate indicator of language vitality, for

example Bangala [big] is an African "market language" with millions of speakers, but it has a reported L1 of zero because it is rarely if ever learned as a first language. We cannot include L2 (second language speakers) in our analysis because accurate L2 numbers are available for very few languages, and may not be comparable across languages, depending on the evaluation of second language competency. L2 numbers cannot be easily extrapolated from L1 figures (see, for example, Supplementary Table 2). Therefore, it is important to emphasize that "L1 speaker population" is not equivalent to "number of fluent speakers" of a language. Our results should therefore be interpreted as investigating the predictive power of reported L1 speaker population sizes as an indicator of language vitality and intergenerational transmission.

The L1speaker population size were predominantly taken from the 17th edition Ethnologue, with some adjustments. Firstly, some languages listed as "extinct" do not have L1 population sizes reported in e17 so we obtained those L1 population sizes from e16, the last version in which they were included. Secondly, we corrected a small number of L1 values that were at odds with other sources of information. Accurate recording of L1 speaker population sizes is very challenging. We examined 142 languages which had usually high or low L1 population for the endangerment category they were assigned to: specifically those languages having a EGIDS rating of 4 or lower (not endangered) and a population below 31,400 or with an EGIDS of 7 or higher (endangered) and a population of 31,400 or higher. We found that in most cases the reported L1 estimates were similar to those reported in other sources, but there were seven cases where we decided the evidence was strong enough to use another source for the population statistics. The adjusted L1 values were for the following languages: Congo Swahili [swc], Dendi [ddn], Semai [sea] (new figures from the 23rd release of Ethnologue); Mussau-Emira [emi] (using figure from UNESCO[4]; Djambarrpuyngu [djr] (using figure from the AUSTLANG database: https://collection.aiatsis.gov.au/austlang/); Iyo'wujwa Chorote [crq] (using data from Crevels[16]); Temiar [tea] (data from Benjamin[17]).

The endangerment level of each language was taken the Expanded Graded Intergenerational Disruption Scale (EGIDS) as reported in Glottolog (v4.2.1). EGIDS is primarily determined by domains of use (mass media, international policy, school, etc) and intergenerational transmission (whether currently learned by children and the age of most L1 speakers: Supplementary Table 1). For example, a language with an EGIDS value of 1 is used in government, mass media, education and business, such as Lao [lao], Amharic

[amh] or Swedish [swe]. The highest value, 10, indicates that the language is currently not spoken, for example Siraya [fos], a Formosan language from Taiwan that was spoken until two centuries ago, and Tillamook [til], a Salishan language from Oregon whose last first-language speaker died in 1972. Often such languages are referred to as "extinct", but we do not use that term here. Instead, we use the term "Sleeping" as it is preferred by many in the endangered languages community[18-20], because as it is possible for a language that has no L1 speakers to be revitalised, particularly where there is strong community support and sufficient linguistic resources to support language revitalisation. For example, Kaurna [zku], a Pama-Nyungan language of the Adelaide area in Australia, was probably last spoken on an everyday basis in the 1860s. The last L1 speaker of the Kaurna language died in 1929. In the last two decades, the Kaurna community have galvanised to revitalise their language in collaboration with linguists through school language programs, adult language learning classes, the development of a learner's guide and other teaching resources, radio shows and increased visibility through 'Welcome to Country' speeches and reinstating Indigenous place names (see www.adelaide.edu.au/kwp).

While a language's EGIDS value often reflects number of L1 speakers (Supplementary Figure 2), it is not fully determined by speaker population. For example, Inari Saami [smn] is recognised as a minority language in Finland and therefore has the EGIDS status of 2 (Provincial) although it has a reported L1 speaker population of 300. EGIDS is typically aligned with other language endangerment scales (Supplementary Figure 1), but not always, for example Inari Saami is rated "Shifting" in the AES.

2.1.3 Region and island languages

Patterns of language endangerment vary over the world due to local or regional influences, for example due to different patterns of colonization or differing threatening processes. To account for potential difference in the effect of each independent variable on languages in different continents, we assign each language to one of 12 global regions using the Natural Earth dataset (https://www.naturalearthdata.com/). The 12 regions are chosen by grouping regions in the dataset, so that each region has roughly even number of languages (Figure 1). Languages were assigned to a region in the Natural Earth dataset by calculating an area of overlap between the language's polygons and the country polygons in the Natural Earth dataset, and assigning the region associated with the country of highest overlap with the language. For a small number of languages (34), no overlap was detected (these were generally small near-shore or island languages, and the lack of overlap occurred

due to minor misalignment between the spatial datasets, likely induced by reprojection to a common projection). For these languages, we assigned the region of the country nearest to the language's polygon.

Note that this division of the world into regions is based on both geography and political sovereignty (Figure 2), both of which may contribute to factors that impact on language endangerment. For example, Papuan languages occurring on the east side of New Guinea are counted in Melanesia, while those on the west side, being politically a part of Indonesia, are counted in South East Asia. Some languages are therefore assigned to regions that seem odd for their location and cultural affinities. Two examples from far-flung ends of the great expanse of Austronesian languages illustrate this point. The Malagasy language Kibosy Kiantalaotsy-Majunga [buc] is an Austronesian language spoken on the island of Mayotte in the Comoros Archipelago, which is under French rule, so in the Natural Earth Dataset this language is assigned to "Western Europe". Similarly, the Austronesian language Rapanui [rap] is assigned to "South America" because Rapa Nui Island (Easter Island) is a special territory of Chile. Given such ambiguities, any set of rules will involve some decisions on arbitrary cut-offs in defining regions, so we have chosen a method that produces the most sensible outcomes in the majority of cases.

Island languages have previously been reported as having higher levels of language endangerment, just as island species are often more vulnerable to extinction[21]. Therefore we wish to ask if languages on islands have greater levels of endangerment, above and beyond covariation with other predictors, and after correcting for relatedness and spatial non-independence. Since there is a continuous range of landmasses of difference sizes, there is no absolute definition of what constitutes an "island" (for example, should Tasmania be considered an island for the purposes of language endangerment? If so, then should Sri Lanka be considered an island, because it is approximately the same size? And so on). Therefore, identifying island languages requires us to specify a cut-off land area, which must necessarily draw an arbitrary line between landmasses of different sizes. We designated languages as island languages using the following method: we calculated the intersection between every language range polygon and every Natural Earth dataset polygon (from their highest resolution dataset), each representing an individual landmass. We then designated the language as an island language if the maximum intersection area among all its intersections was less than 10,000 km^2. In other words, if the largest landmass a language is found on is less than 10,000 km^2, we consider it an island language, and it is

marked '1' in the database, otherwise it is marked as '0' for language. This definition of island is similar to Tershey et al (2015) who used 0.11ha as their cut-off for islands, based on the Global Island Database. However, any size chosen as a cut-off will lead to some arbitrary classifications. For example, Fijian [fij] is assigned 0 for island because the largest island in Fiji, Viti Levu, is 10,389 km^2.

Because we define island languages based on the largest landmass the language occurs on, some languages that are predominantly found on a small island but have some speakers on a larger landmass will be given a "0" for island. For example, Mawng [mph] is an Iwaidjan language primarily spoken on the Goulbourn Islands in the Arafura Sea, off the north coast of Australia, but there are also speakers in Arnhem Land on the nearby mainland, so it is not designated as an island language in this dataset. While Ambonese Malay [abs] is associated with the island of Ambon, it has speakers on North Maluku (which is greater than 10,000km^2), so is not designated an island language. Similarly, Ternatese [tft] and Tidore [tvo] are Papuan languages from Ternate and Tidore islands in the Moluccas, but they also have speakers spoken on North Maluku. Our definition of island language is therefore similar to the biological concept of an island endemic species as a species found only on islands and not on any larger landmasses.

2.1.4 Official languages

To account for the effect of constitutional recognition on language vitality, we record which languages are officially recognized at the national level for each state (and, in the case of some countries without an official language, the primary language of government, commerce and education). It is difficult to come up with a definitive list of official languages, so any such list will be imperfect. Official languages change over time, for example South Sudan has recently replaced Arabic [ara] with Swahili [swa] as an official language (in addition to English [eng]). Some countries do not formally recognize an official language, even if there is a clear predominant language. For example, Australia does not have an official language but English is the main language of education, commerce, media and government, so to reflect its wide sphere of influence, we record English as a *de facto* official language for Australia. In other cases, languages may have official constitutional recognition even though they have limited number of speakers or restricted domains of use. For example, Bolivia recognizes 36 official languages, some of which have very few speakers (e.g. Baure [brg], Itonama [ito], Leco [lec] and Guarasu'we [psm]) or have culturally restricted usage patterns (e.g. the ritual language Machajuyai-Kallawaya [caw] which has

no L1 speakers). In Supplementary Table 3, we record the official languages at national level. Note that we use "national" as a convenient but nonpartisan designation for political units including nations, semi-autonomous regions, territories and so-on, without making any statement about recognition of those states by other jurisdictions. All national-level data is expressed as a value for each language by taking a weighted average of the overlap between the language polygon/s and country borders (from the Natural Earth dataset https://www.naturalearthdata.com).

2.1.5 Documentation

Traditionally, good language description is considered to consist of what is now known as the 'Boasian trilogy', including a grammar, a dictionary and a collection of texts, forming the descriptive canon of a language[22,23]. In particular, the text collection offers a culturally-rich and more community-orientated approach to the lexical and grammatical descriptions. More recently the descriptive enterprise has transformed into the practice of 'language documentation' where the Boasian trilogy is now supplemented by an audio-visual corpus in which recordings are transcribed, translated and annotated using various types of software, which aims to create an enduring record of the linguistic practices and traditions of a speech community[24]. Good language documentation consists of recordings from a range of genres which represents a range of cultural practices, for example narratives (e.g. oral histories, sacred texts), elicitation (e.g. words, sentences, sound contrasts), commentaries (recounting news, a story, film, game), definitions or explanations, dialogues/conversations (greetings, meal-time discussion, socialising) and procedurals ("how to make…")[25]. Languages which are well documented have the resources required for language maintenance work, for example school programs, signage, translation work from major regional languages etc. Documentation can help to give a language political status when communities are petitioning to have their languages recognized officially. For example, Lavukaleve [lvk] is an Austronesian language spoken by around 2000 people in the Central Province of the Solomon Islands. Although very few people are literate in Lavukaleve, the community was strongly in favour of producing a dictionary because it added prestige and status to the language. As Terrill (2002: 217) noted "(a)cknowledging and utilizing powerful cultural symbols like this [dictionaries] is a useful way to encourage language maintenance, and it is one way to halt the pattern of language endangerment that is creeping into some communities"[26]. Documentation can also play a key role in language revitalization[25,27,28].

We use information on level of documentation from Glottolog (v4.2.1). Languages in Glottolog are ranked from having the most extensive kind of description (a full grammar) to the least (for example, only mentioned in an overview of an area). See Hammarström et al.[9] for more details on the documentation level parameter of Glottolog. We aggregate this data to three levels: 0 = little or none (no documentation recorded or only basic wordlist); 1 = basic (e.g. phonology, textual material, grammar sketch); 2 = detailed (e.g. long grammar).

2.1.6 Signed languages

As discussed in section 2.1.1, this analysis only includes languages designated "Spoken L1 languages". Why are signed languages not included in this analysis? Signed languages are an important part of linguistic diversity, and the study of sign languages is an essential component of our understanding of human languages[29,30]. Signed languages show a wide range of variation in patterns and domains of use: some are recognised as national languages (e.g. Korean Sign Language [kvk]), others have very localised use (for example Ban Khor Sign Language [bfk], spoken in a single rural village in northeastern Thailand)[30]. Many signed languages have L1 speakers who learned it as their first language, predominantly Deaf children and their families. Some signed languages are used widely by both Deaf and hearing people, adding to a community's linguistic repertoire[30,31]. Some may be primarily used by hearing people in specific cultural domains of use, or used when spoken language is not practical or appropriate[31,32]. Many signed languages are endangered and some are no longer learned as a first language[15,29].

Just as it is valuable to identify global and regional correlates of endangerment status in spoken languages, it would be illuminating and useful to examine factors increasing the risk of endangerment of signed languages. But signed languages cannot be included in this global analysis of language endangerment for several reasons, which we summarise here:

<u>(1) Poor representation in the available database</u>: Glottolog lists 291 signed languages. 150 signed languages are listed in the latest version of Ethnologue, possibly because not all of the sign languages listed in Glottolog have an ISO 693-3 code. For example, Glottolog recognizes seven different Zimbabwean sign languages (including Manicaland Sign, Mashonaland Sign, Masvingo School Sign, Matabeleland Sign, Midlands Sign, Zimbabwe Community Sign, and Zimbabwe School Sign) but of these only Zimbabwe Sign Language [zib] has an ISO 693-3 code. Both the Glottolog and Ethnologue lists of signed languages

are likely to be an incomplete and geographically biased sample of the worlds signed languages. For example, there are at least 20 Indigenous sign languages in Australia[32], but only four are listed in Ethnologue.

The basic data required for our analysis is patchy for signed languages. For around half of the signed languages listed in Ethnologue, the number of L1 signers is given as zero (77 out of 130 signed languages with a record for L1 users), however this is unlikely to indicate that all of those languages are no longer learned by children. For example, Ho Chi Minh City Sign Language (or Saigon Sign Language, [hos]) is listed as having zero L1 signers yet it is used by many Deaf communities in Vietnam. There has not been a formal census of Deaf people in Vietnam[33], therefore the record of zero L1 signers for Ho Chi Minh City Sign Language is likely to reflect a lack of information rather than a lack of first-language signers.

Information on levels of endangerment is also patchy for signed languages. Of the signed languages listed in Glottolog, nearly half have no recorded endangerment level, around a quarter are listed as Not Endangered, and the remaining signed languages in Glottolog are in AES categories Shifting (45), Threatened (18), Nearly Extinct (9), Moribund (4) and Extinct (3) (See Supplementary Table 1 for AES scale).

<u>(2) Sparse and non-equivalent distribution data</u>: Distribution data is very limited for signed languages in the databases we have used in this analysis. In the WLMS database, only 11 signed languages have distribution polygons, 29 have point location data, and 91 are designated "widespread". Without distribution data, we cannot derive the majority of variables in this analysis (see Table 1). This paucity of distribution data may be partly due to the difference in the nature of distributions of L1 signed languages. For most spoken L1 languages, the distribution data represents the area over which it is the predominant first language. Although some signed languages are learned as a first language for all people in an area, signed languages typically overlap completely with one or more L1 spoken language distributions. For example, Yolgnu sign language [ygs] is learned at an early age by both Deaf and hearing people on Galiwin'ku (Elcho Island, northern Australia), where it is used in daily communication[31]. Most signed languages are learned as a first language by only a small fraction of their community, and all overlap autochthonous spoken languages in distribution. For example, the Australian sign language Auslan [asf] is a first language of approximately 10,000 people, and its distribution is over the entire Australian continent.

Therefore, the distribution of Auslan overlaps with the distributions of hundreds of Indigenous languages although it represents on average less than 0.04% of the population. We can't use Voronoi projection to infer signed language distributions, because this procedure assumes that the area of L1 speakers around a location point will be determined by the boundaries of other L1 spoken languages, which is clearly not the case for many signed languages.

(3) Different threats to vitality: while some of the variables included in this study may be as relevant to signed languages as spoken languages, such as GDP, most variables in this study have been specifically chosen to represent threats to spoken languages. In addition, the vitality of signed languages can be threatened by unique factors, such as rates of congenital deafness and medical interventions. For example, Auslan is considered endangered ("Shifting" in the AES endangerment scale, equivalent to EGIDS 7: Supplementary Table 1), but the factors influencing decline in L1 speakers are distinctly different from other endangered Australian languages, including the declining rates of rubella infections[34] and use of hearing aids and cochlear implants in infants[35]. In some cases, the threats to language vitality may be similar in nature, such as adoption of a national standard language in education, and yet the variables reflecting this pressure for spoken languages might not capture the same process for signed languages. For example, legal recognition of minority language may specifically include signed languages (such as Canada's 2019 Accessible Canada Act and Indigenous Languages Act), while some specifically apply only to spoken languages (e.g. in the USA, Native American Languages Act 103.6 says "The term "Native American language" means the historical, traditional languages spoken by Native Americans."), but most make no specific mention of signed languages. Therefore we can't assume that variables representing legal recognition of minority languages apply equally to signed and spoken languages.

(4) Non-equivalent phylogenetic data: In both Glottolog and Ethnologue, signed languages are gathered in a single group, or related groups, within the taxonomic hierarchy (referred to in Glottolog as a Pseudo Family). In other words, they are grouped by modality, not by their origins and relationships. There is some structure within the Deaf Sign Language Pseudo Family, for example Auslan, Papua New Guinean Sign Language [pgz], British Sign Language [bfi] and New Zealand Sign Language [nzs] are grouped together to reflect their shared origin. But most of the sign languages are in a "star phylogeny" where there is no hierarchy of relatedness, and no relationship to the other autochthonous

languages in the same area. A star phylogeny could be considered a fair representation of the emergence of small-scale “village sign languages”, which can develop spontaneously without being derived from an related signed language[29], but only if they show no similarity through relatedness to any other language, including local spoken languages. But a star phylogeny may not always be a fair representation of expected degree of similarity amongst signed languages and between signed and spoken languages. Since signed languages have a very different phylogenetic representation in these databases than spoken languages, they cannot easily be combined in the same analytical framework.

In summary, endangered signed languages have received much less attention than endangered spoken languages[15]. However, representation of the world's signed language diversity and vitality in global databases is currently too patchy to allow signed languages to be included in the current analysis, as the majority of signed languages do not have data on number of first language (L1) signers, geographic distribution or endangerment status. Furthermore, it would be desirable to analyse signed languages separately from spoken languages since they differ in the nature of their relationships to other languages, the form of their distributions (usually embedded within other language distributions), and in the threats to their vitality which may be specific to signed languages and not relevant for spoken languages (for example, reduction in L1 signers with the rise in medical interventions).

2.2 Diversity

There are two reasons to include variables that describe the relative representation of other languages in the vicinity of each language. Firstly, we need to distinguish patterns of endangerment from patterns of language diversity. If an area has many languages, many will have smaller population sizes[12], and therefore may be more likely to be categorized as endangered. Secondly, competition with other languages has been cited as an important factor in language vulnerability, as language loss often occurs by the speaker population shifting to using another language[24,36-39]. We have several ways of describing the language diversity around each language.

We use polygon data to infer how many other languages are in contact with each language by counting the number of other language polygons that overlap, touch, or nearly touch each language's polygon(s) (defined as within 100 meters to account for slight polygon misalignment). We also express this as the number of contacting languages divided by the

perimeter of the language polygon. To reflect whether the languages in contact are all similar in size or whether there are some larger neighbouring languages alongside many smaller ones, we calculate the evenness of the target language and all languages in contact with it, based on their reported L1 population sizes.

We also calculated language diversity metrics for each language's neighbourhood (10,000 km^2 circle centred at the language's range polygon centroid). Values for an individual language were calculated by counting the number of intersections between a language's neighbourhood circle and other languages' range polygons. For example, the neighbourhood language diversity variable was just the count of number of intersecting languages, and the neighbourhood language evenness variable was calculated using the L1 speaker population values of each intersecting language using the evenness diversity metric

Evenness in L1 speaker population sizes was calculated using Pielou's evenness metric, calculated as $\frac{H'}{H'_{max}}$, where H' is the Shannon-Wiener entropy measure: defined as $H' = -\sum_{i=1}^{R} p_i \ln p_i$, where p_i is the proportion of the sum of all L1 speaker sizes being compared accounted for by the focal language i, and R is the total number of languages. This is a weighted measure of diversity which includes information from both the number of languages, as well as their relative amounts. To remove the effect of the number of languages, leaving only information about relative numbers (in other words the "evenness" of the L1 populations), the Shannon-Wiener index is divided by its theoretical maximum for the observed number of languages. This maximum is $H'_{max} = -\sum_{i=1}^{S} \frac{1}{S} \ln \frac{1}{S} = \ln S$, where S is the number of languages. This evenness metric was calculated using the *asbio* package in R[40].

All spatial intersections described above were done by first converting each dataset to a common cylindrical equal-area projection (EPSG:6933), before calculating their intersection using the *sf* package in R[14]. All other spatial operations mentioned in these methods likewise were conducted in this projection, except where it was possible to use spherical geometry operations (e.g. calculation of areas, perimeters and lengths).

2.3 Education

As a national-level indicator of average level of educational attainment, we include the mean years of schooling. We use the 2010 figures from the Barro-Lee Educational Attainment

Dataset[41], but for countries for which this is not available we use the median value from the United Nations Human Development Report (UNHDR) database[42] for 2005-2015 (Supplementary Table 4).

We also included data on government education spending as a percentage of GDP as an indicator of national policy and political attitudes toward education. Since World Bank and UNHDR data were provided in the form of time series, taking a ten-year median calculated from 2005-2015, the most data-rich period. Values for some territories were filled from regional aggregates, administering authorities or other sources (Supplementary Table 4).

General indices of educational activity do not account for the fact that different educational policies or attitudes may affect indigenous minority languages either positively or negatively. To address this, we gathered data on legal provision for the use of minority languages in education across different countries (Supplementary Table 5). We obtained the texts of linguistic laws and constitutional provisions for each country from a 20-year collection of legal texts (*L'aménagement linguistique dans le monde* [36]). For each territory, we coded the presence (1) or absence (0) of a general provision for the use of minority languages as media of instruction in at least a portion of formal or compulsory schooling.

We also recorded instances where provision was given to specific languages at regional or national levels (Supplementary Data 1). These data were converted to a language-level variable by scoring each language as positive (1) if it is a language specifically known to be used in education, as derived from the *L'aménagement linguistique dans le monde* database, or if it is the official or *de facto* official language of any territory as determined in Supplementary Table 3 and no other language is legally specified as the language of education for that territory. Languages were scored zero (0) otherwise.

These variables should be interpreted as broad-brush measures of education and support for minority languages and may not necessarily be an accurate reflection of the circumstances of any specific language or speaker population. Because information on education levels and policies is recorded at the national level, it may not be an accurate reflection of educational access for all populations and language groups in the country. However, we feel the general educational context is of sufficient importance in an analysis of language endangerment and loss that an imperfect indicator is better than none. For all variables recorded at national

level, if a language distribution spanned national borders, we took a weighted average value over the language's polygon/s.

2.4 Socioeconomic indicators

We use national Gross Domestic Product *per capita* (GDPpc) as an indicator of overall economic development (Supplementary Table 6). We also included the Gini index of income or consumption inequality within each country, from the Standardised World Income Inequality Database (SWIID), taking a ten-year median was calculated from 2005-2015[43].

We use life expectancy at 60 years of age (LE60) as a widely comparable indicator of average population health[44]. We use life expectancy at 60 years of age rather than life expectancy at birth, because health outcomes for community elders may play an especially important role in maintaining language continuity, and because life expectancy at birth is highly correlated with life expectancy at 60 (correlation coefficient = 0.87). To convert the gender-specific values of LE60 to a single value for each population, we used the proportion of males and females aged 60-64 to combine the life expectancy variables to a gender weighted average (see Supplementary Table 6).

These three socioeconomic indicators are predominantly only available at country-level. While socioeconomic data is available at a finer level of resolution for some countries, such states within the USA, we currently lack comparable data to allow a global analysis at sub-national level. However, we note that regions where countries have smaller geographic extent and less within-country variation in socioeconomic variables, for example in Oceania, do not detect associations between socioeconomic variables and language endangerment, even though they offer a finer scale and more exact measure of variation in GDPpc, LE60 and Gini than regions which are dominated by large countries with a wide geographic extent. Furthermore, the Gini Index, which describes economic inequality, will provide some reflection of the variation in socioeconomic status within each country.

2.5 Land use

To represent the both the socioeconomic landscape and the influence of the human modified environment on language vitality, we record the relative proportions of different land use types within the language polygon, specifically built environment, cropland, and pasture (Table 1). Proportion of land area of different types was calculated per grid-cell, where the grid-cell resolution was 5km squared, and then averaged over the polygon. We also use an

aggregate measure of the "human footprint" which attempts to reflect the extent to which the natural environment has been modified using a weighted combination of measures capturing built environments, crop land, pasture land, human population density, night-time lights, railways, roads, and navigable waterways (see Venter et al 2016 for details of weighting scheme). All of these land use measurements are 2009 values from the Human FootPrint (HFP) database, which were derived originally from satellite data[45].

Because of the available scale of the human footprint data, some smaller language polygons did not overlap any non-missing grid-cells in the HFP data. This was most commonly a problem for oceanic islands or small coastal languages, because there are many missing HFP values in oceanic areas. To correct this misalignment, for these very small language polygons only we used the neighbourhood, defined above: a 10,000 km^2 circle centred at the languages polygon centroid to collect the land use data instead of their original polygon. This resulted in those languages having better overlap with the Human Footprint dataset (282 languages in the final data were affected).

We also include a country-level measure of change in urban population from 1960 to 2017 as a measure of socioeconomic shift from rural to urban populations. This is calculated from World Bank data for the percentage of population in urban areas for each country[44], using the percentage point difference between the 2017 and 1960 values (Supplementary Table 6).

2.6 Environment

It is important to include environmental variables in a global analysis of language endangerment for several reasons. Firstly, environmental variables, particularly mean growing season and temperature seasonality, have been shown to be significant predictors of language diversity[12], so including them helps to control for the confounding effect of language diversity on endangerment patterns. Secondly, the association between biodiversity and language diversity can be accounted for by including environmental variables, so once we have basic features of climate in the model we do not need to include species richness[12]. Thirdly, there can be an intimate connection between language and environment[46], and language loss is likely to result in loss of specialist knowledge about natural environments[47,48], so we should include variables that represent climate and landscape as part of our explanation of language diversity and endangerment. Fourthly, the environment is undergoing rapid change due to human-induced climate change, so it is essential that we identify any associations between climate variables and language

endangerment so that we can predict possible future pressures on language vitality brought about by climate change[49].

We record a number of general environmental parameters for each language, averaged across all grids falling within the language polygon/s. Human population density is taken from the most recent values Gridded Population of the World (GPW) dataset, version 4 from the Socioeconomic Data and Applications Center[50]. We calculate the mean population density of grid-cells within language's polygon, with a grid-cell resolution of 1/24th degree (Long-Lat). We include a number of climate-based variables that have previously been shown to be the best environmental predictors of language diversity[12]. Mean growing season[51] is calculated as the number of days per year suitable for growing crops based on precipitation, evapotranspiration and soil moisture holding capacity from the Global Agro-ecological Zones (GAEZ) Data Portal version v3.0[52]. We also include mean average temperature, temperature seasonality and precipitation seasonality from the Worldclim global climate data set v2[53].

2.7 Biodiversity loss

We do not include measures of species diversity in this analysis, because climatic factors such as mean growing season and temperature and precipitation seasonality have been shown to provide significantly greater predictive power for language diversity than species richness[12]. However, the association between species extinction risk and language vulnerability has been a topic of interest for many researchers[54-58]. As an indicator of stress on the natural environment, we use the number of threatened vertebrate species within the language area. We use mammals and amphibians as data-rich indicators of biodiversity loss, counting the number of mammal and amphibian species that are rated Vulnerable or above in the IUCN[59], whose distribution overlaps the language polygon, based on a grid representation resolved to 100 km^2. To control for the influence of spatial variation in species diversity and for incomplete documentation of species endangerment we also express the number of threatened species (rated Vulnerable and above) as a proportion of all mammal or amphibian species (given the species included in the IUCN database, excluding Data Deficient species from the total).

2.8 Connectivity

Language contact has been recognized as a major source of language shift and endangerment, and relative isolation is often considered to reduce threats to language

vitality[37,39]. This is a very different situation from biodiversity where habitat connectivity is typically considered to reduce extinction risk[60] and small isolated populations are often considered the most vulnerable[61]. We have two broad ways of representing the degree to which humans can move freely over the landscape: paths of movement (roads and waterways) and barriers to movement (roughness and altitudinal range). All connectivity variables are calculated for the "neighbourhood" represented by a 10,000 km^2 circle centred on the language's range polygon centroid, as described above. This is because we wish to know how easily speakers of other languages can come into contact with each language, so we wish to characterise the ease of movement between each language and its surrounding areas.

Firstly, we represent the connection between people in the speaker population and other areas by representing the paths of movement offered by roads and waterways with the neighbourhood of each language. Average Road Distance is scored within a 5km squared grid-cell, based on a proximity raster (this includes distance to roads that are outside the grid cell). Road Distance Score was 1 for areas with an average distance of less than 0.5 km of a major road, and then decaying exponentially from 0.5 to 0 for areas with an average distance to a road of between 0.5 and 15 km away. We averaged the score across all grid-cells in the neighbourhood of each language[45]. Similarly, we average Navigable Waterway Distance across all 5km^2 grid-cells within the language neighbourhood, where areas directly adjacent to a Navigable Waterway were given a score of 1, which then exponentially decays to zero at 15 km away (for the definition of a Navigable Waterway, see Venter et al. 2016[45]).

Secondly, we represent the ease of human movement as a function of the altitudinal range and average landscape roughness within the language neighbourhood. Roughness is represented by the autocorrelation in altitude calculated at every 1km along 100 km length transects, averaged over eight different directions from the SRTM30 elevation[62]. Average altitudinal range across all grid-cells within the language neighbourhood, with a grid-cell resolution of 5km^2. Altitudinal range is defined simply as the difference between the maximum and minimum altitude within a grid-cell[63].

2.9 Shift

We have a number of variables to represent the degree to which human impact on environment is changing within each language's area, which we refer to as "Shift" variables.

These shift variables are expressed as the average change per year over the available time period. Change in human population density per year since the year 2000 is based on data from the Gridded Population of the World (GPW) dataset, version 4[50]. We calculate the average rate of change in land use per year, averaged over the language's polygons from data provided in the Human Footprint database[45], including cropland (from 1992 to 2005), pasture (from 1993 to 2009), and built environment (from 1994 to 2009). We also use the rate of change per year in the aggregate Human Footprint (see 2.5 above), calculated as the Human Footprint rating of a grid-cell in 2009 minus the Human Footprint rating for 1993, averaged over a language's polygons.

2.10. World languages

One of the commonly discussed threats to language vitality is the shift to using a wide-spread *lingua franca*, which may be the language of government, education and business, and may have greater prestige in some social settings[64,65]. In particular, there are a number of world languages, spread by conquest, colonialism and commerce, that overlay many Indigenous languages. We use the term "world language" to describe languages with a very large numbers of speakers, in particular many L2 speakers, that have spread far beyond their original homelands (Supplementary Table 2). Nine world languages are included in this analysis as variables, rather than as languages to be analysed, in order to test the hypothesis that world languages can represent "killer" languages[66-70], leading to replacement of autochthonous languages either by force (such as requiring children to speak a colonial language instead of their home language at school), by prestige (when viewed as a tool of upward social mobility), by political structure (such as being the language of government) or by commercial pressure (being the language of business or a common *lingua franca* in trade). However, not all researchers are convinced that world languages should be regarded as "killers" of linguistic diversity[71]. Therefore we think it valuable to ask whether co-existing with a world language is consistently associated with increased levels of language endangerment.

Given this broad definition of "world language", we needed to identify which languages to remove from the analysis and treat as variables that might influence language vitality, and we did this on the basis of numbers of L1 and L2 speakers and geographic spread. Any such list must represent a somewhat arbitrary cut-off, and not all colonial languages or *lingua francas* are included in our list of world languages. For example, although Dutch [nld] and German [deu] are colonial languages that have official recognition in countries far from

their original homeland, they are not included here because they have less than half the number of speakers than any of the other nine world languages (German 100 million speakers with 10% L2; Dutch: 24 million L1 and 5 million L2 speakers, but if including Afrikaans [afr] then 46 million total). In some countries, the spread of a Creole as a *lingua franca* is considered a major threatening process for language vitality, for example shift to Tok Pisin [tpi] in Papua New Guinea, has been identified as a threatening process for many Papuan languages[72,73]. But we have not included them as "world languages" because they do not have the same geographic reach or speaker population size as other world languages. While these languages may be important factors in local patterns of language shift, this analysis is specifically concerned with global determinants of endangerment status, and will necessarily not be able to include threatening processes that are specific to particular countries or regions. For the same reason, we have not included cases of local languages expanding within their own region and overlaying or replacing neighbouring languages, such as Quechua [que] and Swahili [swa][74,75]. Expansion of local languages will be critical factors in investigating local impacts on language diversity within particular regions[76], but less useful for identifying factors that have a global impact.

For each language in our dataset, we ask if it occurs in a country that has one of the nine world languages (Supplementary Table 2) as an official language (Supplementary Table 3). Where a language overlaps more than one country, we use a weighted average according to the degree of coverage of the language polygons over different national regions (note that this means that languages that have any degree of inferred distribution in a country with a world language will have a non-zero value for world language, even if they are predominantly found in a country without a world language as official language). In some countries, a world language acts as the main language of commerce, education and trade even if it doesn't have official status. For example, neither the United States of America nor Australia have any nationally recognized official languages, but the exclusive use of English in almost all educational settings is likely to be an important factor in Indigenous language endangerment and loss in both countries[77-80], so we count English as a world language for these countries even though it does not have official recognition. In order to allow for the fact that different world languages could have different effects on language vitality of co-existing languages, we include nine variables for the presence or absence of each of the nine listed world languages. We also include a variable that records the co-existence of a language with any (or several) of the nine world languages. This variable is the weighted

average of world languages that overlap with a language polygons (which has low correlation with the binary variable for each world language).

3. Hierarchy of relatedness

We need to account for similarity between languages due to the evolutionary relationships between languages. Related languages will tend to be more similar in many aspects, not just in language structure, lexicon, grammar, phonology and so on, but also with respect to their shared environments, shared histories and common socioeconomic factors. For example, we expect two languages from the Pama-Nyungan family in Australia to be more similar to each other in many respects, including climate, political history, and educational environment, than either is to two languages from the Dravidian language family. Many language specific variables, such as population size or area, might also be more similar between close relatives. Failure to account for non-independence due to patterns of relatedness leads to pseudoreplication (counting copies of the same occurrence as multiple instances of independent occurrences), inflating apparent relationships between variables[81,82]. We can use information on relatedness to capture these patterns of covariation in the data in order to look for significant associations between variables above and beyond those due to shared inheritance.

While some language families have published phylogenetic trees, for a global analysis we need to be able to estimate the relative degree of relatedness among all languages. There is currently no widely accepted global language phylogeny, as the relatively rapid rate of language change presents a challenge to resolving deep history, such that relationships between language families are controversial[83,84]. While there have been attempts to estimate a global language phylogeny[85,86] these remain controversial, particularly concerning the deeper branches in that tree (e.g. clustering Japanese and American languages, or grouping the Kiowa-Tanoan languages in North America with Dravidian languages in India)[87,88]. These issues highlight how challenging quantitative estimation of deep language phylogeny is. For this reason, we take the conservative approach of using a taxonomy that is based on a wide range of language characteristics in order to approximate degree of relatedness between languages. Taxonomy provides a useful proxy because it groups languages according to their likely relationships[88,89]. A taxonomy is not a phylogeny, because it does not aim to represent the history of diversification of languages over time, but it provides exactly the kind of information we need to adjust our analysis for the covariation between languages, because taxonomic groups summarise the similarities due to relationships.

However, we also need information on the relative expected similarity of related groups of languages. This is reflected in "branch length" of phylogenetic diagrams, on the grounds that two languages that have been evolving separately for a long period of time are likely to be more dissimilar to each other than a pair of recently separated languages.

We use the taxonomy in Glottolog to determine a nested hierarchy of language relationships, and the method in Bromham et al.[81] to scale branch lengths. In brief, the method assumes that all the language families belong to the same taxonomic level with node height 1 and all extant languages (i.e. the 6511 languages in our database) have height 0. The node height of a taxonomic group within a language family is set to the estimated age of the group relative to the age of the language family if a dated phylogeny of the family is available and the node of the group is identifiable in the phylogeny. We extract node estimated ages from all published dated phylogenies of various language families as summarized in D-PLACE[90]. D-PLACE has matched tip names of each phylogeny to glottocodes. We further match nodes in each phylogeny to taxonomic groups in Glottolog. A node is matched to a taxonomic group if all the languages descending from that node belong to the same taxonomic group and if at least one representative language of each subgroups in the taxonomic group is included in the phylogeny.

For nodes which do not have a node age provided in D-PLACE, we assign approximate node heights to each node, using the following procedure, starting from the node closest to root, and moving toward the tips. Since each node may have multiple descendant nodes with known height, we set the branch length between the node and its nearest ancestor to the minimum of the difference in node height between the nearest ancestor and any of the descendant nodes with known height divided by the number of edges connecting them. This step allows us to evenly distribute any nodes of unknown height between any two nodes with known age without causing conflicts in node height if the node connects more than one pair of end nodes with known heights.

4. Statistical analysis

4.1 Predictor Transformations

In order to reduce the possibility of high skew in predictor variables creating large leverage for a small number of data points, we transformed our predictor variables to make them more symmetric and to reduce long tails on the distribution of values. Because of the large

number of variables, we used an automated method to choose a suitable transformation. The validity of chosen transformations was checked by visualising the distribution of the variable after transformation (e.g. a histogram), and checking for reasonable symmetry and lack of skewness. We also plotted the transformed predictors with the response variable to check for linearity.

For the automated method, we chose from Tukey's "Ladder of Powers" transformations[91] by first fitting a Box-Cox power transformation (x^{λ}, except where lambda $\lambda = 0$, where it is $\log(x)$) to each variable and maximizing its fit to a standard Normal distribution[92]. We then "snapped" this continuous estimate of lambda to its nearest value corresponding to a standard power transformation (e.g -2, -1, -0.5, 0, 0.5, 1, 2, 3). These values of lambda for Box-Cox correspond to simple transformations on Tukey's ladder. This produces a more standardised and interpretable transformation than using the continuous Box-Cox, which can generate transformations that are intermediate between the standard power transformations. The standard power transformations used in this study are: negative reciprocal ($-\frac{1}{x}$), log ($\log(x)$), square root ($\sqrt{x}$), identity (x), square (x^2), and cube (x^3). For variables that were log transformed we also added a small constant before logging to reduce artifacts caused by values equal to or very close to zero. The constant added was calculated as: $\frac{1}{2}\min(x)$, which was found to produce consistently symmetrical distributions.

The only exception to these standard power transformations was for the Shift variables (see Section 2.9 and Table 1), which ranged naturally between negative and positive values of change, but which tended to have very long tails (often in both directions). To reduce the extreme values, we transformed all Shift variables using a signed square root transformation ($\mathrm{sgn}(x)\sqrt{x}$). The transformation used for each variable can be found in Table 1.

4.2 Regression model

The dependent variable in our analysis is current language endangerment status (Supplementary Table 1) which is an ordinal variable, so we use an autoregressive ordinal probit regression to model language endangerment status[93]. The regression model assumes a latent continuous dependent variable that follows a normal distribution and a set of cut points that divide the latent variable into bins to match levels of threat status. An independent variable increases or reduces endangerment status by increasing or decreasing

the mean of the latent variable. This implies the parallel regression assumption that the regression coefficients of each independent variable are the same across levels of endangerment status, such that there is no change in the nature of the relationship between an independent variable and endangerment over the different levels of language endangerment (Supplementary Data 1). To ensure that any identified relationships between predictor variables and endangerment are continuous across all levels of endangerment, not restricted to particular levels of endangerment, we apply a test of parallel assumption of the model. We show that the effects of various variables on shifting the endangerment level of a language are consistent across levels, including the highest level of endangerment (Sleeping languages that are no longer spoken).

To test the parallel regression assumption, we apply likelihood ratio test for each predictor to evaluate whether fitting different regression coefficients to different levels of endangerment level significantly increase model fit, using the "ordinalNet" R package[94]. When applied to the 13 levels of the EGIDS scale (Table 1), three quarters of predictors violate the parallel regression assumption and have opposite effects on levels 1 up to 6a and from levels 6a up to 10 (Supplementary Data 2). In other words, this analysis suggests many of the predictor variables has a different form of relationship for languages that are not currently endangered (EGIDS levels 1-6a) than for those languages that endangered (EGIDS level 6b-10). Since languages rated at levels 1 to 6a in the EGIDS scale are considered to be actively used, learned by children and not currently losing speakers, we group levels 1 to 6a to a single level - "Stable" – and retain the divisions from 6b to 10 (Supplementary Table 1). This grouping results in 7 levels, which satisfies the parallel regression assumption for the majority of the predictors (Supplementary Data 2). All the numeric predictors are centred (subtracted from the mean) and scaled (divided by standard deviation) before analyses, so that the magnitude of their regression coefficients reflects their relative impact on language threat status.

The regression model accounts for autocorrelation among languages by including an endogenous variable in the model, which is a matrix-vector product, where the vector is the endangerment status of each language and the matrix describes the degree of autocorrelation between two languages. So the endogenous variable measures the effect on a language's endangerment status due to its autocorrelation with all the other languages. This autocorrelation matrix is constructed as a weighted sum of a phylogenetic matrix to account for autocorrelation due to relatedness, a distance matrix to account for

autocorrelation due to spatial distance, and a contact matrix to account for autocorrelation due to contact between languages. The phylogenetic matrix is constructed from the phylogenetic correlation matrix of the language hierarchy (see 3). The distance matrix is calculated from the exponential of the negative great-circle distance between the centroids of polygons of two languages (see 2.1.3), which accounts for lower autocorrelation over larger distances. The contact matrix is calculated from a matrix containing 1 if two languages overlap in polygons (see 2.2), and 0 otherwise. All the three matrices are standardised to have 0 diagonals and row sums equal to 1. The weights of these matrices are estimated by maximising the likelihood of a regression model with intercepts given the EGIDS scores of all the languages, using the "L-BFGS-B" method[95] in the "optim" function in R.

4.3 Candidate models

Given the large number of predictors, we first group similar predictors together in order to increase the efficiency of the model selection procedure. Using the full dataset, we estimate the correlation coefficients between all variables (Supplementary Figure 4). We then group together those variables that have an absolute correlation coefficient with each other higher than 0.5, so that predictors from different groups have relatively weak correlation (groups of variables are indicated in Supplementary Figure 4 and Supplementary Data 3 by lines separating the grouped variables). Because of the high correlation within groups but low correlation between groups, adding or removing a variable of one particular group to or from the model has little influence on the predictive power of variables in other groups. Therefore we can update the model by only testing variables in the same group at a time (as explained in more detail below). All groups are visited in a specific order to complete one iteration of model selection. To account for the weak correlation between groups, we randomize the order of the groups in each iteration. Iterations stop when there are no more variables added or removed from the model.

In detail, the algorithm starts with finding a model with only one predictor variable that provides the best fit to the training data set (i.e. two thirds of the languages), where model fit is measured by the likelihood value. Then a random order of groups is generated. For each group in the order, each variable in the group is added to the model separately. Within that group of covarying variables, we select the variable that gives the greatest (and statistically significant) increase in model fit to keep in the model. Then we remove each of the remaining variables in the group separately. We select the variable whose removal

results in the smallest (and statistically non-significant) to be removed from the model. We apply this procedure of adding the variable that has the largest impact on the fit of the model and dropping the variable that has the least impact on the fit of the model for every group of co-varying variables. Once all the groups have been visited this way in turn, a new random order of groups is generated and the procedure starts again from the first group in the new order. This continues until there are no more variables that we could add to significantly increase the fit, and there are no more variables that we could remove without significantly decreasing the model fit. We repeated the algorithm 50 times on the same training dataset with different random seeds.

For each of the models selected by the above procedure, we use lasso to reduce overfitting, with equal weight to two penalties, referred to as L1 norm (which penalizes large regression coefficients) and squared L2 norm (penalizes large difference in regression coefficients between highly correlated independent variables). Note that here L1 and L2 are not used in the linguistic sense of mother tongue and second language speakers but as statistical terms associated with this model fitting procedure[96]. We use this procedure to generate a total of 100 candidate models, 50 candidate models without lasso and 50 candidate models with lasso (Supplementary Data 3), which we then compare using the following model selection procedure

4.4 Model selection

To measure the predictive power of each candidate model, we first apply 10-fold cross-validation to each model using the training dataset, in which the dataset is randomly divided into 10 groups (or folds). This gives us, for each model, the mean and the standard error in percentage deviance explained (the amount of variation in endangerment levels among languages that is predicted by the model), in Brier scores (the mean squared error in the predicted probability and the actual probability of a language having an endangerment level), and in misclassification rates (how often a language is predicted to be in a different endangerment level than its current EGIDS score). These three statistics are common measures for accuracy of predictions[94]. We then use each model to predict endangerment level of the languages in the test dataset and measure accuracy of predictions by the same three measures. Comparing these measures of the test dataset to their standard errors as estimated from the training dataset allows us to test whether two models have significantly different predictive power, which is essentially a *t*-test.

We reject any of the 100 candidate models that do not meet our selection criteria. To be retained, the model must satisfy the following conditions:

(1) the model does not differ significantly in percentage deviance explained from the model with the largest percentage deviance explained;

(2) the model does not differ significantly in Brier score from the model with the lowest Brier score;

(3) the model does not differ significantly in misclassification rate from the model with the lowest misclassification rate.

Finally, we identify variables that are included in more than one third of the retained models, use these variables to construct the best model, and fit the best model to data from all the languages. The fitted best model is used to make future prediction of all the languages. Results of model selection are in Supplementary Data 3.

4.5 Model outcomes

Comparing the predictions of the best model to the observed data (Supplementary Data 1), we find that, the model is generally able to correctly identify Stable languages (EGIDS 1-6a) and the "extinct" level (EGIDS 10), while tends to underestimate the other levels of endangerment in between (Supplementary Figure 4). Languages with underestimated endangerment levels are mainly distributed in one hotspot of endangered languages, in the surroundings of Himalayas (Extended Data Fig. 3), suggesting that the model fails to include the main predictor of language endangerment levels in this area. There are exceptions where the model predicts higher levels of endangerment than is recorded in the database, for example, West Australia, potentially due to the low L1 population sizes of many of these languages (see Supplementary Data 1). Areas of higher predicted than observed endangerment may indicate "latent risk", areas where we might expect an increase in language endangerment in future[97] (Extended Data Fig. 1).

5. Future prediction

Current patterns of endangered languages are given in Figure 2. Our model selection procedure gives us a set of variables that have predictive value on level of language endangerment (Supplementary Figure 7). We can combine this model with information about endangered languages to project likely patterns of loss of language diversity in the future. To project future patterns of language endangerment, we first model shift in

endangerment status due to generational shift (the ageing of current L1 speakers): these results are presented in the main manuscript (Figures 2 & 3).

Our model identifies predictors of language endangerment that are likely to change over time, such as land-use and climate, so we can also use the model to predict likely patterns of endangerment under future values of these variables. Here we demonstrate this approach using future values of temperature seasonality (which is a significant correlate of language change in some regions) and human impact on the landscape (represented by "Shift" variables: Table 1, Supplementary Figure 3). These variables are not globally significant predictors of language endangerment, but are associated with patterns of endangerment in some regions: they will only influence predicted patterns of language endangerment in regions in which they are identified as significant predictors in the model. We first adjust endangerment according to demographic predictions, then we use our model to further adjust endangerment level based on projected future values of key predictors.

We predict endangerment status at two future time points, +40 years (the year 2060) and +80 years (2100). These intervals are determined by available climate change predictions and as appropriate time steps for characterising generational shift.

5.1. Generational shift

Some of the EGIDS language threat categories give us clear predictions about future status, due to generational change in the speaker population (Supplementary Table 1). If a language is not being learned by children, then without intervention to restore language transmission, there will be no L1 speakers after the current speakers have died. Therefore we can use current EGIDS score to predict future level of endangerment (Supplementary Table 7). Languages rated at EGIDS levels 9 or 10 have no active L1 speakers and are not currently being learned by children, so without intervention and revitalisation, these languages will be rated Sleeping (no L1 speakers) at all future time points. Critically Endangered languages (EGIDS 8a and 8b) are currently spoken only by elders, so for these languages, we assume that without language revitalisation, all current L1 speakers will be dead within 40 years and therefore the language will be Sleeping (no L1 speakers) in both 2060 and 2100. Endangered languages (EGIDS 7) have adult L1 speakers but are not being learned by children, so without intervention, once these adult speakers have died, there will be no more L1 speakers, therefore we expect that in 40 years only elderly speakers will

remain and so the language status will change to 8 in 2060, and in 80 years no L1 speakers will be left alive and the status will change to 10 (Sleeping).

For languages that are still being learned by children, the future endangerment status cannot be so clearly forecast, but we wish to conservatively predict which of the languages currently rated 6b might become endangered in future. We cannot assume that small population size alone makes a language vulnerable, because some languages with small numbers of speakers are still being reliably transmitted across generations. So we make use of alternative language endangerment scales (Supplementary Table 1) to identify languages that may be subject to future erosion of generational transmission. Of the languages rated Threatened (6b) in Ethnologue, there are 468 rated Shifting in AES, 466 rated Threatened in AES, 62 rated Moribund and 8 rated Nearly Extinct (we discuss in Section 1 above why AES and EGIDS do not always agree and why we have chosen EGIDS for this analysis). We take an AES rating of Shifting, Threatened, Moribund or Nearly Extinct to indicate that a 6b language may have less stable generational transmission, and that this may result in increase in threat language in smaller populations which are the most vulnerable to attrition in first-language speakers. Therefore for languages rated 6b in EGIDS that have a current L1 speaker population of less than 1000 (which is the median population size for Shifting (7) languages), and for which AES rates the languages as Shifting, Threatened, Moribund or Nearly Extinct, we adjust the threat level from 6b to 7, in +40 years, and from 7 to 8 in +80 years. We do not adjust the endangerment level of any Stable languages (EGIDS 1-6a) at this step.

5.2. Model predictions

Language erosion due to generational shift is inexorable: without intervention and revitalisation all languages currently rated 7 and above will have no remaining L1 speakers in the next century. Therefore the adjustment to endangerment level based on generational shift, described in 5.1, is the baseline for our prediction of language endangerment and loss. But we can also use our model predictors to ask if change in environmental conditions will place even greater threat on languages, in addition to the loss of L1 speakers through generational shift. Our model identifies environmental, socioeconomic and demographic variables that are significantly associated with language threat level above and beyond the variation explained by covariation due to relatedness, spatial proximity or attributable to covariation among explanatory variables. Given that some of these predictors are variables that will change over time, we wish to use the best model to make predictions about

languages that are likely to come under increasing threat in future. Some of the variables in our model will likely remain unchanged, such as island status, and for other variables we have no way of reliably predicting future values, such as spending on education. But there are some variables where we can predict future values given the information we have. These fall into three categories: demographic, environmental and climate.

5.2.1 Demographic shift in L1 speaker population

Languages rated 6b and above are still being learned by children, but are considered to be losing speakers, so we adjust the L1 speaker population size at future time points to reflect this predicted loss. Any language currently rated 8, 9 or 10 will have a L1 speaker population of zero at +40 years and +80 years. Languages rated 7 currently have only adult speakers, with no new L1 learners. If the majority of current adult L1 speakers are over 25, then in 40 years, only speakers older than 65 will remain. To estimate what proportion of the current L1 speakers will be alive in 40 years, we use World Bank regional estimates of the percentage of the population over 65 (Supplementary Table 8). For example, for a sub-Saharan language rated 7, we would assume only 3% of the current L1 speakers will be alive in 40 years, but 15.9% of speakers of an Australian language rated 7 might still be alive in 40 years. Clearly this is a very rough approximation, as it ignores the age structure of the current L1 speaker population (making the conservative assumption that most of the L1 speakers of the language at the present time are younger adults rather than elders) and it does not account for the possible differences in age structure and life expectancy across regions (for example, Indigenous populations in Australia have a life expectancy that is 10 years lower than the national average[98]).

To model the potential loss of speakers over time we need a rule-of-thumb for how rate of decline in L1 speakers in an endangered language. Therefore, we reduce the speaker population of a language rated 6b by 30% in 40 years (equivalent to 0.75% per year). This value is arbitrary, as it would be impossible to estimate an accurate rate of decline for all languages, but it is compatible with an average rate of decline across a range of examples for which we have access to surveys of decline in L1 speaker population over time. For example, in Europe, Welsh was documented as losing 0.5% of L1 speakers per year between 1901 and 1991[99], Slovenian is considered to have lost 0.8% L1 per year between 1881 and 1991[100], and Scottish Gaelic 0.6% per year from 1881 to 1981[101]. It is important to emphasize that we are concerned with shift in first-language acquisition (i.e. L1 speaker population), not the size of minority ethnic populations or the total number of speakers. For

example, the Australian language Bardi is recorded as losing 1% of L1 speakers per year between 1900 and 2011, although the population of Bardi people was growing over this time[102]. In North America, census data suggests a rate of decline in speakers of the Navajo language (Diné Bizaad) of 0.8% per year from 1980 to 2000, but there is growing educational resources that promote learning of Navajo which may result in an increase in speakers. It is surprisingly difficult to find good data on rates of loss of L1 speakers over appropriate time spans. This small sample is not intended as a comprehensive characterisation of language decline rates, but it does provide a degree of empirical anchoring for the decline rate, which is consistent with more general trends reported (e.g. decline in people speaking Indigenous Australian languages at home[103]). Since these examples show a roughly linear decline in L1 population size over years, we calculated their average rate of decline over 40 years in L1 population size as the difference between the earliest and latest records of L1 population size divided by the number of years in between, and multiplied by 40 years.

5.2.2 Climate change

We recognize that many aspects of climate change may impact on language vitality in future, such as the effect of sea-level change on the area of oceanic islands, or through influencing the agricultural productivity of particular regions or the reliability of staple crops. But here we are specifically modelling only those factors that are identified in our model as significant predictors of current patterns of language endangerment. Our models identify temperature seasonality is a consistent significant predictor of language threat in Europe, and Central and East Asia (Supplementary Data 3, Supplementary Figure 7). As global temperatures rise temperature seasonality is also expected to change over time. We model the change in language threat expected to result from projected changes in temperature seasonality up to 2060 and 2100, using the climate projection models available in WorldClim (worldclim.com). Climate projections in WorldClim are based on several different Global Circulation Models and emissions intensities (Shared Socioeconomic Pathways)[53]. We chose the CNRM-ESM2-1 model and ssp370 as a realistic mid-range scenario for projected temperature seasonality. Gridded temperature seasonality data for 2041-2060 and 2081-2100 were downloaded at 2.5 minute resolution, and values were averaged for language polygons as described in Supplementary methods Section 2.

5.2.3 Environmental change in land use and built environment

Aspects of the human-modified environment are significant predictors of language endangerment at both the global level (e.g. road density) and regional level (e.g. built environment in South East Asia, pasture and cropland in Africa). Since human modification of landscape is ongoing in many parts of the world, we wish to predict future values of these land use variables in order to evaluate possible effects on future language vitality. We calculate average rates of change per year from our Shift variables (see section 2.9), including change in population density between 2000 and 2020, change in human footprint between 1993 and 2009, change in croplands over 1992 and 2005, change in built environment over 1994 and 2009, change in pasture over 1993 and 2009, change in croplands (from 1992 to 2005) (see Table 1). We model a linear change in population density, human footprint, croplands, built environment, and pasture, assuming a constant rate of change per year calculated from their corresponding shift variables, so the projected values for these variables in each grid cell in 2060 and 2100 are their rate of change per year within that grid cell multiplied by 40 and 80 years. Clearly a linear rate of change may be an oversimplistic project, but we apply it here as a proof-of-concept exercise for incorporating land-use change in future prediction of language endangerment patterns. Alternative models currently have relatively wide confidence intervals on future predictions, but could be substituted for a linear projection if robust predictions of relevant land-use variables were developed[104,105].

6. Supplementary references

1 Lewis, M. P., Simons, G. F. & Fennig, C. D. Vol. Online version: http://www.ethnologue.com. (SIL International, Dallas, Texas:, 2013).

2 Lewis, M. P. & Simons, G. F. Assessing endangerment: expanding Fishman's GIDS. *Revue roumaine de linguistique. 2010 Apr 1;55(2):* **55**, 103-120. (2010).

3 Grenoble, L. A. & Whaley, L. J. Toward a typology of language endangerment. *Endangered languages*, 22-54 (1998).

4 Moseley, C. Atlas of the World's Languages in Danger (UNESCO Publishing, Paris, 2010).

5 University of Hawaii at Manoa. (2019).

6 Lee, N. H. & Van Way, J. Assessing levels of endangerment in the Catalogue of Endangered Languages (ELCat) using the Language Endangerment Index (LEI). *Language in Society* **45**, 271 (2016).

7 Lee, N. H. & Van Way, J. The Language Endangerment Index (LEI). in *Cataloguing the world's endangered languages* (eds L. Campbell & A. Belew) (Routledge, 2018).

8 Hammarström, H., Forkel, R. & Haspelmath, M. (Max Planck Institute for the Science of Human History, Jena, 2019).

9 Hammarström, H. *et al.* Simultaneous visualization of language endangerment and language description. *Language Documentation & Conservation* **12**, 359-392 (2018).

10 Amano, T. *et al.* Global distribution and drivers of language extinction risk. *Proceedings of the Royal Society B: Biological Sciences* **281**, 20141574 (2014).

11 Sutherland, W. J. Parallel extinction risk and global distribution of languages and species. *Nature* **423**, 276-279 (2003).

12 Hua, X., Greenhill, S. J., Cardillo, M., Schneemann, H. & Bromham, L. The ecological drivers of variation in global language diversity. *Nature Communications* **10**, 2047, doi:10.1038/s41467-019-09842-2 (2019).

13 velox: Fast Raster Manipulation and Extraction, R package, version 0.2.0.9002. (2018).

14 Pebesma, E. Simple Features for R: Standardized Support for Spatial Vector Data. *The R Journal* **10**, 439-446, doi:https://doi.org/10.32614/RJ-2018-009 (2018).

15 Braithwaite, B. Sign language endangerment and linguistic diversity. *Language* **95**, e161-e187 (2019).

16 Crevels, M. in *The indigenous languages of South America: A comprehensive guide. Vol. 2. 2012* (eds L. Campbell & V. Grondona) 167-234 (Walter de Gruyter, 2012).

17 Benjamin, G. *A New Outline of Temiar Grammar. Part 1.*, (Nanyang Technological University, 2020).

18 Hinton, L. in *The Green book of language revitalization in practice* (eds L. Hinton & K. Hale) 413–417 (Brill, 2001).

19 Hobson, J. R. *Re-awakening languages: Theory and practice in the revitalisation of Australia's Indigenous languages.* (Sydney University Press, 2010).

20 Rehg, K. L. & Campbell, L. *The Oxford handbook of endangered languages.* (Oxford University Press, 2018).

21 Tershy, B. R., Shen, K.-W., Newton, K. M., Holmes, N. D. & Croll, D. A. The Importance of Islands for the Protection of Biological and Linguistic Diversity. *BioScience* **65**, 592-597, doi:10.1093/biosci/biv031 (2015).

22 Boas, F. *Handbook of American Indian Languages (Bulletin 40, Part 1, Bureau of American Ethnology),* . 1-79 (Washington: Government Printing Of- ce, 1-83. Reprinted 1966 [1991]. Lincoln: University of Nebraska Press., 1911).

23 Boas, F. Introduction. *International Journal of American Linguistics* **1**, 1-8 (1917).

24 Himmelmann, N. Documentary and descriptive linguistics. *Linguistics* **36**, 161-195 (1998).

25 Meakins, F., Green, J. & Turpin, M. *Understanding linguistic fieldwork.* (Routledge., 2018).

26 Terrill, A. Why make books for people who can't read? A perspective on documentation of an endangered language from Solomon Islands. *International Journal of the Sociology of Language* **155**, 205-219 (2002).

27 Amery, R. Phoenix or relic? Documentation of languages with revitalization in mind. *Language Documentation & Conservation* **3**, 138-148 (2009).

28 Austin, P. K. & Sallabank, J. *Endangered languages: Beliefs and ideologies in language documentation and revitalization.* (British Academy, 2014).

29 Woodward, J. Endangered Sign Languages in *The Oxford Handbook of Endangered Languages* 168 (Oxford University Press, 2018).

30 Nonaka, A. M. The forgotten endangered languages: Lessons on the importance of remembering from Thailand's Ban Khor Sign Language. *Language in Society* **33**, 737-767, doi:10.1017/S004740450404504X (2004).

31 Maypilama, E. & Adone, D. Yolngu sign language: An undocumented language of Arnhem Land. *Learning Communities: International Journal of Learning in Social contexts*, 37-44 (2013).

32 Kendon, A. *Sign languages of Aboriginal Australia: Cultural, semiotic and communicative perspectives.* (Cambridge University Press, 1988).

33 Woodward, J. *et al.* Ho Chi Minh City Sign Language in *Sign Languages of the World: A Comparative Handbook* (eds Jepsen Julie Bakken, Clerck Goedele De, Lutalo-Kiingi Sam, & B. McGregor William) 391-408 (De Gruyter Mouton, 2015).
34 Johnston, T. W (h) ither the Deaf community? Population, genetics, and the future of Australian Sign Language. *Sign Language Studies* **6**, 137-173 (2006).
35 Barnet, B., McDonald, R., Taffe, S. & Kaufman, J. Cochlear Implants and Sign Language in Australia: Why the Deaf Community Must Embrace Non-Signing Implant Recipientsin *Reimagining Communication: Action* 276-285 (Routledge, 2020).
36 Mufwene, S. S. *The ecology of language evolution.* (Cambridge University Press, 2001).
37 Romaine, S. in *The Handbook of language contact* (ed R. Hickey) 320-339 (Wiley-Blackwell., 2013).
38 Sallabank, J. & Austin, P. *The Cambridge handbook of endangered languages.*, (Cambridge University Press, 2011).
39 Thomason, S. G. in *The Oxford handbook of endangered languages* (eds Kenneth L Rehg & Lyle Campbell) 1-18 (Oxford University Press, 2018).
40 Aho, K. asbio: A Collection of Statistical Tools for Biologists. R package version *1.6-7.* **https://CRAN.R-project.org/package=asbio** (2020).
41 Barro, R. L. & Lee, J.-W. A new data set of educational attainment in the world, 1950-2010. . *Journal of Development Economics* **104**, 184-198 (2013).
42 United Nations Development Programme. Human Development Indices and Indicators: 2018 Statistical Update. http://hdr.undp.org/en/2018-update. . (2018).
43 Solt, F. The Standardized World Income Inequality Database, Version 8. https://doi.org/10.7910/DVN/LM4OWF. (2019).
44 World Bank. World Development Indicators. World Bank Publications. URL: http://data.worldbank.org/data-catalog/world-development-indicators (2019).
45 Venter, O. *et al.* Sixteen years of change in the global terrestrial human footprint and implications for biodiversity conservation. *Nature communications* **7**, 1-11 (2016).
46 Riseth, J. Å. *et al.* Sámi traditional ecological knowledge as a guide to science: snow, ice and reindeer pasture facing climate change. *The Polar Record* **47**, 202 (2011).
47 Cámara-Leret, R. & Bascompte, J. Language extinction triggers the loss of unique medicinal knowledge. *Proceedings of the National Academy of Sciences* **118** (2021).
48 Kik, A. *et al.* Language and ethnobiological skills decline precipitously in Papua New Guinea, the world's most linguistically diverse nation. *Proceedings of the National Academy of Sciences* **118** (2021).
49 Cochran, P. *et al.* in *Climate Change and Indigenous Peoples in the United States* 49-59 (Springer, 2013).
50 Center for International Earth Science Information Network (CIESIN). (ed NASA Socioeconomic Data and Applications Center (SEDAC)) (Columbia University, Palisades, NY, 2018).
51 Nettle, D. Explaining global patterns of language diversity. *Journal of anthropological archaeology* **17**, 354-374 (1998).
52 FAO & IIASA. Global Agro-ecological Zones (GAEZ v3.0) (eds (FAO). Food and Agriculture Organization of the United Nations & (IIASA). International Institute for Applied Systems Analysis) (Laxenburg, Austria, 2010).
53 Fick, S. E. & Hijmans, R. J. WorldClim 2: new 1-km spatial resolution climate surfaces for global land areas. *International Journal of Climatology* **37**, 4302-4315, doi:10.1002/joc.5086 (2017).
54 Loh, J. & Harmon, D. Biocultural diversity: threatened species, endangered languages. *WWF Netherlands, Zeist, The Netherlands* **1** (2014).
55 Harmon, D. & Loh, J. in *The Oxford handbook of endangered languages* (eds Kenneth L Rehg & Lyle Campbell) 1-32 (Oxford University Press, 2018).

56 Maffi, L. Linguistic, cultural, and biological diversity. *Annu. Rev. Anthropol.* **34**, 599-617 (2005).
57 Pretty, J. *et al.* The intersections of biological diversity and cultural diversity: towards integration. *Conservation and Society* **7**, 100-112 (2009).
58 Turvey, S. T. & Pettorelli, N. Spatial congruence in language and species richness but not threat in the world's top linguistic hotspot. *Proceedings of the Royal Society B: Biological Sciences* **281**, 20141644 (2014).
59 IUCN. International Union for the Conservation of Nature (IUCN) Red List Version 2019-2. http://www.iucnredlist.org (2019).
60 Schaffer-Smith, D., Swenson, J. J. & Boveda-Penalba, A. J. Rapid conservation assessment for endangered species using habitat connectivity models. *Environmental conservation* **43**, 221-230 (2016).
61 Soulé, M. Viable Populations for Conservation (Cambridge University Press, Cambridge, 1987).
62 Becker, J. *et al.* Global bathymetry and elevation data at 30 arc seconds resolution: SRTM30_PLUS. *Marine Geodesy* **32**, 355-371 (2009).
63 Hijmans, R. J., Cameron, S. E., Parra, J. L., Jones , P. G. & Jarvis, A. Very high resolution interpolated climate surfaces for global land areas. *International Journal of Climatology* **25**, 1965-1978 (2005).
64 Nettle, D. & Romaine, S. *Vanishing voices: The extinction of the world's languages.* (Oxford University Press, 2000).
65 Dalby, A. *Language in Danger.* (Columbia University Press, 2002).
66 Pakir, A. in *Sixth International Conference on Austronesian Linguistics*, (Honolulu, Hawaii, 1991).
67 Leppänen, S. & Pahta, P. Finnish culture and language endangered—language ideological debates on English in the finnish press from 1995 to 2007 in *Dangerous Multilingualism* p142-175 (Springer, 2012).
68 Brutt-Griffler, J. Language endangerment, the construction of indigenous languages and world English. *Contributions To The Sociology Of Language* **92**, 35 (2006).
69 Mühlhäusler, P. *Linguistic ecology: Language change and linguistic imperialism in the Pacific region.* (Routledge, 2002).
70 Skutnabb–Kangas, T. in *The politics of English as a world language: New horizons in postcolonial cultural studies* (ed C. Mair) 31-52 (2003).
71 Mufwene, S. S. Globalization and the myth of killer languages: What's really going on. *Perspectives on endangerment* **5**, 19-48 (2005).
72 Smith, G. & Siegel, J. in *The survey of pidgin and creole languages, Vol I* (eds S. Michaelis, P. Maurer, M. Haspelmath, & M. Huber) (Oxford University Press, 2013).
73 Aikhenvald, A. Y. in *New perspectives on endangered languages: bridging gaps between sociolinguists, documentation and language revitalisation* (eds J. A Flores Farfan & F. Ramallo) (John Benjamins Publishing Company, 2010).
74 Grineveld, C. in *Endangered languages: current issues and future prospects* (eds L. A. Grenoble & L. J. Whaley) 124-161 (Cambridge University Press, 1998).
75 Brenzinger, M. Language endangerment throughout the World. Language diversity endangered (Mouton de Gruyter, Berlin, 1992).
76 Monaka, K. C. & Chebanne, A. M. Setswana and the Building of a Nation State. *Anthropological Linguistics* **61**, 75-93 (2019).
77 Bromham, L., Hua, X., Algy, C. & Meakins, F. Language endangerment: a multidimensional analysis of risk factors. *Journal of Language Evolution* **5**, 75-91, doi:10.1093/jole/lzaa002 (2020).

78 McCarty, T. L. Between possibility and constraint: Indigenous language education, planning, and policy in the United States. *Language policies in education: Critical issues*, 285-307 (2002).

79 Simpson, J. & Wigglesworth, G. Language diversity in Indigenous Australia in the 21st century. *Current Issues in Language Planning* **20**, 67-80 (2019).

80 Combs, M. C. & Nicholas, S. E. The effect of Arizona language policies on Arizona Indigenous students. *Language Policy* **11**, 101-118 (2012).

81 Bromham, L., Hua, X., Cardillo, M., Schneemann, H. & Greenhill, S. J. Parasites and politics: why cross-cultural studies must control for relatedness, proximity and covariation. *Royal Society Open Science* **5**, 181100 (2018).

82 Bromham, L., Skeels, A., Schneemann, H., Dinnage, R. & Hua, X. There is little evidence that spicy food in hot countries is an adaptation to reducing infection risk. *Nature Human Behaviour*, doi:10.1038/s41562-020-01039-8 (2021).

83 Kaufman, T. & Golla, V. in *America Past, America Present: Genes And Languages In The Americas And Beyond* (ed C. Renfrew) 47–57 (McDonald Institute For Archaeological Research, 2000).

84 Ringe, D. "Nostratic" and the factor of chance. *Diachronica* **12**, 55–74 (1995).

85 Jäger, G. Global-scale phylogenetic linguistic inference from lexical resources. *Scientific Data* **5**, 180189, doi:10.1038/sdata.2018.189 (2018).

86 Pagel, M., Atkinson, Q. D., S. Calude, A. & Meade, A. Ultraconserved words point to deep language ancestry across Eurasia. *Proceedings of the National Academy of Sciences USA* **110**, 8471-8476, doi:10.1073/pnas.1218726110 (2013).

87 Mahowald, K. & Gibson, E. Short, frequent words are more likely to appear genetically related by chance. *Proceedings of the National Academy of Sciences* **110**, E3253-E3253, doi:10.1073/pnas.1308822110 (2013).

88 Heggarty, P. Ultraconserved words and Eurasiatic? The "faces in the fire" of language prehistory. *Proceedings of the National Academy of Sciences* **110**, E3254-E3254, doi:10.1073/pnas.1309114110 (2013).

89 Bromham, L. Solving Galton's problem: practical solutions for analysing language diversity and evolution. *Historical Linguistics* **in press** (2021).

90 Kirby, K. R. *et al.* D-PLACE: A global database of cultural, linguistic and environmental diversity. *PloS one* **11**, e0158391 (2016).

91 Tukey, J. W. *Exploratory Data Analysis.*, (Addison-Wesley, 1977).

92 Box, G. E. P. & Cox, D. R. An analysis of transformations. *Journal of the Royal Statistical Society, Series B* **26**, 211–252 (1964).

93 Dow, M. M. Network autocorrelation regression with binary and ordinal dependent variables: Galton's problem. *Cross-Cultural Research* **42**, 394 (2008).

94 Wurm, M. J., Rathouz, P. J. & Hanlon, B. M. Regularized ordinal regression and the ordinalNet R package. *<arXiv:1706.05003>* (2017).

95 Byrd, R. H., Lu, P., Nocedal, J. & Zhu, C. A limited memory algorithm for bound constrained optimization. *SIAM Journal on Scientific Computing* **16**, 1190–1208., doi:doi: 10.1137/0916069. (1995).

96 Santosa, F. & Symes, W. W. Linear inversion of band-limited reflection seismograms. *SIAM Journal on Scientific and Statistical Computing* **7**, 1307–1330, doi:doi:10.1137/0907087 (1986).

97 Cardillo, M., Mace, G. M., Gittleman, J. L. & Purvis, A. Latent extinction risk and the future battlegrounds of mammal conservation. *Proceedings of the National Academy of Sciences* **103**, 4157-4161 (2006).

98 Australian Institute of Health and Welfare. Indigenous life expectancy and deaths [accessed 18 Nov 2020] Available at: https://www.aihw.gov.au/reports/australias-health/indigenous-life-expectancy-and-deaths. (2020).

99 Deuchar, M. in *Proceedings of the 4th International Symposium on Bilingualism* (Cascadilla Press, 2005).

100 Prochazka, K. & Vogl, G. Quantifying the driving factors for language shift in a bilingual region. *Proceedings of the National Academy of Sciences* **114**, 4365-4369, doi:10.1073/pnas.1617252114 (2017).

101 MacAuley, D. in *The Celtic Languages* (ed D. MacAulay) 137-248 (Cambridge University Press, 1992).

102 Bowern, C. *A Grammar of Bardi.* (De Gruyter Mouton, 2012).

103 Battin, J. *et al. National Indigenous Languages Report.* (Commonwealth of Australia, 2020).

104 Newbold, T. *et al.* Global effects of land use on local terrestrial biodiversity. *Nature* **520**, 45-50, doi:10.1038/nature14324 (2015).

105 Chen, G. *et al.* Global projections of future urban land expansion under shared socioeconomic pathways. *Nature Communications* **11**, 537, doi:10.1038/s41467-020-14386-x (2020).

106 Kozok, U. How many people speak Indoneisan? *https://ipll.manoa.hawaii.edu/indonesian/2012/03/10/how-many-people-speak-indonesian/* **University of Hawai'i at Manoa** (2012).

107 Leclerc, J. L'aménagement linguistique dans le monde. in *http://www.axl.cefan.ulaval.ca/monde/index_alphabetique.htm* (2019).

Supplementary Table 1: Language endangerment scales, including the seven-level classification used in this study (see Supplementary Methods for details) which is based on EGIDS scores (Lewis and Simons 2010; Lewis et al. 2013). The correspondence to UNESCO (Moseley 2010), Language Endangerment Index (LEI[93]) and Agglomerated Endangered Scale (AES[8]) is based on Hammarström et al. (2018). "Sleeping" is the widely preferred term by those in the endangered languages community for languages that currently do not have L1 speakers.

Endangerment level		EGIDS			AES	UNESCO	LEI
**Stable**	1	0	International	widely used in trade, knowledge exchange, international policy.	not endangered	safe	at risk
		1	National	used in education, work, mass media, national government			
		2	Provincial	used in education, work, mass media, regional government			
		3	Wider Communication	used in work, mass media but without official status			
		4	Educational	vigorous use, with standardization and literature, supported education.			
		5	Developing	vigorous use with literature but not widespread or sustainable.			
			Dispersed	used in home country, standardized form and literature, but not promoted in education.			
		6a	Vigorous	sustainably used for face-to-face communication by all generations			
**Threatened**	2	6b	Threatened	used for face-to-face communication within all generations, but losing users.	threatened	vulnerable	vulnerable
**Endangered**	3	7	Shifting	child-bearing generation use among themselves, not transmitting to children.	shifting	definitely endangered	threatened
							endangered
**Critically Endangered**	4	8a	Moribund	used only by grandparent generation and older	moribund	severely endangered	severely endangered
	5	8b	Nearly Extinct	limited use by grandparent generation only	nearly extinct	critically endangered	critically endangered
**Sleeping**	6	9	Dormant	serves as a reminder of heritage identity but use is symbolic	extinct	extinct	dormant
			Reawakening	community working to establish users of once-dormant language			awakening
							second language only
	7	10	Extinct	not used and no current association with ethnic identity			extinct

Supplementary Table 2: World languages: nine widespread languages are not included in the analysis as languages but as variables (for details see Supplementary Methods 2.10). We use the following ISO-693 codes, some of which are "macrolanguages" covering for a number of varieties. For example, the code [ara] incorporates 30 varieties of Arabic (e.g. Algerian Saharan Arabic [aao], Tajiki Arabic [abh]) and the code incorporates 38 varieties of Malay (e.g. Indonesian [ind], Minangkabau [min]). The approximate total number of speakers includes both L1 (first language) and L2 (second language) speakers, the percentage of second language learners is given as %L2, and the number of countries in which it is recognised as an official (or *de facto* official) language at national level (see Supplementary Table 3). Speaker numbers are from Ethnologue apart from L2 speakers of Malay which are from Kozok[106].

Language	ISO	Speakers (L1+L2) (millions)	L1 (millions)	L2 (millions)	% L2	National language
Mandarin	zho	1120	921	199	18	3
English	eng	1348	370	978	73	60
Hindustani		830			50	3
(Hindi +Urdu)	hin		342	258		
	urd		69	161		
Arabic	ara	580	310	270	47	25
Spanish	spa	542	471	71	13	21
Malay (inc. Indonesian)	msa	268	44	155	58	4
French	fra	257	80	187	70	31
Russian	rus	257	153	104	40	8
Portuguese	por	257	232	25	10	9

Supplementary Table 3: Official languages, recognized at national level for each state (country, independent territory, semi-autonomous region etc: identified by ISO-3166-1 country code), and whether the state recognizes one of nine world languages as an official language (see Supplementary Table 2). Note that this list is not definitive: the official and national languages for a country are not always clear and may change over time (see Supplementary Methods 2.1.4). "De facto/recognized" includes some commonly used languages that do not have official status but act as one of the main languages of a county. We include these because they often represent world languages that may have a significant impact on the vitality of indigenous languages in that country. We record 'n' for countries that do not have one of the nine world languages as an officially recognized language or de facto recognized language.

State	IsoC	Official	National	De Facto/ Recognized	World Language
Afghanistan	AFG	Pashto, Dari			n
Albania	ALB	Albanian			n
Algeria	DZA	Arabic, Berber		French	Arabic, French
American Samoa	NA	Samoan, English			English
Andorra	NA	Catalan			n
Angola	AGO	Portuguese	Kimbundu, Umbundu, Kikongo, Chokwe, Kwanyama, Mbunda		Portuguese
Antigua and Barbuda	ATG	English			English
Argentina	ARG		Spanish		Spanish
Armenia	ARM	Armenian			n
Aruba	ABW	Dutch, Papiamento			n
Australia	AUS			English	English
Austria	AUT	German			n
Azerbaijan	AZE	Azerbaijani			n
Bahamas, The	BHS	English			English
Bahrain	BHR	Arabic		English	English, Arabic
Bangladesh	BGD	Bengali			n
Barbados	BRB	English		Bajan Creole	English
Belarus	BLR	Belarusian, Russian			Russian
Belgium	BEL	Dutch, French, German			French
Belize	BLZ	English		Belize Creole	English
Benin	BEN	French	Fon, Yom, Yoruba		French
Bermuda	BMU	English			English
Bhutan	BTN	Dzongkha			n
Bolivia	BOL	Spanish, Aymara, Araona, Baure, Bésiro, Canichana, Cavineño, Cayubaba, Chácobo, Chimán, Ese Ejja, Guaraní, Guarasu'we, Guarayu, Itonama, Leco, Machajuyai-Kallawaya, Machineri, Maropa, Mojeño-Ignaciano, Mojeño-Trinitario, Moré, Mosetén, Movima, Pacawara, Puquina, Quechua, Sirionó, Tacana, Tapieté, Toromona, Uru-Chipaya, Weenhayek, Yaminawa, Yuki, Yuracaré, Zamuco			Spanish
Bosnia and Herzegovina	BIH	Bosnian, Serbian, Croatian			n
Botswana	BWA	English, Setswana			English
Brazil	BRA	Portuguese			Portuguese

British Virgin Islands	NA	English			English
Brunei Darussalam	BRN	Malay		English	Malay, English
Bulgaria	BGR	Bulgarian			n
Burkina Faso	BFA	French	Fula, Jula, More		French
Burundi	BDI	English, French, Kirundi			French, English
Cabo Verde	CPV	Portuguese, Cape Verdean Creole			Portuguese
Cambodia	KHM	Khmer		English, French	French, English
Cameroon	CMR	English, French			French, English
Canada	CAN	English, French			French, English
Cayman Islands	CYM	English			English
Chad	TCD	Arabic, French			Arabic, French
Chile	CHL	Spanish			Spanish
China	CHN	Mandarin Chinese			Mandarin
Colombia	COL	Spanish			Spanish
Comoros	COM	Arabic, French, Comorian			Arabic, French
Congo, Dem. Rep.	COD	French	Lingala, Kingogo, Swahili, Tshiluba		French
Congo, Rep.	COG	French	Kituba, Kikongo, Lingala		French
Costa Rica	CRI	Spanish			Spanish
Cote d'Ivoire	CIV	French			French
Croatia	HRV	Croatian			n
Cuba	NA	Spanish			Spanish
Curacao	CUW	Papiamento, English, Dutch			English
Cyprus	CYP	Greek, Turkish			n
Czech Republic	CZE	Czech			n
Denmark	DNK	Danish			n
Djibouti	DJI	Somali, French, Arabic	Afar		French, Arabic
Dominica	DMA	English		Domican Creole	English
Dominican Republic	DOM	Spanish			Spanish
Ecuador	ECU	Spanish			Spanish
Egypt, Arab Rep.	EGY	Arabic			Arabic
El Salvador	SLV	Spanish			Spanish
Equatorial Guinea	GNQ	French, Portuguese, Spanish			French, Portuguese, Spanish
Eritrea	ERI		Afar, Arabic, Beja, Bilen, Kunama, Nara, Saho, Tigre	Tigrinya, English	Arabic, English
Estonia	EST	Estonian			n
Eswatini	SWZ	English, Swazi			English
Ethiopia	ETH	Amharic		English	English
Faroe Islands	NA	Faroese, Danish			n
Fiji	FJI	English, Fijian, Fiji Hindi			English, Hindustani
Finland	FIN	Finnish, Swedish			n
France	FRA	French			French
French Polynesia	NA	French			French
Gabon	GAB	French			French
Gambia, The	GMB	English			English
Georgia	GEO	Georgian			n
Germany	DEU	German			n
Ghana	GHA	English	Akuapem Twi, Asante Twi, Ewe, Dagaare, Dagbani, Dangme, Ga, Gonja, Kasem, Fante, Nzema		English
Greece	GRC	Greek			n
Greenland	NA	Greenlandic			n
Grenada	GRD	English		Grenadian Creole English, Antillean Creole	English
Guam	NA	English, Chamorro			English
Guatemala	GTM	Spanish			Spanish
Guernsey (Channel Islands)	NA	English, French			English, French
Guinea	GIN	French			French
Guinea-Bissau	GNB	Portuguese	Upper Guinea Creole		Portuguese

Guyana	GUY	English		Guyanese Creole	English
Haiti	HTI	French, Haitian Creole			French
Honduras	HND	Spanish			Spanish
Hong Kong SAR, China	HKG	English, Mandarin			English, Mandarin
Hungary	HUN	Hungarian			n
Iceland	ISL	Icelandic			n
India	IND	Hindi, English			English, Hindustani
Indonesia	IDN	Indonesian			Malay
Iran, Islamic Rep.	IRN	Persian			n
Iraq	IRQ	Arabic, Kurdish			Arabic
Ireland	IRL	English, Irish			English
Isle of Man	NA	Manx		English	English
Israel	ISR	Hebrew		Arabic	Arabic
Italy	ITA	Italian			n
Jamaica	JAM	English		Jamaican Patois	English
Japan	JPN	Japanese			n
Jersey (Channel Islands)	NA			English	English
Jordan	JOR	Arabic			Arabic
Kazakhstan	KAZ	Kazakh, Russian			Russian
Kenya	KEN	English, Swahili			English
Kiribati	KIR	English, Taatate ni Kiribati			English
Korea, Dem. Peoples Rep.	NA	Korean			n
Korea, Rep.	KOR	Korean, Korean Sign Language			n
Kosovo	XKX	Albanian, Serbian			n
Kuwait	KWT	Arabic			Arabic
Kyrgyz Republic	KGZ	Kyrgyz, Russian			Russian
Lao PDR	LAO	Lao, French			French
Latvia	LVA	Latvian			n
Lebanon	LBN	Arabic			Arabic
Lesotho	LSO	Sotho, English			English
Liberia	LBR	English			English
Libya	LBY	Arabic			Arabic
Liechtenstein	NA	German			German
Lithuania	LTU	Lithuanian			n
Luxembourg	LUX	Luxembourgish, French, German			French
Macao SAR, China	MAC	Portuguese, Mandarin			Mandarin, Portuguese
Madagascar	MDG	French, Malagasy			French
Malawi	MWI	Chewa, English			English
Malaysia	MYS	Malay		English	Malay, English
Maldives	MDV	Dhivehi		English	English
Mali	MLI	French	Bambara, Bomu, Bozo, Dogon, Fulfulde, Hassaniya Arabic, Mamara, Maninkakan, Soninke, Songhay, Syenara, Tamasheq, Xaasongaxango		French
Malta	MLT	Maltese, English			English, Arabic
Marshall Islands	MHL	Marshallese, English			English
Mauritania	MRT	Arabic	Pulaar, Soninke, Malinke, Wolof		Arabic
Mauritius	MUS			English, French	English, French
Mexico	MEX			Spanish	Spanish
Micronesia, Fed. Sts.	FSM	English			English
Moldova	MDA	Romanian			n
Monaco	NA	French			French
Mongolia	MNG	Mongolian			n
Montenegro	MNE	Montenegran			n
Morocco	MAR	Arabic, Berber			Arabic
Mozambique	MOZ	Portuguese			Portuguese
Myanmar	MMR	Burmese			n
Namibia	NAM	English	Afrikaans, German, Otjiherero, Koekhoegowab,		English

			Oshiwambo, RuKwangali, siLozi		
Nauru	NRU	Nauruan		English	English
Nepal	NPL	Nepali			n
Netherlands	NLD	Dutch			n
New Caledonia	NA	French			French
New Zealand	NZL	English, Maori, NZ Sign Language			English
Nicaragua	NIC	Spanish			Spanish
Niger	NER	French	Arabic, Buduma, Fulfulde, Gourmanchéma, Hausa, Kanuri, Zarma & Songhai, Tamasheq, Tassawaq, Tebu		French, Arabic
Nigeria	NGA	English			English
North Macedonia	MKD	Macedonian, Albanian			n
Norway	NOR	Norwegian, Sami			n
Oman	OMN	Arabic			Arabic
Pakistan	PAK	English, Urdu			English, Hindustani
Palau	PLW	Palauan, English			English
Panama	PAN	Spanish			Spanish
Papua New Guinea	PNG	English, Tok Pisin, Hiri Motu, PNG Sign Language			English
Paraguay	PRY	Spanish, Guarani			Spanish
Peru	PER	Spanish, Quechua, Amara			Spanish
Philippines	PHL	English, Filipino			English
Poland	POL	Polish			n
Portugal	PRT	Portuguese			Portuguese
Puerto Rico	PRI	Spanish, English			Spanish, English
Qatar	QAT	Arabic			Arabic
Romania	ROU	Romanian			n
Russian Federation	RUS	Russian			Russian
Rwanda	RWA	English, French, Kinharwanda, Swahili			English, French
Samoa	WSM	English, Samoa			English
San Marino	SMR	Italian			n
Sao Tome and Principe	STP	Portuguese			Portuguese
Saudi Arabia	SAU	Arabic			Arabic
Senegal	SEN	French			French
Serbia	SRB	Serbian			n
Seychelles	SYC	English, French, Seychellois Creole			English, French
Sierra Leone	SLE	English			English
Singapore	SGP	English, Mandarin, Malay, Tamil			English, French, Mandarin
Sint Maarten (Dutch part)	SXM	Dutch, English			English
Slovak Republic	SVK	Slovak			n
Slovenia	SVN	Slovene			n
Solomon Islands	SLB	English			English
Somalia	NA	Somali, Arabic			Arabic
South Africa	ZAF	Ndebele, Northern Sotho, Sotho, SiSwati, Tsonga, Tswana, Venda, Xhosa, Zulu and Afrikaans.		English	English
South Sudan	SSD	English, Swahili		Arabic	English, Arabic
Spain	ESP	Spanish			Spanish
Sri Lanka	LKA	Sinhala, Tamil		English	English
St. Kitts and Nevis	KNA	English			English
St. Lucia	LCA	English			English
St. Martin (French part)	NA	French			French
St. Vincent and the Grenadines	VCT	English			English
Sudan	SDN	English, Arabic			English, Arabic
Suriname	SUR	Dutch			n
Sweden	SWE	Swedish	Finnish, Meänkieli, Romani, Sami, Yiddish		n
Switzerland	CHE	German, French, Italian, Romansch			French
Syrian Arab Republic	NA	Arabic			Arabic
Taiwan	NA			Mandarin	Mandarin

Tajikistan	TJK	Tajik		Russian	Russian
Tanzania	TZA	Swahili, English			English
Thailand	THA	Thai			n
Timor-Leste	TLS	Portuguese, Tetum			Portuguese
Togo	TGO	French	Ewe, Kabiye		French
Tonga	TON	English, Tongan			English
Trinidad and Tobago	TTO	English			English
Tunisia	TUN	Arabic		French	Arabic, French
Turkey	TUR	Turkish			n
Turkmenistan	TKM	Turkmen			n
Turks and Caicos Islands	TCA	English			English
Tuvalu	TUV	English, Tuvaluan			English
Uganda	UGA	English, Swahili			English
Ukraine	UKR	Ukranian			n
United Arab Emirates	ARE	Arabic			Arabic
United Kingdom	GBR			English	English
United States	USA			English	English
Uruguay	URY	Spanish			Spanish
Uzbekistan	UZB	Uzbek		Russian	Russian
Vanuatu	VUT	English, French, Bislama			English, French
Venezuela, RB	VEN	Spanish			Spanish
Vietnam	VNM	Vietnamese			n
Virgin Islands (U.S.)	*NA*	English			English
West Bank and Gaza	PSE			Hebrew, Arabic	Arabic
Yemen, Rep.	YEM	Arabic			Arabic
Zambia	ZMB	English			English
Zimbabwe	ZWE	Chewa, Chibarwe, English, Kalanga, Koisan, Nambya, Ndau, Ndebele, Shangani, Shona, sign language, Sotho, Tonga, Tswana, Venda, Xhosa.			English

Supplementary Table 4. Additional sources of socioeconomic and education data. Values of these variables are taken from the World Bank for most countries, in some cases derived from aggregate values where country-specific values were not available. Where data on particular countries was missing it was supplemented from alternative sources, such as the CIA World Factbook (Central Intelligence Agency, Washington, DC: 2019).

Variable	Territory	Supplementary source
GDPpc	North Korea	CIA World Factbook 2009 estimate
	Somalia	CIA World Factbook 2010 estimate
	Syria	CIA World Factbook 2010 estimate
Education_Spending	China	China Ministry of Education https://moe.gov.cn/srcsite/A05/s3040/20111223_128871.html
	Bahamas	World Bank – Caribbean Small States aggregate
	United Arab Emirates	CIA World Factbook 2009 estimate
Life_Expectancy	Andorra	CIA World Factbook 2010 estimate
	Dominica	World Bank – Caribbean Small States aggregate
	F.S. Micronesia	World Bank – Pacific Islands Small States aggregate
	Marshall Islands	World Bank – Pacific Islands Small States aggregate
	Monaco	CIA World Factbook 2010 estimate
	Nauru	World Bank – Pacific Islands Small States aggregate
	Tuvalu	World Bank – Pacific Islands Small States aggregate
	Solomon Islands	World Bank – Pacific Islands Small States aggregate
	St. Kitts & Nevis	World Bank – Caribbean Small States aggregate
	Vanuatu	World Bank – Pacific Islands Small States aggregate
Multiple	Northern Cyprus	Indicators filled from World Bank values for Cyprus
	Kashmir	Average from World Bank values for China, Pakistan and India
	San Marino	Life_Expectancy@60, GINI and Schooling filled from Italy
	Somaliland	Indicators filled from World Bank values for Somalia
	Taiwan	All indicators sourced from Taiwan Ministry of Education https://www.edu.tw/ and Taiwan Ministry of the Interior https://www.moi.gov.tw/english/index.aspx
	Western Sahara	Indicators filled from World Bank values for Mauritania

Supplementary Table 5. Legal provision for minority education. Laws and constitutional (Const.) articles considered positive evidence of legal provision for minority education for the Minority_Education variable. The texts considered are the English or French texts provided in the *L'aménagement Linguistique dans le Monde* database[107]. Numbers after the name of the law refer to article and clause

Territory	Legal provision for minority language education
Albania	Const 20.2; Protection against Discrimination Law 18.2 (2010)
Angola	Basic Law on the Education System 9.3
Argentina	Const 75.17 (1994)
Armenia	Language Law 2.2.; Education Law 17.1 (1999)
Azerbaijan	Const 45 (1995)
Belarus	Const 50.3; Language Law 21.3 (1998)
Benin	Law 2003-17 8,23 (2003)
Bolivia	Const 78.2 (2009); Ley General de Derechos y Politicas Linguisticas 12.1. (2012)
Bosnia and Herzegovina	Law on the protection of the rights of national minorities 14.1 (2003); Law on the protection of the rights of national minorities in the FBIH 10.2 (2008); Law on the protection of the rights of national minorities (2004) (RS)
Brazil	Const 219 (1994)Law 9.394 establishing the directive and basis of national education 32.3 (1996)
Burkina Faso	Law 013-2007-AN 10.1 (2007)
Chad	Loi portant orientation du systeme educatif tchadien 5, 25 (2006)
Chile	Ley Indigena 32
China	Regional Ethnic Autonomy Law 36, 37.3 (1984)
Colombia	Const 10.3 (2001); Ley 115 57
Democratic Republic of the Congo	Loi-cadre 14/004 9.13 (2004)
Croatia	Constitutional law on the rights of national minorities 11.1 (2002); Law on education in the language and alphabet of the national minorities 1.1 (2000)
Czech Republic	Charter on human rights and basic liberties 25.2 (1992)
Djibouti	Law 96/AN/00/4e 5.1 (2000)
Ecuador	Const 346.9 (2008)
Estonia	Const 37.4 (2014)
Finland	Basic Education Law 12 (1998)
France	Arrete du 30 mai 2003/25 Juillet (2007) 1
Georgia	General education law 3 (2005)
Guatemala	Const 76 (1993); Regulation on the Law on Literacy 137-91 3
Honduras	Accuerdo presidencial no 0719-EP (1994), Decreto 93-97 (1997)
Hungary	Const 29.1 (2011)
India	Const 350A (1950)
Iraq	Const 4.1 (2005)
Kazakhstan	Const 19.2 (1995); Education Law 9.3 (2007)
Kosovo	Const 59.3 (2008)
Kyrgyzstan	Language Law 21.1 (1989)
Lithuania	Education Law 1.2 (1991)

Macao SAR, China	Law 9/2006 on the foundations of the non-university education system 37.2 (2006)
Mauritius	Education Act 1First Schedule 43.1 (1957)
Mexico	Ley General de Derechos Linguisticos de los Pueblos Indigenas 11 (2003)
Moldova	Const 35.2 (1994)
Mongolia	Const 8 (1992)
Montenegro	Const 79.4 (2007), Loir sur l'exercice des droits et libertes des minorites nationales et ethniques au Montenegro 79.4 (2004)
Myanmar	National Education Act 43.b (2014)
Namibia	Constitution 3.2 (1990)
New Caledonia	Loi 99-209 215.2 (1999)
Nicaragua	Const 121.5 (1987)
Macedonia	Const 48.4 (1991)
Norway	Act Relating to Primary and Secondary Education 2-8.1 (2005)
Panama	Law 34 modifying the Organic Law on Education No. 47 of 1946 11 (1995)
Papua New Guinea	Education Act Preliminary 2 (Amended 1995)
Paraguay	Ley de lenguas 9.6 (2010)
Peru	Ley general de Educacion 20 (2005)
Philippines	Indigenous Peoples Rights Act 30 (1997)
Poland	Law on national and ethnic minorities and on regional languages 17 (2005)
Romania	Const 32.3 (1995)
Russia	Const 26.2 (1993)
Republic of Serbia	Const 13.1 (2004)
Slovakia	Const 34.2 (1992)
Tajikistan	Language Law 21 (1989) and Law on Education of Aboriginal Peoples (2014)
Turkmenistan	Language Law 19.1 (1990)
Uganda	Const 6.3 (1995)
Ukraine	Const 53 (1996)
United States	Native American Languages Act (1990)
Uzbekistan	Law on the official language 6.2 (1995)
Venezuela, RB	Decreto Presidencial No 1.795 1 (2002); Ley de Idiomas Indígenas 7 (2008)

Supplementary Table 6: World Bank indicators used in calculating socioeconomic and education variables.

World Bank indicator	World Bank description	Related variable	Calculation
SP.DYN.LE.60.FE.IN	Life Expectancy at Age 60, female (years)	life expectancy 60	2012 value averaged with male data, weighted by population
SP.DYN.LE.60.MA.IN	Life Expectancy at Age 60, male (years)	life expectancy 60	2012 value averaged with female data, weighted by population
SP.POP.6064.FE	Population ages 60-64, female	life expectancy 60	2012 value used to weight female LE60
SP.POP.6064.MA	Population ages 60-64, male	life expectancy 60	2012 value used to weight male LE60
BAR.SCHL.25UP	Barro-Lee: Average years of total schooling, age 25+, total	years of schooling	2010 value
UIS.EA.MEAN.1T6.AG25T99	UIS: Mean years of schooling (ISCED1 or higher) of the population age 25+. Total	years of schooling	2005-15 median used for missing Barro-Lee values
SE.XPD.TOTAL.GD.ZS	Government expenditure on education, total (% of GDP)	education spending	2005-15 median
SP.URB.TOTL.ZS	Percentage of Population in Urban Areas (in % of total Population)	urban change	2017 value – 1960 value
NY.GDP.PCAP.KD	GDP per capita (constant 2010 US$)	GDPpc	2005-15 median

Supplementary Table 7: Modelling change in endangerment status over time. To predict language endangerment status at two future time steps (2060 and 2100), we first adjust the endangerment level (EGIDS) according to the rules describe in "Generational Shift", then we adjust the values of the variable L1 using the "Demographic" transition model. In addition, we have also modelled predictions with further adjustments for future values of environment and land use (which will only influence the regions where those predictors are significant).

**1. Generational shift**	**+40 years (2060)**	**+80 years (2100)**
$EGIDS_{current}$	***$EGIDS_{2060}$***	***$EGIDS_{2100}$***
6b: L1>1000	6b	6b
6b: L1≤1000, AES ≥ Shifting	7	8
7	8a	10
8a, 8b, 9,10	10	10
**2. Model Predictions**		
2.1 Demographic		
$EGIDS_{current}$	***$L1\_pop_{2060}$***	***$L1\_pop_{2100}$***
6b	$L1\_pop_{current}$*0.7	$L1\_pop_{2060}$*0.7
7	$L1\_pop_{current}$*(1-Proportion >65[i])	0
8a, 8b, 9,10	0	0
2.2. Environment		
Temperature seasonality	WorldClim 370 SSP, 2060	WorldClim 370 SSP, 2100
2.3 Land use		
Population density	pop density + (pop density change*40)	pop density + (pop density change*80)
Human footprint	human footprint + (footprint change*40)	human footprint + (footprint change*80)
Cropland	cropland + (cropland change*40)	cropland + (cropland change*80)
Built environment	built + (built change*40)	built + (built change*80)
Pasture	pasture + (pasture change*40)	pasture + (pasture change*80)

[i] *See Supplementary Table 8 for the regional values for proportion over 65*

Supplementary Table 8: Demographic data used in future predictions to adjust L1 speaker populations (see Supplementary Table 7). Data from The World Bank (2019) World Development Indicators, http://data.worldbank.org/data-catalog/world-development-indicators.

UN Region/Territory	% Population over 65
Australia & New Zealand	15.9
East Asia & Pacific	11.2
Europe & Central Asia	16.6
Latin America & Caribbean	8.7
Middle East & North Africa	5.3
North America	16.4
Papua New Guinea	3.5
Pacific island small states	4.6
South Asia	6.0
Sub-Saharan Africa	3.0

Supplementary Figure 1: Relationships between language endangerment scales (see Supplementary Methods (section 1) for details). Distribution of recorded endangerment levels for each language ("scores") are on the diagonal (bar charts): the category NA reflects the number of languages that do not have an endangerment score recorded under that particular scheme. The pairwise correlation between scores is given in the upper right hand entries. The plots in the left hand panels represent the matching of language endangerment scales, with the size and opacity of the circles representing the proportion of all languages that fall in that category. EGIDS levels were derived from Ethnologue (e16/e17), and AES were derived from Glottolog (version 4). LEI values are from the Endangered Languages Project (http://www.endangeredlanguages.com/). UNESCO scores are from the UNESCO Languages Atlas (http://www.unesco.org/languages-atlas/).

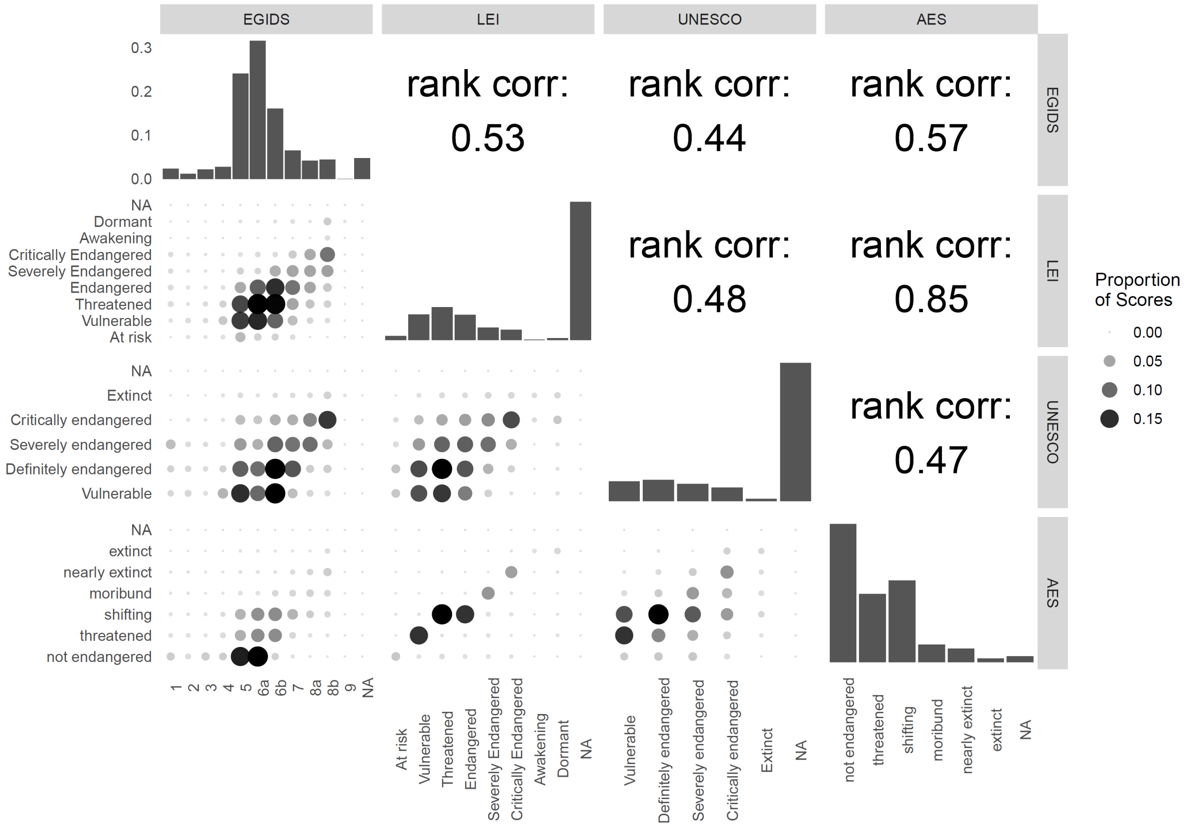

Supplementary Figure 2. Endangerment scales and L1 speaker population size. The relationship between two endangerment scales - AES (used in Glottolog) and EGIDS (used in Ethnologue) - and L1 speaker population size. AES assigns more languages with large speaker populations in highly endangered categories (extinct, nearly extinct, moribund). Some languages that are outliers are identified by ISO code. Note that these databases are constantly being updated so that this figure represents a snapshot of the data at the time of access (Ethnologue v.17 plus v.16 values for "extinct" languages, Glottolog v4.2.1). For example, Kannada [kan], a Dravidian language of south western India, was previously listed as Nearly Extinct in Glottolog despite having 44 million L1 speakers and 23 million L2 speakers, with the source cited for the rating is the UNESCO scale (Moseley 2010), possibly because the sublanguage Kuruba is listed as Critically endangered, however it has now been changed to Not Endangered.

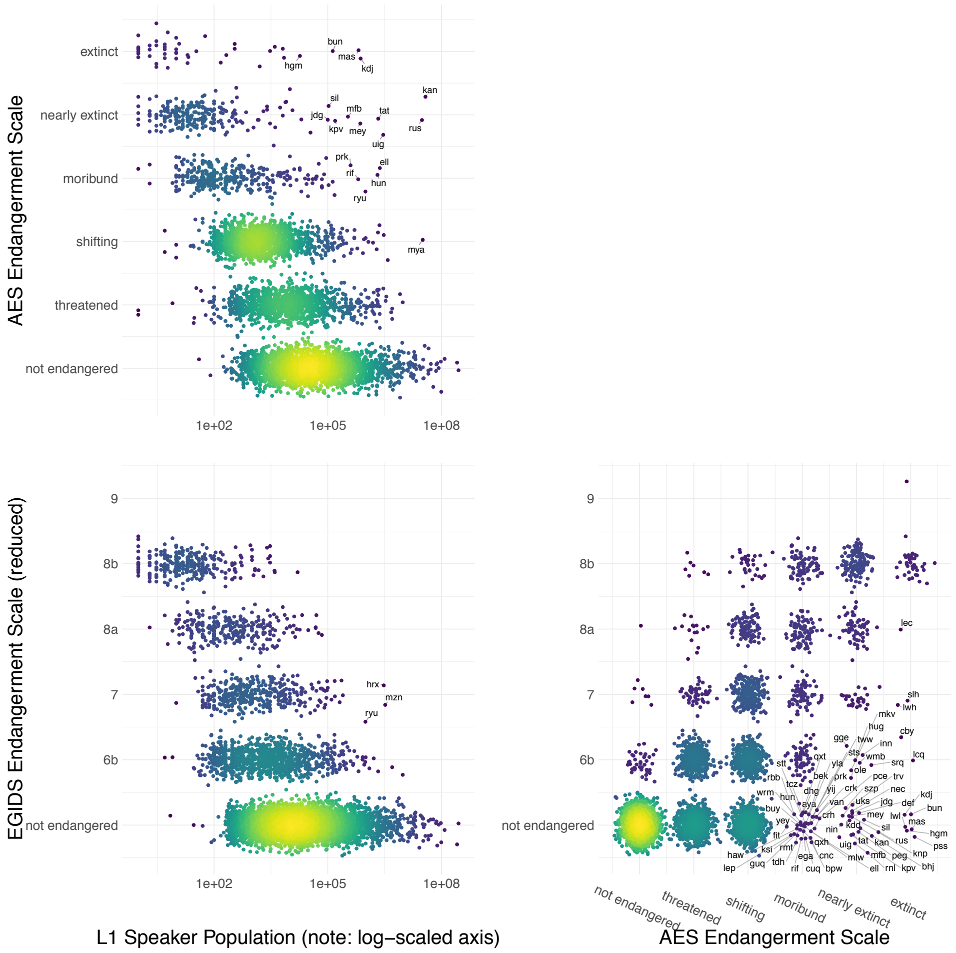

Supplementary Figure 3: Variables included as predictors in our analysis of language endangerment. Black stars indicate variables consistently selected as significant predictors in best models at a global level, white stars indicate variables consistently selected as significant predictors for one or more regions. Details of variables given in Supplementary Methods and Table 1.

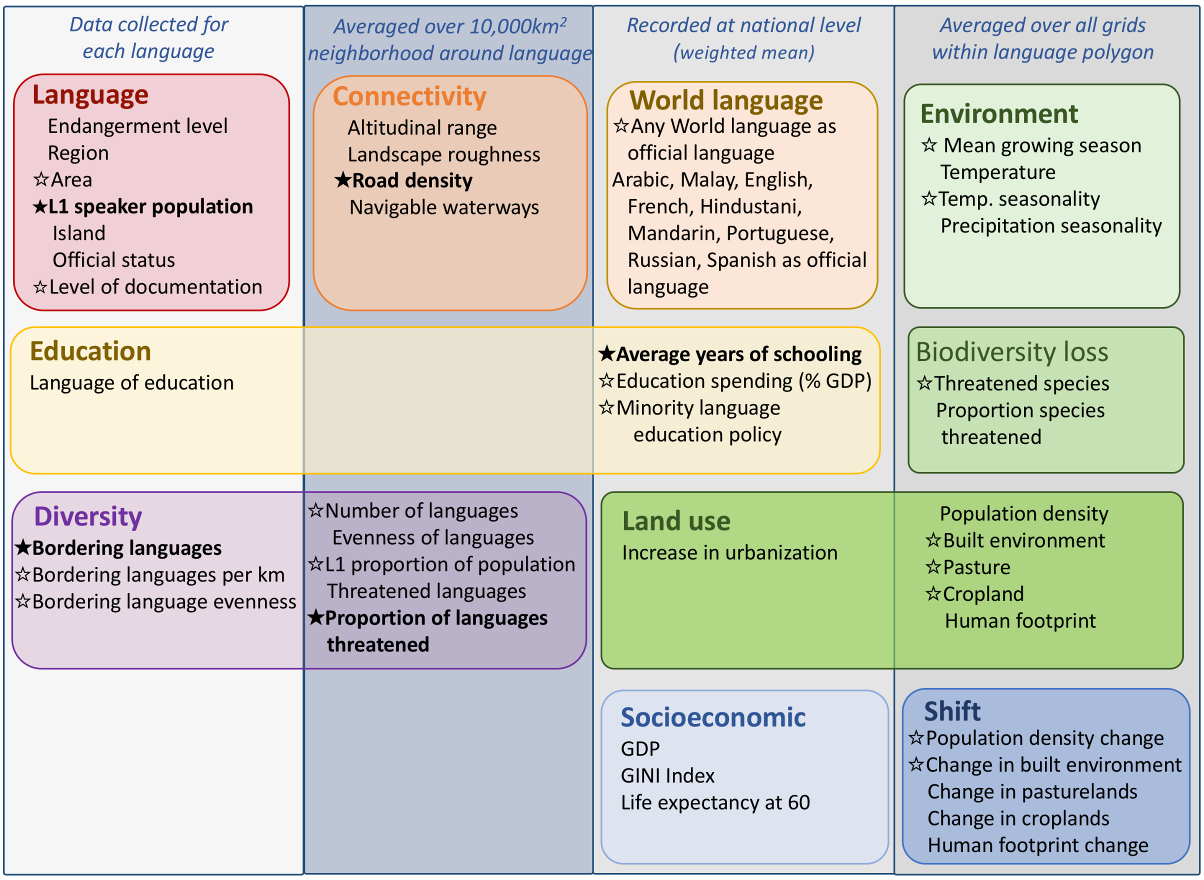

Supplementary Figure 4. Pairwise correlation coefficients between variables.

Variables between two bars are grouped together in the model selection procedure. For details of variables see Table, Supplementary Data 1.

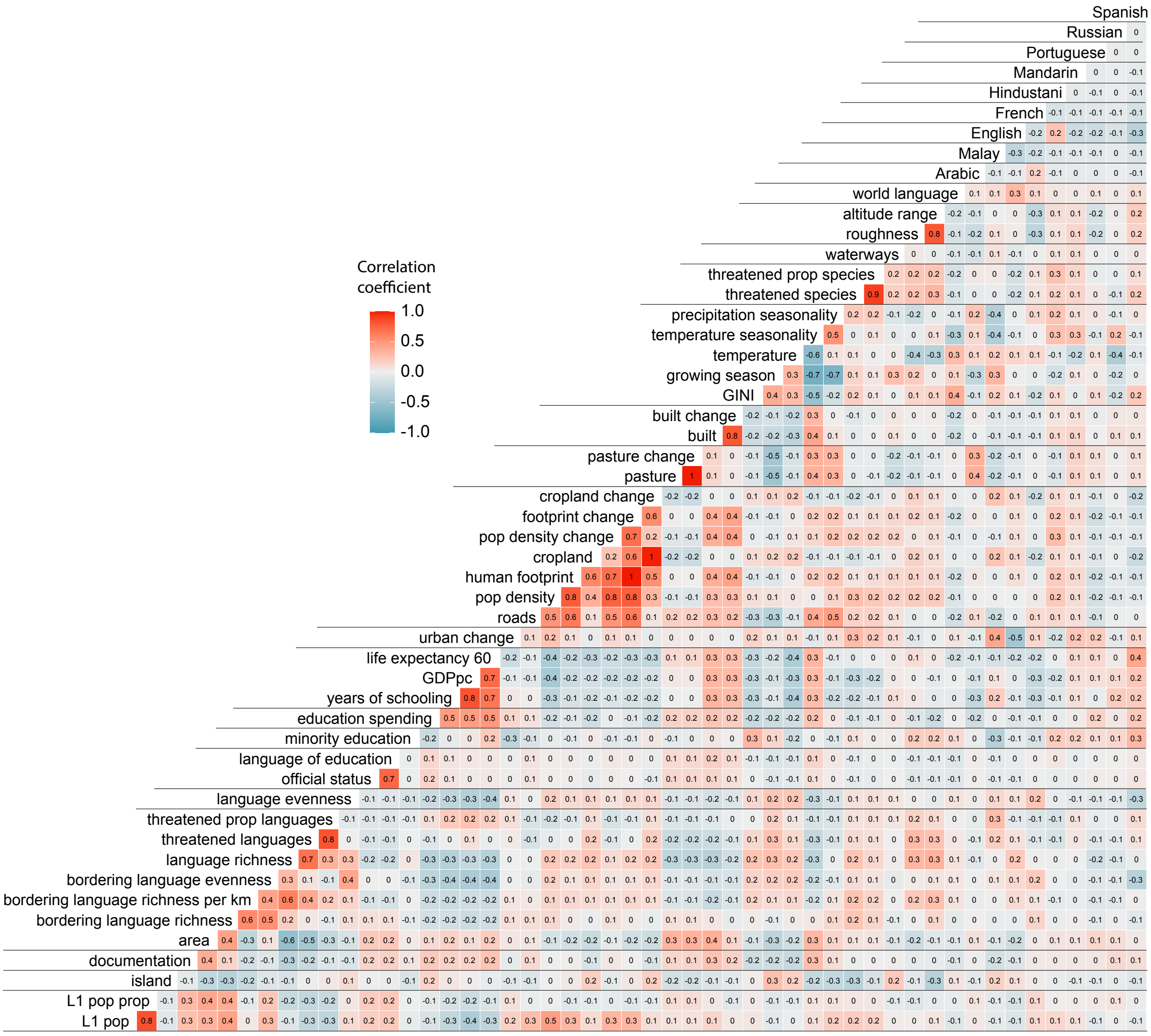

Supplementary Figure 5: Relative number of languages rated as endangered (EGIDS 6b-10) or Sleeping (EGIDS 9-10) by region. Regions are defined according to the Natural Earth dataset (see Supplementary Methods 2.1.3, Supplementary Data 1). Note that these regions were aggregated into 12 regions for the analysis, see Figure 1.

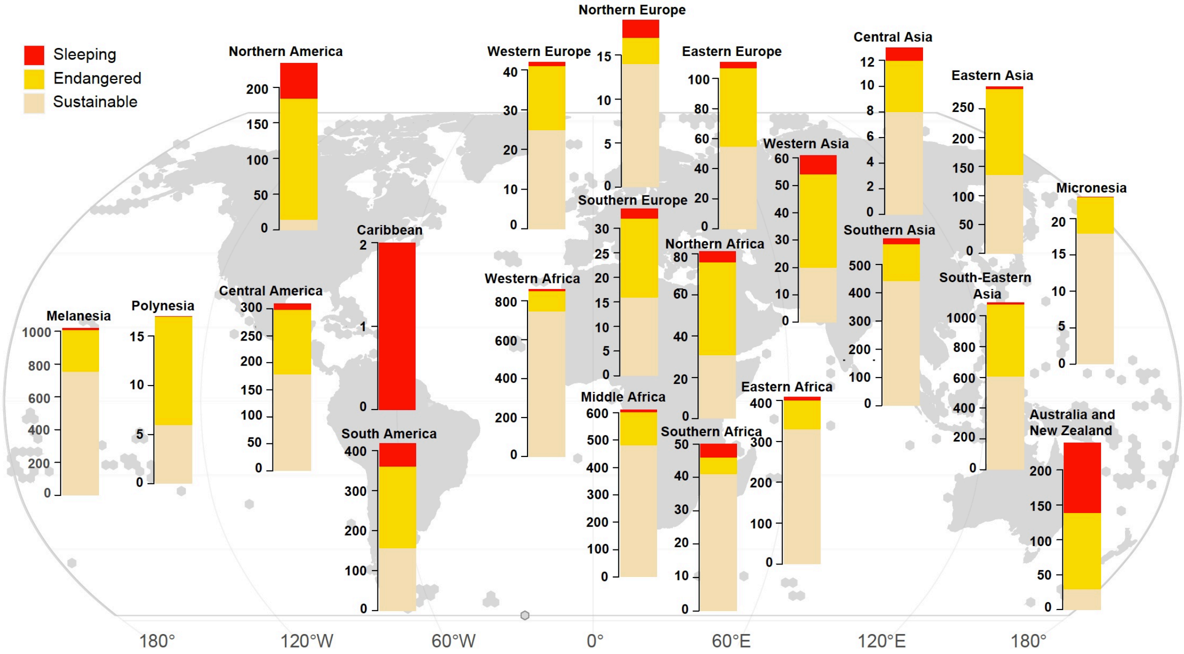

Supplementary Figure 6. Comparison of observed and predicted levels of endangerment by the best fit model. The violin plot shows the reported EGIDS from the database on the x-axis against the expected value of the endangerment level of each language predicted by the best model, with dot and whisker indicating median and interquartile range. The prediction is best for Stable (1-6a) and languages classified by EGIDS as "Extinct" (10), and those in the Threatened (6b) category.

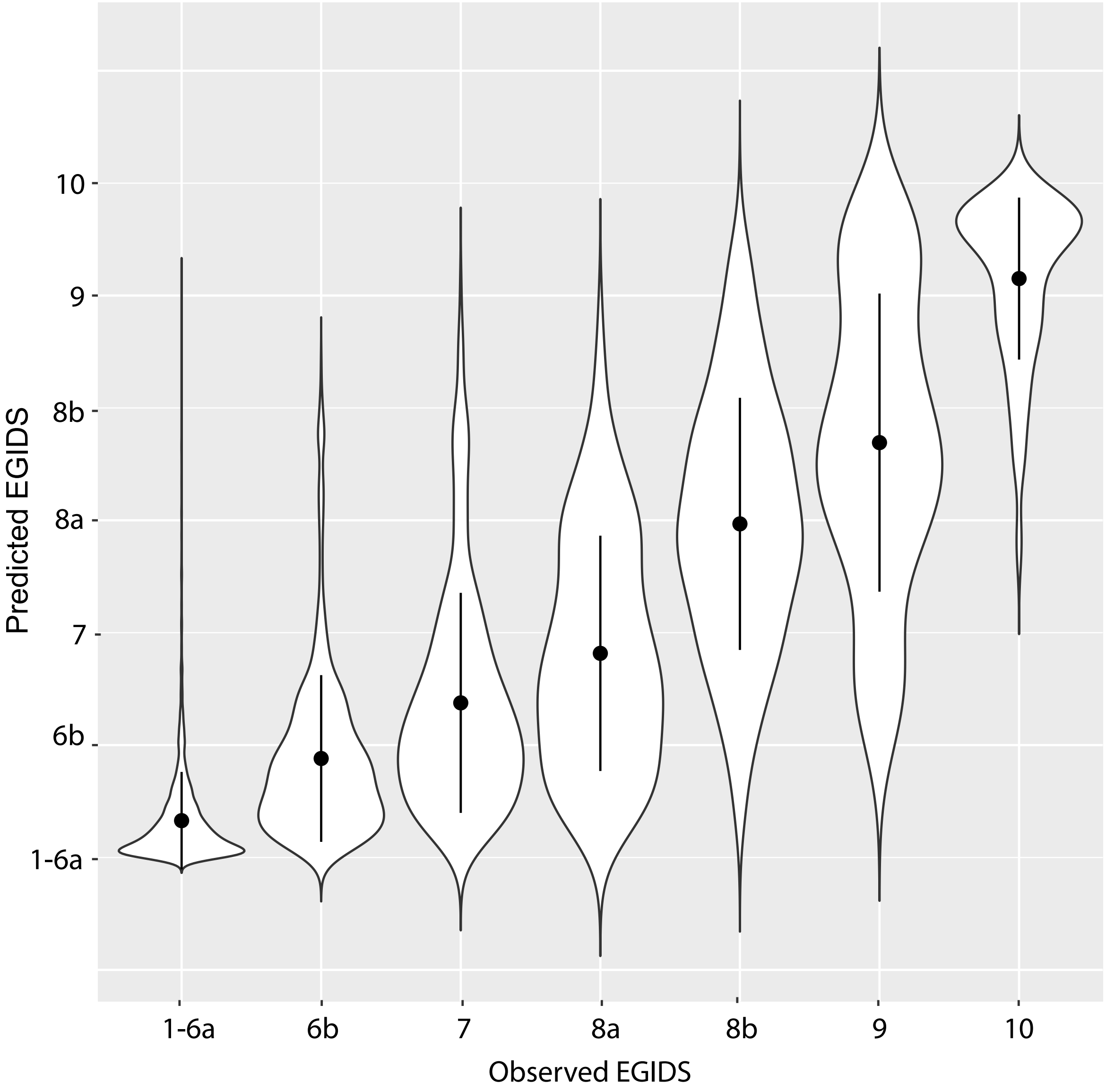

Supplementary Figure 7: Predictor variables identified in best-fit models. Each column corresponds to one of the equally best-fit models. Each row corresponds to a predictor variable. Each grid is colored by the coefficient of a predictor variable in a model, with blue indicating negative effect, red indicating positive effect, and color intensity indicating the magnitude of the effect, which is measured as the number of standard deviation change in the endangerment level given one standard deviation increase in the predictor variable. Predictor variables that are chosen in more than one third of the models are included in the best model as shown in Figure 2.

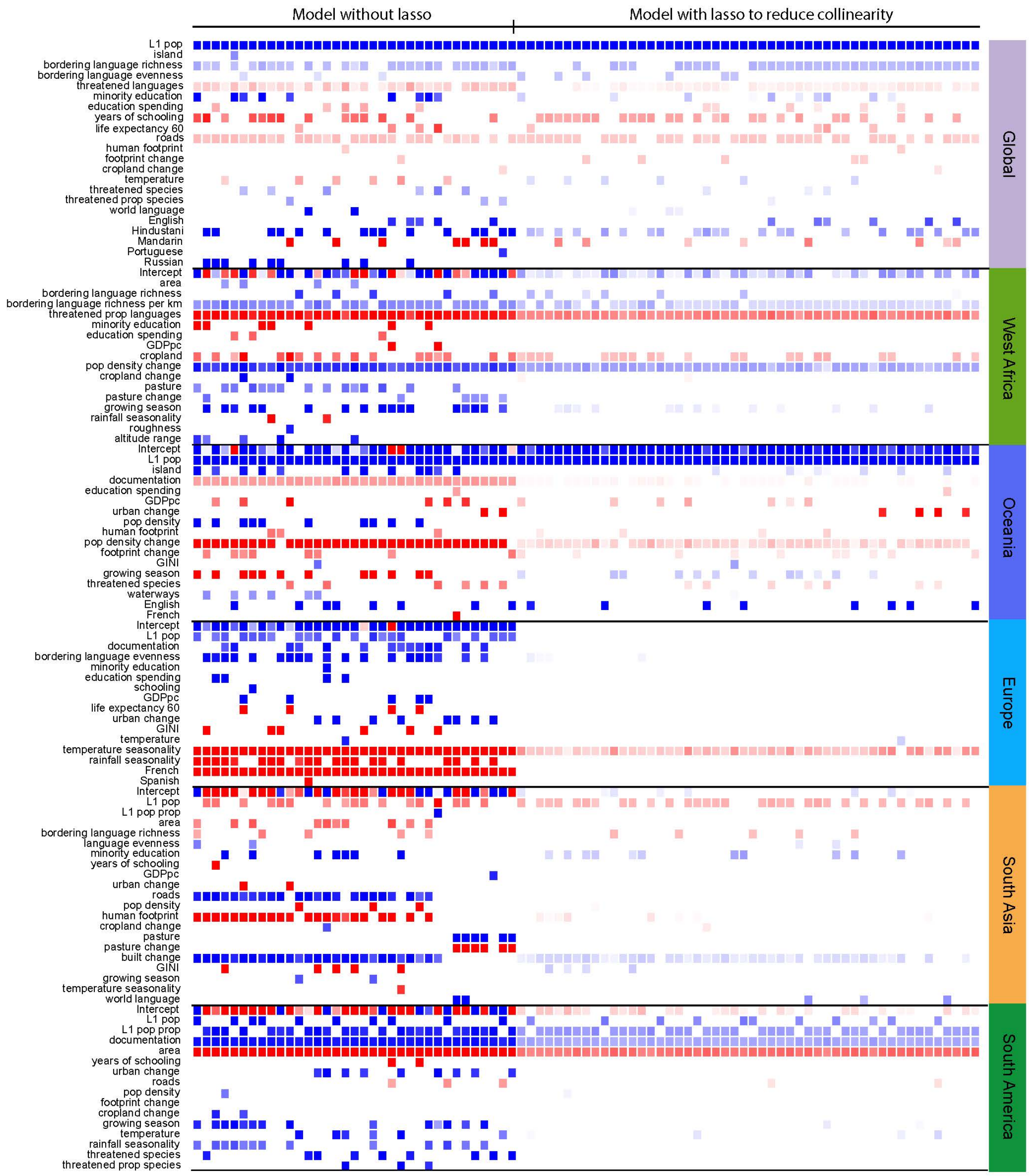
Model without lasso
Model with lasso to reduce collinearity
L1 pop
island
bordering language richness
bordering language evenness
threatened languages
minority education
education spending
years of schooling
life expectancy 60
roads
human footprint
footprint change
cropland change
temperature
threatened species
threatened prop species
world language
English
Hindustani
Mandarin
Portuguese
Russian
Global
Intercept
area
bordering language richness
bordering language richness per km
threatened prop languages
minority education
education spending
GDPpc
cropland
pop density change
cropland change
pasture
pasture change
growing season
rainfall seasonality
roughness
altitude range
West Africa
Intercept
L1 pop
island
documentation
education spending
GDPpc
urban change
pop density
human footprint
pop density change
footprint change
GINI
growing season
threatened species
waterways
English
French
Oceania
Intercept
L1 pop
documentation
bordering language evenness
minority education
education spending
schooling
GDPpc
life expectancy 60
urban change
GINI
temperature
temperature seasonality
rainfall seasonality
French
Spanish
Europe
Intercept
L1 pop
L1 pop prop
area
bordering language richness
language evenness
minority education
years of schooling
GDPpc
urban change
roads
pop density
human footprint
cropland change
pasture
pasture change
built change
GINI
growing season
temperature seasonality
world language
South Asia
Intercept
L1 pop
L1 pop prop
documentation
area
years of schooling
urban change
roads
pop density
footprint change
cropland change
growing season
temperature
rainfall seasonality
threatened species
threatened prop species
South America

Supplementary Figure 7 cont.

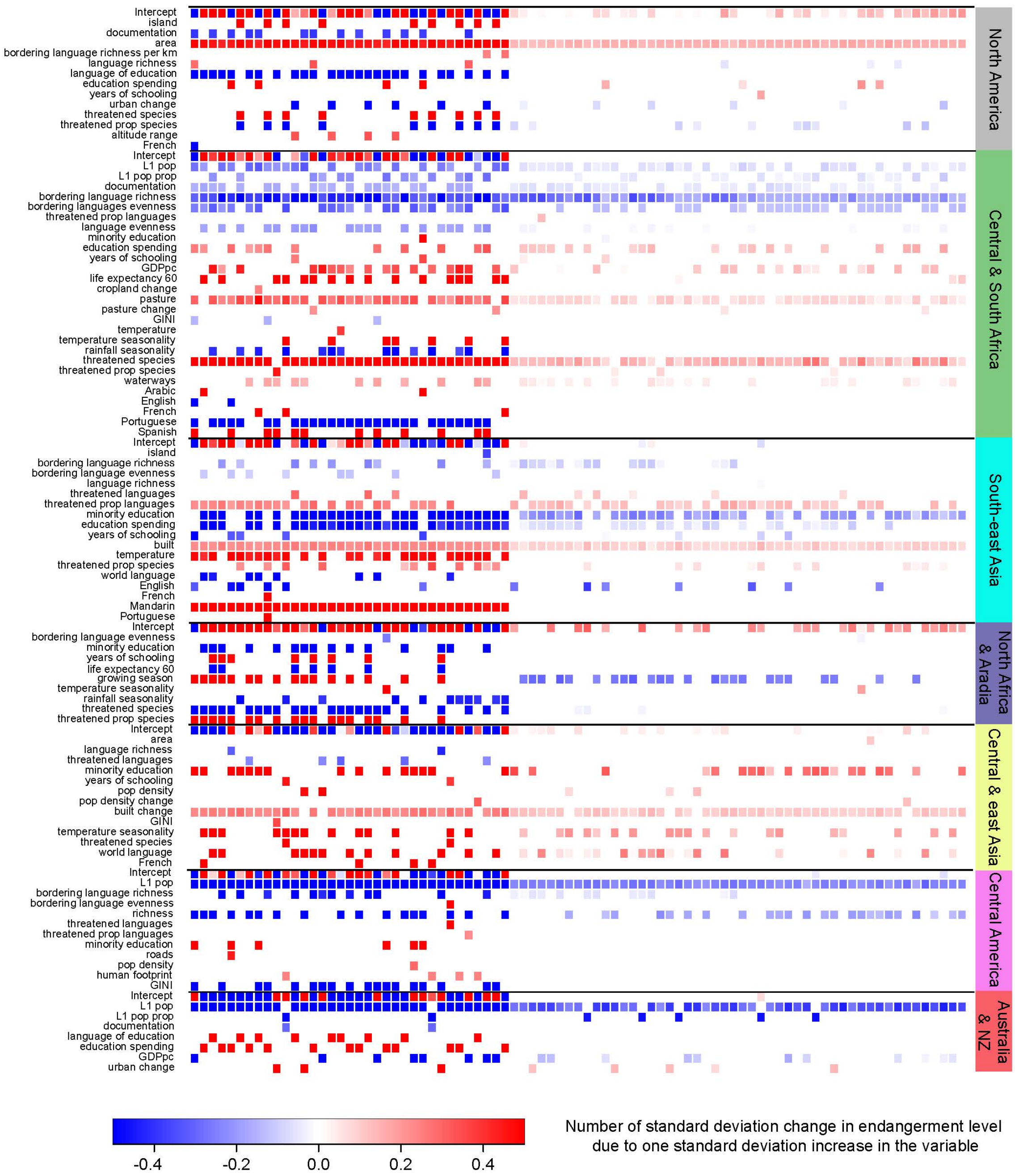

Supplementary Figure 8: Predicted patterns of language endangerment under generational shift, demographic transition, land-use shift and climate change. The proportion of all languages that are predicted to be endangered or no longer spoken in eighty years' time (A), and the current degree of documentation for these languages (B). Each violin gives the probability distribution of the number or proportion of languages that are predicted to be endangered (predicted EGIDS ≥ 6b; blue) or Sleeping (predicted EGIDS ≥ 9; red), with the dot showing the mean and the whisker showing the standard deviation. Each dashed line shows the number or proportion of languages that are currently endangered (reported EGIDS ≥ 6b; blue) or Sleeping (reported EGIDS ≥ 9; red). Threatened (EGIDS 6b), Endangered (7) and Critically Endangered (8a, 8b) languages currently have living L1 speakers, but without intervention to support language vitality, many threatened languages are predicted to lose all L1 speakers (Sleeping: EGIDS 9, 10) (Supplementary Table 1). This figure projects current levels of documentation for each language, so does not reflect future documentation efforts of endangered languages.

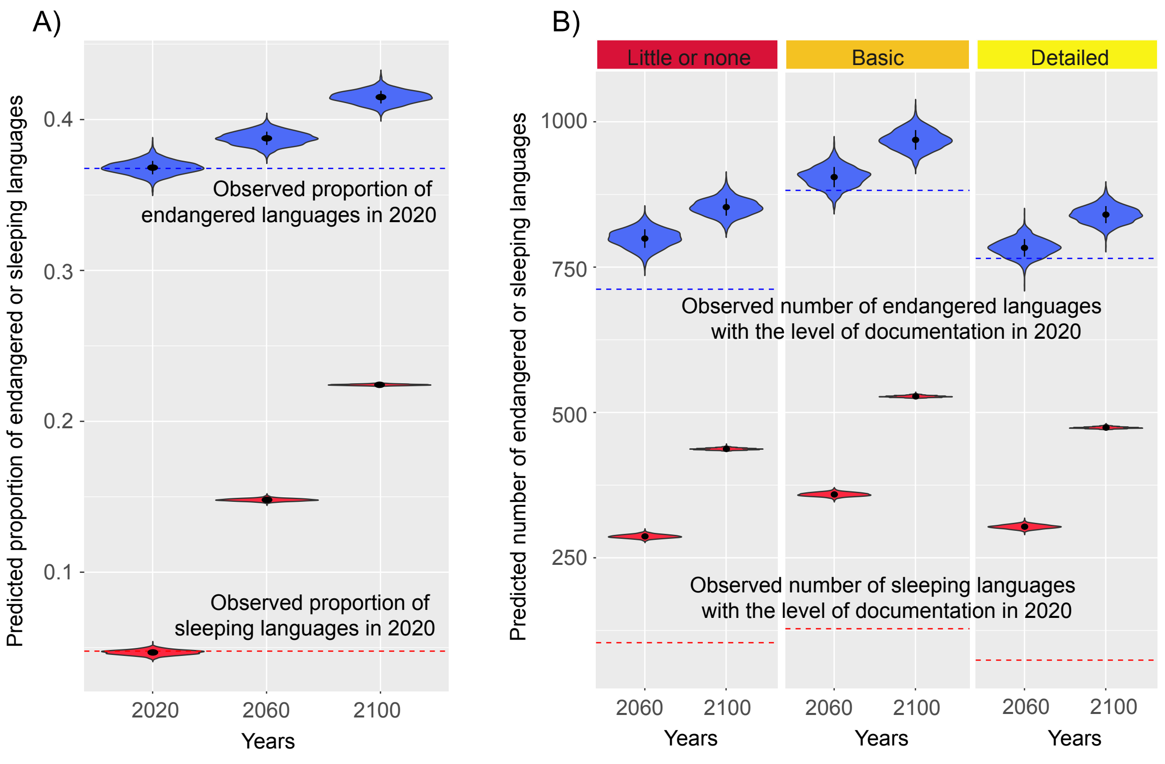

Supplement: Supplementary file 1 — Supplementary Information. [file 41559_2021_1604_MOESM1_ESM.pdf]
