# Supplementary material for: Global predictors of language endangerment and the future of linguistic diversity
Source: Nat Ecol Evol. 2021 Dec 16;6(2):163–73. doi: 10.1038/s41559-021-01604-y (PMC8825282; doi:10.1038/s41559-021-01604-y)
Supplement: Supplementary file 3 — Peer Review Information [file 41559_2021_1604_MOESM3_ESM.pdf]

Peer Review Information

Journal: Nature Ecology & Evolution
Manuscript Title: Global predictors of language endangerment and the future of linguistic diversity
Corresponding author name(s): Lindell Bromham

Editorial Notes:

Reviewer Comments & Decisions:

Decision Letter, initial version:

8th June 2021

*Please ensure you delete the link to your author homepage in this e-mail if you wish to forward it to your co-authors.

Dear Professor Bromham,

Your Article, "Global drivers of language endangerment and the future of linguistic diversity" has now been seen by four reviewers. You will see from their comments copied below that while they find your work of considerable potential interest, they have raised quite substantial concerns that must be addressed. In light of these comments, we cannot accept the manuscript for publication, but would be very interested in considering a revised version that addresses these serious concerns.

We hope you will find the reviewers' comments useful as you decide how to proceed. If you wish to submit a substantially revised manuscript, please bear in mind that we will be reluctant to approach the reviewers again in the absence of major revisions.

In particular, the reviewers all raise concerns regarding the validity of the variables examined--in some cases these may be bolstered with additional discussion and transparency, but where these are used as the basis for future predictions, there may be more fundamental issues, as reviewers 1 and 4 comment. To this end we recommend that in your revision, the focus on future predictions be removed to focus on the current status of endangered languages.

If you choose to revise your manuscript taking into account all reviewer and editor comments, please highlight all changes in the manuscript text file [OPTIONAL: in Microsoft Word format].

We are committed to providing a fair and constructive peer-review process. Please do not hesitate to contact us if there are specific requests from the reviewers that you believe are technically impossible

or unlikely to yield a meaningful outcome.

If revising your manuscript:

* Include a “Response to reviewers” document detailing, point-by-point, how you addressed each referee comment. If no action was taken to address a point, you must provide a compelling argument. This response will be sent back to the referees along with the revised manuscript.

* If you have not done so already we suggest that you begin to revise your manuscript so that it conforms to our Article format instructions at http://www.nature.com/natecolevol/info/final-submission. Refer also to any guidelines provided in this letter.

* Include a revised version of any required reporting checklist. It will be available to referees (and, potentially, statisticians) to aid in their evaluation if the manuscript goes back for peer review. A revised checklist is essential for re-review of the paper.

Please use the link below to submit a revised paper:

[REDACTED]

Note: This URL links to your confidential home page and associated information about manuscripts you may have submitted, or that you are reviewing for us. If you wish to forward this email to co-authors, please delete the link to your homepage.

If you wish to submit a suitably revised manuscript we would hope to receive it within 6 months. If you cannot send it within this time, please let us know. We will be happy to consider your revision so long as nothing similar has been accepted for publication at Nature Ecology & Evolution or published elsewhere.

Nature Ecology & Evolution is committed to improving transparency in authorship. As part of our efforts in this direction, we are now requesting that all authors identified as ‘corresponding author’ on published papers create and link their Open Researcher and Contributor Identifier (ORCID) with their account on the Manuscript Tracking System (MTS), prior to acceptance. This applies to primary research papers only. ORCID helps the scientific community achieve unambiguous attribution of all scholarly contributions. You can create and link your ORCID from the home page of the MTS by clicking on ‘Modify my Springer Nature account’. For more information please visit please visit www.springernature.com/orcid.

Please do not hesitate to contact me if you have any questions or would like to discuss the required revisions further.

Thank you for the opportunity to review your work.

[REDACTED]

Reviewer expertise:

Reviewer #1: macroecological methods

Reviewer #2: endangered languages

Reviewer #3: endangered languages

Reviewer #4: macroecological methods

Reviewers’ comments:

Reviewer #1 (Remarks to the Author):

found the paper quite interesting and well-written. The authors make a strong, clear case for the importance of understanding language endangerment. As an outsider to the linguistic literature, I am unable to evaluate many of the domain-specific portions of the analysis. Overall, I felt the analysis presented was very comprehensive and potentially very useful for elucidating drivers of linguistic loss.

As a general comment, I am a bit confused by the future projection section. First, the authors did not make the conceptual link between climate and linguistic diversity clear, therefore it was difficult to follow why we might expect future linguistic loss to be driven by changes in climate. Second, in the first section regarding drivers of language endangerment the best-fit model identifies 5 predictors of language endangerment, none which relate to climate or biophysical landscape properties. Therefore I am uncertain why the authors feel projecting based on future climate is appropriate. The authors do acknowledge that there is “no globally consistent association between landscapes or climate and endangerment”, but say that this may be regionally important. I feel that this portion of the analysis should either be removed or needs to be better justified and discussed.

One of the key results was the connection between road density and language endangerment. This is very interesting, but I felt there was a lack of mechanistic explanation to establish the connection. The authors highlight that this response was independent of variables (e.g. GDP), but I felt this required more justification for why increasing road density was such a strong predictor.

A smaller comment, but the authors state that “language diversity is under even greater threat than biodiversity” in multiple places. I understand that they are trying to clarify the urgency and magnitude of this issue and I do find it helpful to put into the context of other threats. However, this statement is only supported by a single reference, the IUCN Red List, and is based solely on threat status for terrestrial vertebrates, which constitute only a small portion of the total biodiversity. The magnitude of biodiversity loss and threat is an extremely active area of research, so I caution against making this particular comparison. I suggest rephrasing to make a qualitative statement putting linguistic diversity loss in the context of biodiversity.

Reviewer #2 (Remarks to the Author):

The article is thought-provoking regarding factors that cause language endangerment and loss (LEL) and can be applied to various situations to account for similarities and differences in outcomes.

However, I think the authors can do a better job of demonstrating their positions. I suggest they start with a cogent review of the factors the current scholarship has proposed as accounting for the processes and what they agree or disagree about. The present discussion is not so clearly articulated! The authors should then move on to explain why the factors they propose as accounting for LEL are (more) explanatory than in the state of the art. While I agree that the current scholarship has done a poor job, I have several reservations about what the authors are proposing, perhaps because the real explanations and examples illustrating their alternative factors are not clearly provided.

It's not clear to me why connectivity by roads or waterways is more significant than the actual population contacts that sometimes trigger competition between languages, favoring some at the expense of others. And, to begin with, why do the authors present connectivity as mutually exclusive with contact? I would say that connectivity leads to contact! Of course contact alone does not explain things, because there are numerous cases of contact that have not produced LEL. This means that population/ socioeconomic structure and political structure are more critical factors than connectivity, which is only indirectly related to the actuation of LEL.

A similar issue arises with islands. They are instances of isolation, which can occur also in mainland populations that are segregated topographically (which the authors acknowledge) and/or socially (as in urban centers). Connections with islands, by bridges or waterways, does not necessarily lead to LEL! It is the ensuing population/socioeconomic structure that may affect the vitality of a language--either that of the indigenous population or of the migrant population. The discussion in the article is so abstract that one cannot learn significantly from the discussion, especially a reader not familiar already with the literature on LEL. And things boil down again to dynamics of social contacts/ interactions within a particular population structure--whether there is power asymmetry between the parties. Which party makes concessions to which?

Urbanization raises similar issues. Different ethnolinguistic groups may live in the same urban center without one affecting negatively the vitality of the language(s) of the other group(s) if they are segregated and can operate economically and politically in their respective languages. Although an urban center is a contact ecology, it is the particular socioeconomic and political structure of the latter that may or may not cause LEL.

As should be obvious to the authors, who sometimes cite Africa, formal education alone will not endanger languages that do not serve as media of education. This is one respect in which the difference between settlement and exploitation colonization becomes so significant. Formal education operates within a specific population structure, which varies from one polity to another, depending largely on whether or not the language of the economy is also the medium of education and whether the whole economy (formal and informal/vernacular; and the white- and blue-collar sectors) operates in the same language. Also, highly educated people in Black Africa have generally not given up their heritage languages. Although their children acquire European colonial languages as their mother tongues, often one of two mother tongues,there are really no signs of indigenous languages being endangered by the European languages in Africa in general.

There should be an adequate explanation for why few indigenous African languages have been endangered or driven to extinction--and in these cases it is other indigenous languages, and not necessarily by major ones, that displace them; not European languages (except with some Khoisan languages in Southern Africa).

Climatic change is only a potential indirect cause of LEL. After all, populations can respond to climatic change in different ways, some of which may not affect their population structures, especially if they do not relocate.

I can go on and on with most of the other factors. I get the impression that the authors have just been speculating about a variety of factors--some of which affect natural ecosystems--that may also affect the vitality of languages. However, they should really take some time to think their conjectures through. A factor such as "human footprint" applies more easily or clearly to natural ecosystems than they do to languages and cultures, which humans themselves develop.

I very much agree with the authors that the demographic size of an ethnolinguistic group is not always a factor that explains LEL. If this is true, then they should think about whether the current predictions about how many languages will be extinct within the next century are accurate. In this regard, they should consider the present pattern of language distribution but also the very long history that has produced the current distribution of languages. Then they can speculate whether the world is likely to evolve in the same trend everywhere.

I expected the supplementary essay to provide more information that respond to my comments above than the technical information about how the predictions have been calculated. The reader who expects modeling to help research make progress in explaining the subject matter will be better served otherwise, for instance in showing that current explanations are inadequate and why. And of course modeling serves better when it empirically grounded rather than when it is based on abstract speculations that may or many not be verified later, empirically. I would love to read a revised version of the article.

Salikoko S. Mufwene

Reviewer #3 (Remarks to the Author):

This was an interesting paper which uses a combination of linguistic and social information to study language endangerment, especially the factors that are most important in leading to community language loss.

While there are many insights to be gained from this paper, substantial further clarification is needed before publication. I have both general and specific comments about the work.

First, I wanted a lot more information about the input to the model and the coded variables; there wasn't enough in the main paper to be able to evaluate the key findings (of course, much more information is in the supplementary materials, but I felt that key aspects of the analysis had to be taken on trust). Given that manyof these variables are usually only available at the country level, but language endangerment is associated with severe disparities within countries and regions, we need to know more about the granularity of the coded variables. Secondly, these are "associations", not causal models, so more should be done to be specific about the causation mechanisms assumed in the paper. Thirdly, there are some aspects of time-lag in data that need more discussion. For example, the presence of sleeping languages in the dataset - today's sleeping languages were "endangered" 50-100 years ago. But the issues around endangerment were different then (for example, the presence of

residential schools in the USA and Canada; the last one closed in 1996 but they were active from the early 1900s). Finally, I found myself constantly wanting to know more about the regional dynamics. There is some discussion about differences at the continent level but even that is very coarse-grained (compare the US southwest vs mid-Atlantic states, for example). finally, I would like to see some discussion of the effects of language endangerment and loss on communities (e.g. around the language and wellbeing literature) and the effects of papers which treat language loss as inexorable on language community projects. Barbra Meek, Bernard Perley, Wes Leonard and Jenny Davis have written on these issues, for example.

Specific comments follow:

Abstract: why those recommendations in particular? could recommendations also include support for linguistic communities?

Does this paper include data on Sign Languages? please mention explicity why they were excluded if not.

p2: the paper talks about predictions of future endangerment, but the paper could alo direct policy and inventions to prevent further language loss and to support communities in key areas.
p4: please provide more information about the effects of including languages which are no longer spoken (ie already so endangered they have lost all speakers) on model estimates.
"surroundings of Himalayas" - rephrase? not sure precisely what this phrase refers to
Drivers of endangerment. I was confused about the inclusion of threatened languages as a "driver" of endangerment. That appears to be circular - that a predictor of language endangerment is that the language is endangered?
Number of speakers: to which extent is this finding driven by a combination of the largest languages being orders of magnitude larger than most of the rest of the world's languages, and many highly endangered languages having single-digit or very small numbers of speakers/signers? Is population size still signitifcant for languages in the 100,000-100,000,000 speaker range, for example?

p5 how is language contact defined here? what is the mechnanism assumed by which lots of languages/linguistic ecology helps or hinders language endangerment?

I didn't understand the comments about barriers to human movement not being a factor, but roads are a factor. Surely roads are implicated in barriers (or otherwise) to human movement.

p6 I appreciate the comments around different types of bilingual education programs and how they play different roles in different regions. This point could be made more of (particularly with respect to policy and prevention of language loss).

p7 Africa and hunter-gatherer groups: My impression (from Binford's work on hunter-gatherers, for example) is that hg-groups are not very numerous in Africa compared to different types of pastoralism, so it's unclear to me how much subsistenceshift might be driving language shift here. A comparison with Amazonia would be in order.

Temperature gradient an Europe: is it climate change and the Arctic, or that the most northerly parts of Europe are where most of the recognized endangered languages are? Here clarification about whether regional languages of Europe are included would be helpful.

p8 - genocide and disease hasn't been mentioned; are these variables also included? they are, for example, a big historical driver of endangerment and language loss in the US, Canada, and Australia. This is another area where I wonder if historical processes are not being sufficiently accounted for (e.g. the number of Australian languages whose speaker numbers are already in single digits, per the National Indigenous Languages Report III).

p10 - some discussion of community agency and approaches would be good. There are many communities working on linguistic rights, activism around language, and language reclamation.

p13 - what granularity of data is available for the predictor variables? is it country level or more specific? Ideally it would be associated with the language/community level.

This brings up multilingualism - the authors discuss "language contact" but this could cover a host of different dynamics that facilitate or impede language transmission and retention.

p15 - please say more about the expectations around language relationship and independence. This is a problem for many typological studies but it's unclear to me why (phylogenetic) language relationship should be a factor in language endangerment.

p26 - this table makes it clearer that life expectancy, education, and schooling is at the national level. This is extremely problematic for countries where endangered languages are both a small minority and are concentrated in particular regions. For example, Native Americans make up 2% of the US population and have an average life expectancy which is 5 years less than the US average.

Averaging over 10,000km² for 'neighborhood' seems problematic; other papers use an average neighbor window (e.g. average over the area of the nearest n neighbors within a minimal or maximal radius, which better captures the combined effects of language density and actual language contact.

Re proportion of languages expected to become "sleeping" over the next 40 years. This is problematic for the Australian case, where none of the languages in the southeastern have first language populations. They were all already sleeping, and the languages which now have speakers have come about through language reclamation programs. That seems to be an important dynamic that's not captured here.

OVerall summary:

- Validity: See comments above. Some aspects of the study need to be clarified and further tested.
- Originality and significance: Overall I think the results of this paper are original and clarify a topic that is relevant to many across the world. However, I think the authors should make the policy implications of the paper clearer, since their results are likely to have a direct impact on the lives of people who are already structurally disadvantaged.
- Data & methodology: See above for my comments on this aspect of the paper. Substantial additional clarifications are required.
- Appropriate use of statistics and treatment of uncertainties: Some comments were made above, particularly regarding the causal inference and methods.
- Conclusions: Do you find that the conclusions and data interpretation are robust, valid and reliable? See above for concerns about global vs regional issues, about issues around causation, and about the

effects of historical (rather than -- or as well as -- contemporary issues).
- Suggested improvements: Please list additional experiments or data that could help strengthening the work in a revision. This relies on the answers to questions above. I don't think additional tests are necessary if the above points can be clarified appropriately.
- References: Does this manuscript reference previous literature appropriately? If not, what references should be included or excluded? yes. I note that the reference list does not have Indigenous writers on language endangerment, however, and that perspective should be included.
- Clarity and context: Is the abstract clear, accessible? Are abstract, introduction and conclusions appropriate? Yes, though with the proviso that more information about methods needs to be in the main paper.

Reviewer #4 (Remarks to the Author):

This paper is a thorough and comprehensive work investigating the potential drivers of language loss and decline, as well as predictors for the future. A phenomenal amount of work has gone into gathering the data to conduct this analysis, which also seems thorough and robust. Overall, I think this is a fantastic piece of work that seems well deserving of publication; I have only minor comments regarding the first half of the paper (understanding current drivers of language endangerment), but have some more substantial concerns about the second half which makes future predictions.

I am a macroecologist, not a linguist (though linguistics is something I have long been interested it), and so review this paper from that standpoint, and cannot make expert comment on the linguistic aspects of the paper.

Future Predictions
I begin with my primary concern: the future predictions aspect of the paper. I very much understand the desire to make future predictions, it seems a logical extension of the "current drivers" model, and is important and interesting to understand, but I'm concerned that the lack of available data on predictors significantly impedes your ability to make predictions. You find the main drivers of language endangerment to be number of L1 speakers, bordering language richness, threatened languages, road density and years of schooling. However, save number of L1 speakers, you are not able to obtain predictions for any of these key variables, only for climate and land use, which (as shown in Figure S10), were not strong predictors for the majority of best-fit models at the global scale. Where they are relevant at the regional scale (e.g. Temperature seasonality in Europe), I can see an argument for making a regional predictive model, but certainly not upscaling to global. I understand that you've chosen these predictors based on available data, but to me it seems that this step of this analysis simply may not be possible.

Even if temperature and human footprint were more important in the model, I also have concerns about using them as predictors. First, with temperature (though as a non-linguist I readily admit the following may be wrong!), I would want a more cohesive hypothesis for how temperature seasonality affects language endangerment. To me, temperature seasonality in Europe is merely a correlate with past patterns of human settlement, and I can't really see a mechanism by which increased seasonality would have a causative role in increasing language endangerment. I could of course me missing something, but if so (and if you decided to continue using this variable despite other concerns), this needs to be more clearly explained. Whilst on this point, language around correlation and causation needs to be watched in the main paper, for instance, there is a sentence where you say "Climate has

the strongest influence in Europe, with endangerment levels increasing with temperature seasonality" – but I would argue that temperature is a correlate, rather than an influence per se.

Secondly, with human footprint, I do not agree with extrapolating the values found by Venter (2016). Assuming that the rate of change in human impact on the environment, and population density, will follow a linear trend from the changes observed from ~1990-~2009 up until 2100 does not seem a reasonable assumption to me, given the amount of unknowns about how the earth and human society could change in that time. What's more, there do exists predictions of land use change over this future time period that would be more suited to the task. I am not familiar with this area of research, but the PREDICTS team I think have done some work that could point you in the right direction (https://doi.org/10.1038/nature14324), again, if you decided to pursue using this variable.

Current drivers of endangerment
Overall the analysis seems extremely rigorous and the statistics and model selection sound (the assessment and resolution of the parallel trends assumption was especially neat). The only issue that jumps out to me is to do with choosing threshold values (Such as the 10,000km2 circle for various predictor calculations, definitions of island languages, and using 1/3 as the cut off for identifying retained predictors). There's not much justification provided for why these values were chosen. Did you sensitivity test them at all?

Figures
Figure 1: This figure is, in my opinion, apologies, a bit of a mess! I can see what you are getting at, but this doesn't seem the best way to display the information. I would rather see a table with a column for each predictor group, a column for the variables within each group, a column for the scale they were recorded at (maybe by "Language", "Neighbourhood", "National" or "Polygon") and a column for significant predictors. Or, if you do keep the figure, I think it needs to be tidied up somehow.

Figure 2: I don't think the lines linking regions to the map are necessary. More importantly, I would consider finding a way to group variables into your wider variable groups, you've done this by colour somewhat, but it would be good to group the legend more (e.g. with square brackets indicating the different group classes, and more similar colours within each group class – for the main text I don't think it's essential to see the difference between, say, bordering language richness and bordering language richness per km, this could be saved for supplementary). Ideally, I think it would be better to amalgamate the colours to just have one value per wider group, but I can't really see how you would do this.

Figure 3: For A could you convert the legend back to normal (rather than log) scale? (i.e. keep the colours logged, but label with actual values)

Figure 4: There is unnecessary use of colour here – consider reducing.

Minor Comments
(I am unsure if this is an error in the PDF, but the file I have received did not contain any line numbers. I'll do my best to signpost to the relevant sections.)

At least for the main text, I would do away with L1 and just refer to "First Language Speakers" or similar – the acronym isn't used enough for readers to remember it.

Is the use of ISO codes in the main text necessary? They are use intermittently in the supplementary. Many of your readers will not be linguists by training and (like me!) may not at first know what they mean. If they do need to be kept, explain what they mean the first time they're introduced (which you do in the methods, but not the main text).

You use the EGIDS acronym in figure captions but not the main text, this needs to be introduced somewhere in main text, or explained in figure (preferable)

I think the discussion should at least make mention of the conflicting issues here of welfare and language endangerment. You've done a good job of it with education, but re roads, I think just a little more discussion about the potential for roads to provide positive social benefits (I think, this is not my area of expertise) and how this can be squared with protecting languages would be good.

It would be good to specify the actual number of languages when you say "90% of the world's languages" in the second paragraph of the intro.

I think the main text just needs a tiny bit more explanation of the methods, e.g. one sentence at the beginning of the "Drivers of Language Endangerment" section briefly explaining the models and process.

In the last paragraph before "Predicting Future language loss" you're missing the word 'is': "For example, in Africa, language endangerment *is* associated with…"

The first sentence under "Predicting Future Language Loss" is a little clunky. In the next sentence, it would be good to just have one more sentence explaining intergenerational transmission before referring to methods.

Last sentence, second paragraph under "Safeguarding language diversity" – I can't really see anywhere where east Siberia is threatened in 3b?

Methods – "Language Data" – I think you should give a brief mention of the datasets used to get language data here.

Generally speaking, your methods are beautifully explained!

Hannah Wauchope

Author Rebuttal to Initial comments

21st June 2021

Reviewer expertise:

Reviewer #1: macroecological methods

Reviewer #2: endangered languages

Reviewer #3: endangered languages

Reviewer #4: macroecological methods

Reviewers' comments:

Reviewer #1 (Remarks to the Author):

found the paper quite interesting and well-written. The authors make a strong, clear case for the importance of understanding language endangerment. As an outsider to the linguistic literature, I am unable to evaluate many of the domain-specific portions of the analysis. Overall, I felt the analysis presented was very comprehensive and potentially very useful for elucidating drivers of linguistic loss.

As a general comment, I am a bit confused by the future projection section. First, the authors did not make the conceptual link between climate and linguistic diversity clear, therefore it was difficult to follow why we might expect future linguistic loss to be driven by changes in climate.

> 1.1 To identify correlates of language endangerment, we included the climatic variables in our model that have previously been shown to be correlates of language diversity (see page 14), and referenced a study that examines these issues in greater detail. We have

now added a statement to this effect on page 3: “We also include variables that have been associated with language diversity, including features of climate and landscape.”

We have expanded the discussion of the role of climate in language diversity in the supplementary information and added references to studies discussing the impact of climate change on language vitality (page S18-19) as follows: “It is important to include environmental variables in an analysis of global language endangerment for several reasons. Firstly, environmental variables, particularly mean growing season and temperature seasonality, have been shown to be significant predictors of language diversity, so including them helps to control for the confounding effect of language diversity on endangerment patterns. Secondly, the association between biodiversity and language diversity can be accounted for by including environmental variables, so once we have these climate variables in the model we do not need to include species richness. Thirdly, there can be an intimate connection between language and environment, and language loss is likely to result in loss of specialist knowledge about natural environments, so we should include variables that represent climate and landscape as part of our explanation of language diversity and endangerment. Fourthly, the environment is undergoing rapid change due to human-induced climate change, so it is essential that we identify any associations between climate variables and language endangerment so that we can predict possible future pressures on language vitality brought about by climate change”

Second, in the first section regarding drivers of language endangerment the best-fit model identifies 5 predictors of language endangerment, none which relate to climate or biophysical landscape properties. Therefore I am uncertain why the authors feel projecting based on future climate is appropriate. The authors do acknowledge that there is “no globally consistent association between landscapes or climate and endangerment”, but say that this may be regionally important. I feel that this portion of the analysis should either be removed or needs to be better justified and discussed.

1.2 We did report the region-specific climate factors that are significant predictors of language endangerment (pages 7 & 20, Figure 1, Supplementary Data S3), but we apologise if we did not make this point clear enough. We have now explained this more clearly.

We have also rerun the forward prediction without climate variables. We report the results of the demographic-only predictions in the main text, but we include the climate and land-use based predictions in the Supplementary Information. We explain in the Supplementary Information that the regionally-significant climate and land-use variables will only influence forward projections within the regions in which they are significant predictors (page S30).

One of the key results was the connection between road density and language endangerment. This is very interesting, but I felt there was a lack of mechanistic explanation to establish the connection. The authors highlight that this response was independent of variables (e.g. GDP), but I felt this required more justification for why increasing road density was such a strong predictor.

1.3 We discuss this point on page 5 but there is relatively little existing literature on this topic, and no quantitative analysis that we are aware of. We have added citations to papers that state that roads are a factor in language vitality (such as Aikenvald (2004) on languages in the Sepik area of New Guinea) but while each of these studies speculates on the role of roads in language endangerment, they do not directly testing for such an effect. As far as we know, ours is the first study to formally investigate the association between roads and language endangerment on a global scale. Therefore, we think it is important that we highlight this result in the hope that it will stimulate further research. But it is important that we do not over-interpret the result – we can show that roads are a significant predictor of global patterns of language endangerment, above and beyond other indicators of economic development or human movement, but we do not have finer level of detail to allow us to investigate the mechanism underlying this observation. We provide a potential explanation on page 5-6 but we are careful not to overstep the bounds of our results.

A smaller comment, but the authors state that “language diversity is under even greater threat than biodiversity” in multiple places. I understand that they are trying to clarify the urgency and magnitude of this issue and I do find it helpful to put into the context of other threats. However, this statement is only supported by a single reference, the IUCN Red List, and is based solely on threat status for terrestrial vertebrates, which constitute only a small portion of the total biodiversity. The magnitude of biodiversity loss and threat is an extremely active area of research, so I caution against making this particular comparison. I suggest rephrasing to make a qualitative statement putting linguistic diversity loss in the context of biodiversity.

1.4 We have changed this comment to remove the relative comparison (so that it now reads “As for global biodiversity, the world’s language diversity is under threat”), and also added specific information on endangerment rates in some biological taxa for comparison (amphibians, birds, mammals, conifers).

Reviewer #2 (Remarks to the Author):

The article is thought-provoking regarding factors that cause language endangerment and loss (LEL) and can be applied to various situations to account for similarities and differences in outcomes. However, I think the authors can do a better job of demonstrating their positions. I suggest they start with a cogent review of the factors the current scholarship has proposed as accounting for the processes and what they agree or disagree about. The present discussion is not so clearly articulated!

2.1 We agree wholeheartedly with Prof. Mufwene that the current ms does not provide a comprehensive review of factors influencing language endangerment and loss. We cannot provide an overview of this rich field within the space requirements of this ms, particularly given the limit on the number of references. So instead we have referenced key reviews and book-length treatments of the field of language endangerment and loss so that readers can access that literature.

The authors should then move on to explain why the factors they propose as accounting for LEL are (more) explanatory than in the state of the art. While I agree that the current scholarship has done a poor job, I have several reservations about what the authors are proposing, perhaps because the real explanations and examples illustrating their alternative factors are not clearly provided.

2.2 We do not make the claim that the factors we include are more explanatory than others, simply that these are factors that we have appropriate data to include in a global quantitative analysis – we now emphasize this point by adding to the introduction on page 2 that we “compare the predictive power of variables representing some of the potential drivers of language loss.”

In addition to the explanations given in the extensive supplementary information on the selection of variables, we now have added a statement to the introduction emphasizing

that there are many important factors that cannot be included in a global quantitative study of this nature due to the lack of available data with world-wide coverage. We have added the following statement to page 3: “Clearly any such list will be incomplete, and the requirement for globally consistent data will fail to capture significant influences on language vitality that operate on regional or local levels. Our aim is not to provide a comprehensive picture of language endangerment but a useful exploration of the influence of a selection of potential impacts”.

We have also added this statement to the discussion on pages 4-5 “These variables cannot provide a full picture of the processes threatening language diversity, as there will be many other important factors that cannot be included due to lack of appropriate and consistent data with global coverage, or because of the idiosyncratic nature of processes of language endangerment and the influence of historical factors cannot be captured in a broad-scale model. By identifying factors that are significant predictors of language endangerment at a global scale, we add to understanding of the complex interaction of factors contributing to language endangerment.”

It's not clear to me why connectivity by roads or waterways is more significant than the actual population contacts that sometimes trigger competition between languages, favoring some at the expense of others. And, to begin with, why do the authors present connectivity as mutually exclusive with contact? I would say that connectivity leads to contact! Of course contact alone does not explain things, because there are numerous cases of contact that have not produced LEL. This means that population/ socioeconomic structure and political structure are more critical factors than connectivity, which is only indirectly related to the actuation of LEL.

2.3 We do not consider connectivity and contact to be mutually exclusive, in fact we discussed on page 5 how connectivity brings languages into contact and can aid the spread of *lingua francas*. Perhaps this impression arose from the results of the analysis, that the direct measure of contact (languages whose distributions overlap or meet at the edges) is a negative predictor of endangerment, while road density is a positive predictor of endangerment. We interpret these results as implying that the association between road density and language endangerment cannot simply explained by roads bringing languages into contact, because measures of contact itself do not predict greater endangerment. We have now modified our discussion on page 5 which hopefully will clarify this point, emphasizing that contact can lead to multilingualism rather than language loss.

A similar issue arises with islands. They are instances of isolation, which can occur also in mainland populations that are segregated topographically (which the authors acknowledge) and/or socially (as in urban centers). Connections with islands, by bridges or waterways, does not necessarily lead to LEL! It is the ensuing population/socioeconomic structure that may affect the vitality of a language--either that of the Indigenous population or of the migrant population. The discussion in the article is so abstract that one cannot learn significantly from the discussion, especially a reader not familiar already with the literature on LEL. And things boil down again to dynamics of social contacts/ interactions within a particular population structure--whether there is power asymmetry between the parties. Which party makes concessions to which?

2.4 We agree entirely that island status does not provide a measure of contact – some islands have many languages in contact, some islands have ongoing contact with neighbouring mainland or other islands, and even distant islands may have regular contact (as seen in the degree of borrowings between languages in many oceanic islands). We include island status in our analysis not as an indicator of isolation, but because island status has previously been suggested to be associated with greater endangerment, as discussed in the Supplementary Information on page S12.

We suggest that the lack of evidence of an influence of island status or geographic barriers to movement on endangerment indicates that contact alone does not explain language endangerment. We have added this statement to page 6: "We can therefore conclude that being in regular contact with speakers of another language does not itself endanger Indigenous language vitality, instead there are other more complex social, economic and political dynamics influencing language endangerment, that may co-occur with language contact but are not synonymous with it."

We have also expanded our discussion of island definitions in the Supplementary Information pages S10-S11, adding the following text:

"Island languages have previously been reported as having higher levels of language endangerment just as island species are often more vulnerable to extinction (Tershey et al). Therefore, we wish to ask if languages on islands have greater levels of endangerment, above and beyond covariation with other predictors, and after correcting for relatedness and spatial non-independence. Since there is a continuous range of landmasses of difference sizes, there is no absolute definition of what constitutes an "island" (for example, should Tasmania be considered an island for the purposes of

language endangerment? If so, then should Sri Lanka be considered an island, because it is approximately the same size? And so on). Therefore, identifying island languages requires us to specify a cut-off land area, which must necessarily draw an arbitrary line between landmasses of different sizes".

Urbanization raises similar issues. Different ethnolinguistic groups may live in the same urban center without one affecting negatively the vitality of the language(s) of the other group(s) if they are segregated and can operate economically and politically in their respective languages. Although an urban center is a contact ecology, it is the particular socioeconomic and political structure of the latter that may or may not cause LEL.

2.5 We agree, and we do not equate urbanization with language contact at any point in the ms. Instead urbanization is taken "as a measure of socioeconomic shift from rural to urban populations" (SI page S16) and as a reflection of "the impact of changing landscape and environment" (page S15). To reflect these two key aspects of urbanization – land use change and socioeconomic change - we have two variables representing urbanisation, one of which is grouped with the "shift" variables, one of which under "land use", neither is categorised as indicating connectivity, contact or language diversity (Table 1, Figure S3).

As should be obvious to the authors, who sometimes cite Africa, formal education alone will not endanger languages that do not serve as media of education. This is one respect in which the difference between settlement and exploitation colonization becomes so significant. Formal education operates within a specific population structure, which varies from one polity to another, depending largely on whether or not the language of the economy is also the medium of education and whether the whole economy (formal and informal/vernacular; and the white- and blue-collar sectors) operates in the same language. Also, highly educated people in Black Africa have generally not given up their heritage languages. Although their children acquire European colonial languages as their mother tongues, often one of two mother tongues,there are really no signs of indigenous languages being endangered by the European languages in Africa in general.

2.6 We agree that it is very difficult to characterize the impact of education at the global level, and we discuss on page 6-7 how even when there is a national policy on minority language use in education, it may have quite variable effects in different regions (we use China and the US as examples to illustrate that point because they are well documented, but we would also be happy to include African examples if Prof. Mufwene

can recommend published studies on this topic). We note that the association between years of schooling and language endangerment has been detected in smaller scale studies (see, for example, ref 49), but we suggest that this requires much more research attention. We say on page 7: “More fine-grained analysis at regional level is needed to examine the influence on vitality of minority languages in classrooms.” Of course, it would be ideal to discuss this in greater detail but the word length limits on the ms make this difficult. We would be happy to take suggestions for key studies we should cite on this topic.

There should be an adequate explanation for why few indigenous African languages have been endangered or driven to extinction--and in these cases it is other indigenous languages, and not necessarily by major ones, that displace them; not European languages (except with some Khoisan languages in Southern Africa).

2.7 Thank you for this interesting point, it has prompted us to include a new figure in the Supplementary Information (Figure S5) that sets out the number of endangered and Sleeping languages in regions across the world, so that readers can compare number of endangered languages not only between Africa and the rest of the world, but also between different regions of Africa.

We mention on page 8 that the situation in Africa is in many ways quite different to other regions, with some groups adopting a neighbouring language. Again, a more targeted regional analysis and a fuller discussion of this point would be ideal, but to do that topic justice would require an independent study focusing on patterns and variables relevant to the Africa situation, which is not possible within a study aiming to use data available for global languages. However, we do reference a number of studies of language endangerment in Africa where readers can go for more detail, and we have now added more citations to studies by African linguists (e.g. Ndhlovu 2020, Essegbey et al 2015, Monaka & Chebanne 2019).

We have added a statement to page 8 that regional patterns of endangerment require more focused analysis: “These regional patterns are ideal foci for future studies of language endangerment: while the current study is constrained to predictors that are globally relevant and consistently measured for all regions of the world, a targeted study could focus on variables considered important at regional scales, such as land-use and

subsistence in Africa, population density change in Oceania, or climate in Europe and Central & Eastern Asia.”

Climatic change is only a potential indirect cause of LEL. After all, populations can respond to climatic change in different ways, some of which may not affect their population structures, especially if they do not relocate.

2.8 We have checked the phrasing in the ms to make sure that we are not implying a direct causal link, but instead are identifying a statistical association. E.g. on page 8 we say “Climate has the strongest association with language endangerment in Europe, with endangerment levels increasing with temperature seasonality, reflecting patterns of language erosion in Artic regions”. We have also added some studies exploring the link between climate and language vitality to page S19 (see point 1.1 above).

I can go on and on with most of the other factors. I get the impression that the authors have just been speculating about a variety of factors--some of which affect natural ecosystems--that may also affect the vitality of languages. However, they should really take some time to think their conjectures through. A factor such as "human footprint" applies more easily or clearly to natural ecosystems than they do to languages and cultures, which humans themselves develop.

2.9 We have chosen variables to represent key factors that have been identified by linguists as important in LEL (see brief overview on page 3), but we now more clearly explain that not all factors of importance can be included in an analysis of this nature. For example, we would have liked to include information on multiligualism in communities, and the presence of active bilingual education programs, but unfortunately these important aspects of language vitality are simply not available with consistent global coverage. Our analysis should be seen as weighting the relative contributions of a large range of factors that can be tractably included in a global quantitative analysis, rather than an exhaustive search for all possible factors that could impact on language vitality. We hope this point is made more clearly in the revised manuscript.

I very much agree with the authors that the demographic size of an ethnolinguistic group is not always a factor that explains LEL. If this is true, then they should think about whether the current predictions about how many languages will be extinct within the next century are accurate. In this regard, they should consider the present pattern of language distribution but also the very

long history that has produced the current distribution of languages. Then they can speculate whether the world is likely to evolve in the same trend everywhere.

> 2.10 Our forward predictions are conservative in that they only include languages that are already identified as being endangered due to reduced intergenerational transmission. Languages that are currently being stably transmitted to children are not included in our predictions, regardless of their current L1 speaker population size. We emphasize this point now on page 8-9 by adding the following statement: “Our analysis is conservative in that it only considers change in L1 speakers in languages identified as having reduced transmission to younger generations, according to the EGIDS classification: we do not model change in speaker number for languages currently considered to be stably transmitted, even though they may become endangered in future”
>
> Our predictions model the influence of L1 speaker number differently in each region (see Figure 2 and Supplementary Data S3), so we do not make the assumption that the influence of L1 on endangerment is the same all over the world. We also use regionally specific demographic information to model the change in L1 speakers, as described in Supplementary Methods 5.2.1, pages S29-30, and in Supplementary Table S9.

I expected the supplementary essay to provide more information that respond to my comments above than the technical information about how the predictions have been calculated. The reader who expects modeling to help research make progress in explaining the subject matter will be better served otherwise, for instance in showing that current explanations are inadequate and why. And of course modeling serves better when it empirically grounded rather than when it is based on abstract speculations that may or many not be verified later, empirically. I would love to read a revised version of the article.

> 2.11 We would certainly not like any reader to get the impression that we imply any inadequacy in previous studies of language endangerment. We present a global quantitative analysis as an alternative and complementary way to ask questions about LEL, not a superior way. We think that we can highlight important processes using this kind of analysis, but a broad-scale quantitative analyses in no way replaces more targeted studies of linguistic change in particular communities or regions. We now

emphasize that large scale studies are complementary to focused studies more clearly on page 3, adding the following statement: “Broadscale quantitative studies are therefore a complement to more focused qualitative studies on language endangerment and loss.”

Salikoko S. Mufwene

Reviewer #3 (Remarks to the Author):

This was an interesting paper which uses a combination of linguistic and social information to study language endangerment, especially the factors that are most important in leading to community language loss.

While there are many insights to be gained from this paper, substantial further clarification is needed before publication. I have both general and specific comments about the work.

First, I wanted a lot more information about the input to the model and the coded variables; there wasn't enough in the main paper to be able to evaluate the key findings (of course, much more information is in the supplementary materials, but I felt that key aspects of the analysis had to be taken on trust).

3.1 As noted, the full details on all of the data and its sources, and the raw data itself, are provided in the Supplementary Information. Given the space limitation in the main ms, it would be impossible to include this level of detail in the main manuscript document. However, we have now moved the table describing all of the variables and their sources to the main manuscript, replacing the previous figure (which has now moved to the Supplementary Information).

Given that many of these variables are usually only available at the country level, but language endangerment is associated with severe disparities within countries and regions, we need to know more about the granularity of the coded variables.

3.2 We agree that granularity of data is a key issue: we discuss this point on page 6-7 with reference to education variables at country level failing to capture regional level implementation. Granularity is clearly indicated for each variable in Figure S3 and in Table 1 where the scale of each variable is indicated. The grid cell size is given in the maps in Figures 1 & 2.

We have now also added a statement to page 16 to emphasize this point: “The variables we include vary in their degree of spatial resolution. For variables concerning legislation and policy (for example, provision for minority language education), data is typically available only at country level. For some socioeconomic variables, such as life expectancy, there is some regional variation available for larger countries, but most areas only have country level data, so for consistency we use national averages provided by global organisations such as the World Bank and World Health Organization (see Table 1). For environmental variables such as temperature seasonality, we can average values over all the grid cells included in the language distribution area, but for landscape factors influencing human movement, such as mountains and roads, values only within the language area are not fully informative, because it is movement between language areas that we wish to capture. So for these variables, we average over all grid cells in a “neighbourhood” centred on the language distribution. For full details of the spatial resolution of each variable, please see the Supplementary Methods.”

Secondly, these are "associations", not causal models, so more should be done to be specific about the causation mechanisms assumed in the paper.

3.3 We agree (see point 2.8 above), and we are careful to report these identified factors as statistical predictors of language endangerment, and not to attribute causation on the basis of this analysis. We do not assume any causal mechanisms, instead we briefly discuss some possible interpretations. This study is designed to capture variables that have statistically significant associations with language endangerment, but it is beyond the scope of this study to establish causes for those patterns. We hope the results of this

global-scale study will stimulate more focused studies that can examine possible causes in more detail.

Thirdly, there are some aspects of time-lag in data that need more discussion. For example, the presence of sleeping languages in the dataset - today's sleeping languages were "endangered" 50-100 years ago. But the issues around endangerment were different then (for example, the presence of

residential schools in the USA and Canada; the last one closed in 1996 but they were active from the early 1900s).

3.4 The influence of history is highlighted in the manuscript on pages 1, 2, 10, 14, 16, and on S21, but only briefly given the space limitations, so we now address this point more explicitly on page 5. The time lag in language endangerment and loss is a critical point, and that is why we think the study would be incomplete without projecting language loss into the future: a language that today is only spoken by elders must become Sleeping within a few decades unless transmission to younger generations is revitalized.

We have added the following statement to page 5 to emphasize this point: “Furthermore, language endangerment and loss is an ongoing process, and there may be historical factors that caused dramatic reduction in L1 speakers that will not be captured in current values of socioeconomic variables, such as massacres of Indigenous populations or ethnic groups, punishing people for speaking their language and separating children from parents. Because of these unavoidable limitations, no study of this kind can aim to comprehensively describe factors affecting vitality of all of the world’s languages. But by identifying contemporary factors that are significant predictors of current patterns of language endangerment at a global scale, we add to understanding of the complex interaction of factors contributing to language endangerment.”

Finally, I found myself constantly wanting to know more about the regional dynamics. There is some discussion about differences at the continent level but even that is very coarse-grained (compare the US southwest vs mid-Atlantic states, for example).

3.5 We have now added a new figure (Figure S5) which gives the number of endangered and Sleeping languages for regions across the world. Regional variation in language endangerment patterns are presented in figure 3, and all of the broad-scale regional results for predictors of language endangerment are presented in full in graphical format in figure 2, as well as being given in numerical detail in Supplementary Data S3, so readers can assess the degree to which patterns vary between regions in detail for each variable and each region. The factors that vary at a regional level are highlighted in Figure 1. Although we briefly mention some regional results (e.g. page 8-9), space limitations prevent a more detailed analysis of each region, and some of the key variables are not available in sufficient detail at finer spatial scales (see Table 1). This study is specifically designed to focus on the global picture, but we present all of our raw data to allow other researcher to build a more detailed region-specific analysis. Indeed, our research group plans to conduct more focused regional analyses in future, but this will require construction of new datasets, with different variables and finer spatial resolution, so cannot be incorporated into this global study.

finally, I would like to see some discussion of the effects of language endangerment and loss on communities (e.g. around the language and wellbeing literature) and the effects of papers which treat language loss as inexorable on language community projects. Barbra Meek, Bernard Perley, Wes Leonard and Jenny Davis have written on these issues, for example.

3.6 Thank you for this helpful suggestion, we are aware how important language is for community wellbeing but we had not included this perspective due to lack of space. However, we agree that it deserves special emphasis, so we have added a brief comment and a number of key references. For example, on page 12 we have added: “The benefits of providing support to enhance Indigenous language vitality, in terms of wellbeing and socioeconomic outcomes, are likely to far outweigh the costs. Implementation of support for Indigenous language vitality at all levels of governance and within speaker communities is urgent, given the predicted rate of loss of L1 speakers who can aid language vitality and transmission (Figure 3).”

We have also added a reference to Jenny Davis’ work as an example of language revitalisation: “to support communities to re-invigorate intergenerational language transmission”. We are happy to take further advice on appropriate ways to include this critical aspect of language endangerment.

Specific comments follow:

Abstract: why those recommendations in particular? could recommendations also include support for linguistic communities?

> 3.7 We enthusiastically agree with this point! We have added this to the abstract. We use the example of Alutiiq on page 9 to highlight community engagement in language vitality but we have also now added a statement emphasizing the importance of community-level support to the final section of the Discussion on page 14: "Investing in speaker communities to provide them with the support they need to encourage language diversity and vitality will bring measurable benefits in terms of social justice, community wellbeing and cultural engagement".

Does this paper include data on Sign Languages? please mention explicity why they were excluded if not.

> 3.8 As stated on page 13, our analysis includes only spoken languages. There are several reasons for focusing on spoken languages rather than sign languages. Firstly, it allows us to use the Glottolog label "Spoken L1 Languages" to filter the dataset – without this filtering step, the analysis would include a large number of languages that have either never had L1 speakers or have not had L1 speakers in recent times, for example cants (e.g. Polari), historically significant languages that are not currently learned as first languages (e.g Sanskrit) or ritual languages (e.g. Machajuyai-Kallawaya). Secondly, sign languages are challenging to place within the language taxonomy that allows us to correct for relatedness. Thirdly, distribution data for sign languages is often quite sparse and inconsistent, for example many sign languages are spoken by family or social groups embedded within larger language communities. Finally, signed languages are subjected to specific pressures – see page S9 for details. So the choice to exclude sign languages should not be interpreted as indicating lack of importance, but that the endangerment status of sign languages requires a special analysis, and would be poorly served by inclusion in this global analysis.
>
> We have added a more detailed explanation to the Supplementary Information on page S8-9.

p2: the paper talks about predictions of future endangerment, but the paper could alo direct policy and inventions to prevent further language loss and to support communities in key areas.

3.9 We agree that this is an important point, see point 3.7 above. However, the key predictive factors identified in this study do not directly lead to simple interventions – for example, while road density is a correlate of language endangerment it would not be very practical to suggest a halt to road building activities – so any discussion of interventions would not be directly drawn from our own results. We do point out on pages 12-13 that some predictors – such as education – are amenable to manipulation, and we place an emphasis on investment in documentation as a key to language revitalization, but it is beyond the scope of this broadscale study to target specific interventions that will work at local and regional levels.

p4: please provide more information about the effects of including languages which are no longer spoken (ie already so endangered they have lost all speakers) on model estimates.

3.10 Our endangerment scale is continuous – languages that are no longer spoken because they have no living L1 speakers are one extreme end of that continuum. We explicitly test whether the relationship between predictor variables and endangerment is continuous across all levels of endangerment, or whether some levels show distinct patterns – this is the test of parallel assumption of the model. We show that the effects of various variables on shifting the endangerment level of a language are consistent across levels, including the higher level of endangerment (Sleeping languages that are no longer spoken). We have now expanded our explanation of the parallel assumption test on page S26 to make this point clearer.

"surroundings of Himalayas" - rephrase? not sure precisely what this phrase refers to

3.11 we have now rephrased this to "Himalayas and Yunnan"

Drivers of endangerment. I was confused about the inclusion of threatened languages as a "driver" of endangerment. That appears to be circular - that a predictor of language endangerment is that the language is endangered?

3.12: Good point, we have re-labelled this section “Predictors of language endangerment”, and we have also changed the title of the paper to “Global predictors of language endangerment and the future of linguistic diversity”

Number of speakers: to which extent is this finding driven by a combination of the largest languages being orders of magnitude larger than most of the rest of the world's languages, and many highly endangered languages having single-digit or very small numbers of speakers/signers? Is population size still signitifcant for languages in the 100,000-100,000,000 speaker range, for example?

3.13 The analysis explicitly deals with this issue. All predictor variables are transformed to reduce long tails on the distributions (see Table 1) and standardized to have the same range of variance – the standardizations were given in Supplementary Information 4.2, as follows: “All the numeric predictors are centred (subtracted from the mean) and scaled (divided by standard deviation) before analyses, so that the magnitude of their regression coefficients reflects their relative impact on language threat status.”

This means that extreme values of population size will not have undue influence on the relationship between speaker population size and endangerment level. Most languages in this size range will fall into the Stable category, but some endangered languages do have relatively large speaker populations. For example, there are 18 Threatened languages (EGIDS =7) and 61 Shifting (EGIDS=6b) languages with current L1 reported as greater than 100,000, and 13 Shifting languages with reported L1 over 100,000,000 (see Supplementary Data S1)

p5 how is language contact defined here? what is the mechnanism assumed by which lots of languages/linguistic ecology helps or hinders language endangerment?

3.14 We have clarified the concept of language contact on page 5-6, and added a discussion of contact potentially leading to multiligualism rather than language shift.

I didn't understand the comments about barriers to human movement not being a factor, but roads are a factor. Surely roads are implicated in barriers (or otherwise) to human movement.

> 3.15 We have now clarified the discussion on page 6-7 as follows: “One interpretation of the association between road density and language endangerment is that roads increase human movement and so bring people into contact with speakers of other languages, and this contact may result in language shift. However, our results suggest that the association between language endangerment and roads is unlikely to simply reflect language contact. If language contact itself generated language shift and loss, then we would expect languages with a high degree of contact with other languages to be more endangered. In fact, we find the opposite: languages whose distribution overlaps or meets many other languages are less endangered (Figure 2, Supplementary Data S3). Furthermore, if contact between speakers of different languages was a driver of language loss, then we would expect landscapes that inhibit movement to reduce language contact and show lower levels of endangerment, but none of the other connectivity variables, such as altitudinal range, landscape roughness or density of waterways, show consistent association with language endangerment.”

p6 I appreciate the comments around different types of bilingual education programs and how they play different roles in different regions. This point could be made more of (particularly with respect to policy and prevention of language loss).

> 3.16 This is something the authors feel strongly about, so we would love to include a longer discussion of the need for well-supported bilingual education, but in the end our analysis doesn’t not have direct bearing on bilingual education because we do not have the data to test it. Our education variables are necessarily coarse grained, targeting legislative provision and school completion rates, recorded at country level, and this doesn’t allow us to directly test the effect of bilingual education. So while we believe strongly in the advantages of bilingual education, we would be stepping beyond the results of the analysis to make a claim for the effectiveness of bilingual education in reducing language loss rates. We do, however, reference sources that discuss the impact of bilingual education.

p7 Africa and hunter-gatherer groups: My impression (from Binford's work on hunter-gatherers, for example) is that hg-groups are not very numerous in Africa compared to different types of

pastoralism, so it's unclear to me how much subsistenceshift might be driving language shift here. A comparison with Amazonia would be in order.

3.17 We have now clarified this statement that the association with land use may reflect subsistence change, and that one example of such a change in the shift from hunter-gatherer to settled agriculture, of which examples are given in the reference cited. We don't discuss Amazonia in this context simply because our analysis does not identify any environmental or land-use predictors of language endangerment in South America. This doesn't mean that subsistence change is not important in language shift in Amazonia, only that our analysis is not able to address that question directly.

Temperature gradient an Europe: is it climate change and the Arctic, or that the most northerly parts of Europe are where most of the recognized endangered languages are? Here clarification about whether regional languages of Europe are included would be helpful.

3.18 We are unsure what the reviewer means by "regional languages" – we analyse 6511 languages including many minority languages of Europe and the Arctic, but our analysis focusses on languages rather than dialects. The full list of languages is given in Supplementary Data S1, which can be sorted by region. We have also added a new figure with the number of languages in each region for our dataset (Figure S5).

p8 - genocide and disease hasn't been mentioned; are these variables also included? they are, for example, a big historical driver of endangerment and language loss in the US, Canada, and Australia.

3.19 These are very important factors, and we now acknowledge this explicitly in the ms. Unfortunately, we do not have a globally consistent way of including past human conflict in our analysis. But we have now added a statement acknowledging the influence of violent conflict to page 5: "there may be historical factors that caused dramatic reduction in L1 speakers that will not be captured in current values of socioeconomic variables, such as massacres of Indigenous populations or ethnic groups, punishing people for speaking their language and separating children from parents".

There are variables that represent both current and historic parasite stress, but they would be unlikely to address this point for two reasons. Firstly, variables representing parasite stress do not adequately capture specific epidemics that have devastated particular Indigenous populations: for example, the values of parasite stress for Australia would not adequately capture the smallpox epidemic that devastated many Indigenous populations. Secondly, previous studies have already investigated the association between language diversity and infectious disease and found that it is driven by covariation with environment. Current or historical parasite load scales with biodiversity and climate, and has been shown to have no significant explanatory power for language distribution once these confounding factors are taken into account (see ref 70).

We have added a statement to this effect on pages 16-17: “The variables included in this study necessarily represent current environments, socioeconomic status and contemporary policy settings. Aside from the Shift variables (Figure S3, Table 1) which represent change over time, we are unable to directly capture historical processes, such as past educational programs, historical disease epidemics, or incidence of warfare or genocide. These are very important factors in language endangerment but cannot be easily represented in globally consistent, universally available variables, so are beyond the scope of this global analysis of current endangerment patterns.”

This is another area where I wonder if historical processes are not being sufficiently accounted for (e.g. the number of Australian languages whose speaker numbers are already in single digits, per the National Indigenous Languages Report III).

3.20 We are clear in the introduction that we don’t aim to cover all processes that threaten languages, that would be impossible for a global level analysis. Instead we ask whether, on top of the specific factors that influence endangerment and loss of particular languages or in specific regions, there are also additional factors that have predictive power for patterns of endangerment. This point is made on pages 2, 5, 11, 14, 17 in the main text.

p10 - some discussion of community agency and approaches would be good. There are many communities working on linguistic rights, activism around language, and language reclamation.

3.21 See point 3.7 above.

p13 - what granularity of data is available for the predictor variables? is it country level or more specific? Ideally it would be associated with the language/community level.

3

.22 The scale of each variable is set out in Figure S3, Table 1 and in the extensive discussions of data collection for each variable in the Supplementary Information.

This brings up multilingualism - the authors discuss "language contact" but this could cover a host of different dynamics that facilitate or impede language transmission and retention.

3.23 We have now clarified our use of the term “Language contact” on page 6, and we are careful to refer to the variable Bordering languages as representing just one aspect of language contact. We would love to be able to include data on levels of multilingualism, but it is unfortunately available for very few communities (see points 2.3, 2.9 and 3.14 above).

p15 - please say more about the expectations around language relationship and independence. This is a problem for many typological studies but it's unclear to me why (phylogenetic) language relationship should be a factor in language endangerment.

3.24 This point is explained on page 18, where we emphasize that endangerment itself is not assumed to be heritable, but that related languages may share patterns of endangerment. We have added the following clarification to page 18: “All analyses rest on the assumption that datapoints are statistically independent of each other, so if we find that the residuals of the model show phylogenetic signal, then this must be taken into account in order to satisfy the statistical assumptions of the test. Our method estimates the contribution of relatedness to observed patterns of endangerment, so that if there is little or no influence of relatedness on patterns of endangerment, then the phylogeny will have no effect on the outcomes”

p26 - this table makes it clearer that life expectancy,patte education, and schooling is at the national level. This is extremely problematic for countries where endangered languages are both a small minority and are concentrated in particular regions. For example, Native Americans make up 2% of the US population and have an average life expectancy which is 5 years less than the US average.

3.25 We agree absolutely that this data is too coarse-grained to reflect languages use at a local level – this point is discussed on page S31, where we discuss the example of Indigenous Australians having a life expectancy ten years lower than the general population. Unfortunately, finer grained socioeconomic data is not available for most countries. For example, while some countries have data on life expectancy of different regions, or for different ethnic populations, it is not available consistently enough for us to include in a global analysis.

We have added the following statement to page 16: “Socioeconomic context is represented by Gross Domestic Product *per capita* (GDPpc), the Gini index of income inequality, and life expectancy at 60 years (Tables S5, S7), noting that these national averages do not capture variation between groups within each country.”

Averaging over 10,000km² for 'neighborhood' seems problematic; other papers use an average neighbor window (e.g. average over the area of the nearest n neighbors within a minimal or maximal radius, which better captures the combined effects of language density and actual language contact.

3.26 Any selection of area or grid size for data collection will necessarily be arbitrary. Nonetheless for some variables we felt it was important to include areas surrounding language polygons as these are a better representation of the “language ecology” than focusing only on the area within the language distribution. So we had to make a choice about a reasonable size for data collection. This area was chosen to be appropriate to the level of resolution in the environmental data, and to roughly represent a day’s journey on foot from the centre of the area to the edge. It would be less useful to use an average over n neighbours because it would conflate the measurement with language diversity. We clarify this in the Supplementary information on page S6, and we emphasize that this choice is arbitrary. As per point 4.4, we are not able to test sensitivity of parameter choices as the analysis is computationally intensive and rerunning the analysis with alternative values is not practical.

Re proportion of languages expected to become "sleeping" over the next 40 years. This is problematic for the Australian case, where none of the languages in the southeastern have first language populations. They were all already sleeping, and the languages which now have speakers have come about through language reclamation programs. That seems to be an important dynamic that's not captured here.

3.27 We agree that the data for Australia is problematic, as is clear from Figure 3 where the representation over southern Australia is patchy. We are confined to using the data from the published sources as set out in Table 1, as it is beyond the scope of this study to conduct an independent investigation of speaker numbers in each of the 6511 languages in the database, but readers can make their own evaluation based on the raw data in Supplementary Data 1 and the coverage in Figures 2-3 and Figures S5-S9. We have added the following statement to page 15: “Some regions or language families may be poorly represented in global databases (e.g. Australian languages have patchy representation and would benefit from expert revision on speaker numbers and endangerment levels).”

Languages that are reclaiming speakers through revitalization programs are included in EGIDS level 9 (Reawakening) but will be recorded as having an L1 of zero, and are classified as “Sleeping” in our scheme (Table S1). However, we note that while the raw data may underestimate the number of Sleeping languages in southeastern Australia, our model suggests a greater number of Sleeping languages than currently recognized in the database (see Figure S5B). This suggests that our model is able to capture the dynamic in southeastern Australia: languages that currently have speakers recorded in the database in southeastern Australia are predicted to be Sleeping languages by the model.

OVerall summary:

- Validity: See comments above. Some aspects of the study need to be clarified and further tested.

- Originality and significance: Overall I think the results of this paper are original and clarify a topic that is relevant to many across the world. However, I think the authors should make the

policy implications of the paper clearer, since their results are likely to have a direct impact on the lives of people who are already structurally disadvantaged.

3.28 We agree, but we are also wary of stepping beyond the results of our study – we are careful to provide enough discussion to contextualise our results without offering unsupported advice. We hope that these results will be used by researchers and language activists to strengthen interventions at a country, regional or local scale.

- Data & methodology: See above for my comments on this aspect of the paper. Substantial additional clarifications are required.

- Appropriate use of statistics and treatment of uncertainties: Some comments were made above, particularly regarding the causal inference and methods.

- Conclusions: Do you find that the conclusions and data interpretation are robust, valid and reliable? See above for concerns about global vs regional issues, about issues around causation, and about the effects of historical (rather than -- or as well as -- contemporary issues).

- Suggested improvements: Please list additional experiments or data that could help strengthening the work in a revision. This relies on the answers to questions above. I don't think additional tests are necessary if the above points can be clarified appropriately.

- References: Does this manuscript reference previous literature appropriately? If not, what references should be included or excluded? yes. I note that the reference list does not have Indigenous writers on language endangerment, however, and that perspective should be included.

3.29 This is a very good point - we agree that the voice of speakers of endangered languages currently has insufficient emphasis in the academic literature, and that this problem is manifest in our bibliography. We did include some publications with Indigenous authors (e.g. Bromham et al 2020) but we have now added additional references in response to this point (e.g. Taff et all 2020, Kik et al 2021, Cochran et al 2013, Riseth et al 2015, Davis 2016). We agree that this is still not enough, and would be happy to take suggestions on appropriate references to include that would increase the voice of speakers of endangered languages in the bibliography.

- Clarity and context: Is the abstract clear, accessible? Are abstract, introduction and conclusions appropriate? Yes, though with the proviso that more information about methods needs to be in the main paper.

> 3.30 We agree that more detail on methods in the main ms would be preferable but given the word limits on manuscript length and the complexity of the data collection and analysis we have already put as much detail in the main ms as space will allow and the rest is in the extensive supplementary information. We would be happy to take advice from the editor about the possibility of expanding the methods section if that is allowed.

Reviewer #4 (Remarks to the Author):

This paper is a thorough and comprehensive work investigating the potential drivers of language loss and decline, as well as predictors for the future. A phenomenal amount of work has gone into gathering the data to conduct this analysis, which also seems thorough and robust. Overall, I think this is a fantastic piece of work that seems well deserving of publication; I have only minor comments regarding the first half of the paper (understanding current drivers of language endangerment), but have some more substantial concerns about the second half which makes future predictions.

I am a macroecologist, not a linguist (though linguistics is something I have long been interested it), and so review this paper from that standpoint, and cannot make expert comment on the linguistic aspects of the paper.

Future Predictions

I begin with my primary concern: the future predictions aspect of the paper. I very much understand the desire to make future predictions, it seems a logical extension of the "current drivers" model, and is important and interesting to understand, but I'm concerned that the lack of available data on predictors significantly impedes your ability to make predictions. You find the main drivers of language endangerment to be number of L1 speakers, bordering language richness, threatened languages, road density and years of schooling. However, save number of L1 speakers, you are not able to obtain predictions for any of these key variables, only for climate and land use, which (as shown in Figure S10), were not strong predictors for the majority of best-fit models at the global scale. Where they are relevant at the regional scale (e.g.

Temperature seasonality in Europe), I can see an argument for making a regional predictive model, but certainly not upscaling to global. I

understand that you've chosen these predictors based on available data, but to me it seems that this step of this analysis simply may not be possible.

> 4.1 The model accounts for regional differences in predictors, so that the influence of these factors will vary between regions according to the empirically based model, and will not influence language endangerment in regions where they are not identified as predictors. However, to address this criticism we have rerun the forward prediction using only the demographic predictions for the number of L1 speakers over time, and not the climate or land-use projections. We now present just this simpler model in the main paper, but we present the full model in the Supplementary Information along with a discussion of these issues. Figure 4 has been updated to give only the demographic predictions, which are the primary drivers of future language endangerment. The new results are qualitatively the same as the previous predictions.

Even if temperature and human footprint were more important in the model, I also have concerns about using them as predictors. First, with temperature (though as a non-linguist I readily admit the following may be wrong!), I would want a more cohesive hypothesis for how temperature seasonality affects language endangerment. To me, temperature seasonality in Europe is merely a correlate with past patterns of human settlement, and I can't really see a mechanism by which increased seasonality would have a causative role in increasing language endangerment. I could of course me missing something, but if so (and if you decided to continue using this variable despite other concerns), this needs to be more clearly explained. Whilst on this point, language around correlation and causation needs to be watched in the main paper, for instance, there is a sentence where you say "Climate has the strongest influence in Europe, with endangerment levels increasing with temperature

seasonality" – but I would argue that temperature is a correlate, rather than an influence per se.

> 4.2 We agree that climate is a correlate of language endangerment that might not reflect a direct causal role of climate in endangerment, and we have carefully edited the ms to make sure that we do not use causal language in describing these relationships. In fact, this is not specific to climate variables, it is the case for all of our predictors. We aim to identify statistically significant associations between our variables and endangerment, and use those to evaluate the relative support for different proposed causes of endangerment and predict future patterns. Whatever the causal relationship underlying

the statistically significant associations, we can still use that significant association to model a possible outcome as those variables change in future. Climate provides relatively little explanatory power in our model, so its influence on future predictions is limited.

Secondly, with human footprint, I do not agree with extrapolating the values found by Venter (2016). Assuming that the rate of change in human impact on the environment, and population density, will follow a linear trend from the changes observed from ~1990-~2009 up until 2100 does not seem a reasonable assumption to me, given the amount of unknowns about how the earth and human society could change in that time. What's more, there do exists predictions of land use change over this future time period that would be more suited to the task. I am not familiar with this area of research, but the PREDICTS team I think have done some work that could point you in the right direction (https://doi.org/10.1038/nature14324), again, if you decided to pursue using this variable.

4.3 We agree that a linear extrapolation from average rates of change over the past decades is simplistic, and we now emphasize this point on page S34. Our aim is not to provide accurate forecasts of future conditions but to ask more generally "if current rates of change continue, how might this influence language endangerment patterns". This same extrapolation is also applied to the demographic data – we cannot predict future implementations of programs that might increase (or slow the rate of decrease) of speakers, nor can we forecast changes in the future that might accelerate rates of loss of speakers. But we can explore one particular scenario which is to ask what will happen if things continue as they have been doing.

There have been more sophisticated projections of future land use change that account for alternative shared socioeconomic pathways/emissions scenarios (SSPs), and other sources of uncertainty (although they are still all based on extrapolating from recent trends) such as the PREDICTS project (https://doi.org/10.1038/nature14324) and recent paper by Chen et al (https://doi.org/10.1038/s41467-020-14386-x). However, the confidence bounds on their estimates are substantial and overlap among many of the different scenarios, and a simple linear extrapolation approximates a mid-range scenario, (eg SSP2) so we feel that for our purposes this is a reasonable and defensible choice. However, we now present predictions without any future extrapolation of land-use change.

Current drivers of endangerment

Overall the analysis seems extremely rigorous and the statistics and model selection sound (the assessment and resolution of the parallel trends assumption was especially neat). The only issue that jumps out to me is to do with choosing threshold values (Such as the 10,000km2 circle for various predictor calculations, definitions of island languages, and using 1/3 as the cut off for identifying retained predictors). There's not much justification provided for why these values were chosen. Did you sensitivity test them at all?

4.4 In each of these cases, we have to choose a reasonable threshold, and any choice we make will be to some extent arbitrary but we have tried to make sure these choices are reasonable and robust.

For a discussion of the island size see 2.4 above, and the expanded discussion on pages S10-11 of the supplementary information. The 10,000km2 threshold is arbitrary, which we acknowledge in the discussion on page 10, but provides a reasonable cut-off: we give examples of islands just over the size threshold and these are relatively large and diverse landmasses. We note that this is similar to the definition from the Global Island Database (<0.11ha) used by Tershey et al. (2015) analysis of language endangerment patterns. After running the pipeline that assigned each language to island status based on its distribution, we validated the approach by selecting and checking island languages in the database against independent information sources.

For a discussion of neighbourhood size, see 3.26 above. While any such designation must be arbitrary, the size of the neighbour was chosen to provide an appropriate scale given the granularity of the environmental variables, and the area over which people might be expected to come into contact with neighbouring populations (representing roughly a day's journey on foot from the centre of the neighbourhood to the edge, though this of course will depend on landscape and population norms for travel). In addition to defining this based on unaided human movement, we plotted several alternative neighbourhood sizes on the global map to check that this choice gave the most reasonable distributions.

In the equally best-fit models (Fig.S10), slightly more than 1/3 of the models are models without lasso, which are the models that do not account for collinearity. Since variables that are included in the majority of models with lasso are also included in the majority of models without lasso, we chose 1/3 as cut-off, so that we can exclude variables that are only included in models without lasso, while still being able to keep variables that are included in nearly all the models without lasso, even if they are not included in any model with lasso.

It is unfortunately not practical to sensitivity test these thresholds. A single analysis run takes at least a month, so rerunning for several levels of each of these variables would take a prohibitively long time. Since island was not a significant factor in the analysis, the benefit to running with different island sizes would be unlikely to match the cost in terms of computational time.

Figures

Figure 1: This figure is, in my opinion, apologies, a bit of a mess! I can see what you are getting at, but this doesn't seem the best way to display the information. I would rather see a table with a column for each predictor group, a column for the variables within each group, a column for the scale they were recorded at (maybe by "Language", "Neighbourhood", "National" or "Polygon") and a column for significant predictors. Or, if you do keep the figure, I think it needs to be tidied up somehow.

4.4 We provided information in the variables in both visual format and table format because we find that some people find a graphical format easier to digest. On the one hand, the graphical format can very quickly convey not only the variables included but also the scale on which they were measured, and which ones were globally significant or regionally significant predictions. On the other hand, a table requires much more effort to read and understand but can have a greater level of detail. To get the best of both worlds, we have now moved the table S2 from the supplementary information to the main text, and have moved Figure 1 into the Supplementary Information (now Figure S3). We are happy to take advice on which aspects of the figure need to be tidied up, as we are not sure exactly what is required.

Figure 2: I don't think the lines linking regions to the map are necessary. More importantly, I would consider finding a way to group variables into your wider variable groups, you've done this by colour somewhat, but it would be good to group the legend more (e.g. with square brackets indicating the different group classes, and more similar colours within each group class – for the main text I don't think it's essential to see the difference between, say, bordering language richness and bordering language richness per km, this could be saved for supplementary). Ideally, I think it would be better to amalgamate the colours to just have one value per wider group, but I can't really see how you would do this.

4.5 it is challenging to display all of this information on one figure, and we are happy to take advice on ways to improve this figure. But we are not sure given this comment what the appropriate change is. Our use of colour was designed to provide an easy visual link to the variable categories in Figure 1. While we appreciate that some variables might be less interesting – such as including both bordering languages and bordering languages per km – including some but not others would mean that the figure does not accurately display the results of the model, and would involve an arbitrary choice of which aspects of the model should be included and which left out.

Figure 3: For A could you convert the legend back to normal (rather than log) scale? (i.e. keep the colours logged, but label with actual values)

4.6 We have made this change to the legend on figures 3, S8 and S10.

Figure 4: There is unnecessary use of colour here – consider reducing.

4.7 We are not sure whether this comment refers to choice of colours in the figure, or a suggestion that this figure should be black and white not colour. We are happy to take further guidance on optimal colour use in this figure.

Minor Comments

(I am unsure if this is an error in the PDF, but the file I have received did not contain any line numbers. I'll do my best to signpost to the relevant sections.)

At least for the main text, I would do away with L1 and just refer to "First Language Speakers" or similar – the acronym isn't used enough for readers to remember it.

4.8 L1 is standard terminology in linguistics, and is the label given to the variable in cross-linguistic databases. It is used over 30 times in the main text alone, so we don't think it could be easily removed. We have gone through the text and made sure our use of L1 is consistent.

Is the use of ISO codes in the main text necessary? They are use intermittently in the supplementary. Many of your readers will not be linguists by training and (like me!) may not at first know what they mean. If they do need to be kept, explain what they mean the first time they're introduced (which you do in the methods, but not the main text).

4.9 This is a linguistic standard, somewhat akin to using scientific names rather than only common names for species. For example, we use Alutiiq as an example on page 11-12, but this language has been referred to using a variety of names including Pacific Yupik, Pacific Gulf Yupik, Sugpiak, Sugpiaq, Sugcestun, Suk, Supik, Koniag-Chugach, Kodiak Alutiiq and so on – if we provide the ISO code [ems] then it is unambiguous which language we are referring to, and it can be identified in any database.

In addition to the explanation in the Supplementary Information, we have added the following statement to page 14: "We give the standard nomenclature according to the ISO 639-3 three letter language identifiers in Supplementary Data S1, and throughout this document we give the ISO code in square brackets at the first mention of a language."

You use the EGIDS acronym in figure captions but not the main text, this needs to be introduced somewhere in main text, or explained in figure (preferable)

4.10 It was introduced in the methods on p14 – we have now added additional citations and references to the Table S1 where the different scales are explained and compared.

I think the discussion should at least make mention of the conflicting issues here of welfare and language endangerment. You've done a good job of it with education, but re roads, I think just a little more discussion about the potential for roads to provide positive social benefits (I think, this is not my area of expertise) and how this can be squared with protecting languages would be good.

4.11 We agree that there are many complex and conflicting issues surrounding Indigenous health and wellbeing and linguistic and cultural continuity. We now make brief mention of this issue on page 12, with two additional references: "The benefits of implementing Indigenous language vitality programs in terms of wellbeing and socioeconomic benefits are likely to far outweigh any costs. Implementation of support for Indigenous language vitality at all levels of governance and within speaker communities is urgent, given the predicted rate of loss of L1 speakers who can aid language vitality and transmission"

However, we recognize that this is a complex topic beyond the scope of this study. For example, the literature on roads, economic development and cultural continuity is scattered, complex and lacking in empirical analysis: roads clearly connect communities to economic networks that open new opportunities, but also can result in both cultural and linguistic shift. In any case, we also include variables that reflect both economic development (such as GDP) and overall measures of health (such as life expectancy), and these are not identified as significant predictors of language endangerment on this global scale of analysis.

It would be good to specify the actual number of languages when you say "90% of the world's languages" in the second paragraph of the intro.

4.12 Done

I think the main text just needs a tiny bit more explanation of the methods, e.g. one sentence at the beginning of the "Drivers of Language Endangerment" section briefly explaining the models and process.

4.13 The word limit and structure of the main text does not allow extensive explanation of the methods, but we have added the following summary: “Our analysis seeks the best set of variables, from 51 candidate variables, to explain variation in the dependent variable, endangerment level, over and above the covariation due to relationships between languages, spatial auto-correlation and contact between distributions, and allowing for interactions between predictor variables and region. We reduce the number of variables by grouping variables according to their pairwise correlation, then identify independent variables with significant predictive power on a proportion of the data (training dataset), then evaluate the fit of the model on the remaining data (test dataset). We then estimate model parameters on the full dataset.”

In the last paragraph before “Predicting Future language loss” you’re missing the word ‘is’: “For example, in Africa, language endangerment *is* associated with…”

4.14 Fixed, thanks

The first sentence under “Predicting Future Language Loss” is a little clunky. In the next sentence, it would be good to just have one more sentence explaining intergenerational transmission before referring to methods.

4.15 We have rewritten this section as follows: “Some of the predictors of language endangerment identified in our model are likely to change over time. We can therefore project values of these variables forward and use them to model likely future patterns of language endangerment. If a language is no longer being learned by children, then, in the absence of interventions to increase language transmission, we can use demographic information to predict when there will be no more living L1 speakers. We can combine the current L1 speaker population size with endangerment score, which tells us the relative generational distribution of L1 speakers and whether the number of L1 speakers is declining (Table S1), and use demographic information on age structure of the population (Table S8) to predict how many L1 speakers will be alive in future (see Supplementary Methods 5 for details). Our analysis is conservative in that it only considers change in L1 speakers in languages identified as having reduced transmission to younger generations (see Table S1): we do not model change in speaker number for

languages currently considered to be stably transmitted, even though they may become endangered in future. “

Last sentence, second paragraph under “Safeguarding language diversity” – I can’t really see anywhere where east Siberia is threatened in 3b?

4.16 East Siberia is an example of hotspot in Figure 2c (previously labelled Figure 3c), so we have now added 2c to this sentence.

Methods – “Language Data” – I think you should give a brief mention of the datasets used to get language data here.

4.17 These sources are all in Table S2 which has now been moved to the main text, so is now labelled Table 1

Generally speaking, your methods are beautifully explained!

4.18 Thank you Dr Wauchope for this comment! In writing this interdisciplinary study we have worked very hard to make the methods accessible to those for whom they will be unfamiliar, just as we have tried hard to make the linguistic issues clear to readers who are more familiar with studies of biodiversity.

Hannah Wauchope

Decision Letter, first revision:

natureresearch

16th August 2021

*Please ensure you delete the link to your author homepage in this e-mail if you wish to forward it to your co-authors.

Dear Professor Bromham,

Your manuscript entitled "Global predictors of language endangerment and the future of linguistic diversity" has now been seen by the same four reviewers, whose comments are attached. The reviewers have raised a number of concerns which will need to be addressed before we can offer publication in Nature Ecology & Evolution. We will therefore need to see your responses to the criticisms raised and to some editorial concerns, along with a revised manuscript, before we can reach a final decision regarding publication.

While the referees with expertise in macroecological methods feel the manuscript is now acceptable from that angle, the two linguistics referees have remaining comments. We would ask you to pay particular attention to those raised by referee 3, particularly the need to validate country-level data.

We therefore invite you to revise your manuscript taking into account all reviewer and editor comments. Please highlight all changes in the manuscript text file [OPTIONAL: in Microsoft Word format].

We are committed to providing a fair and constructive peer-review process. Do not hesitate to contact us if there are specific requests from the reviewers that you believe are technically impossible or unlikely to yield a meaningful outcome.

When revising your manuscript:

* Include a “Response to reviewers” document detailing, point-by-point, how you addressed each reviewer comment. If no action was taken to address a point, you must provide a compelling argument. This response will be sent back to the reviewers along with the revised manuscript.

* If you have not done so already please begin to revise your manuscript so that it conforms to our Article format instructions at http://www.nature.com/natecolevol/info/final-submission. Refer also to any guidelines provided in this letter.

* Include a revised version of any required reporting checklist. It will be available to referees (and, potentially, statisticians) to aid in their evaluation if the manuscript goes back for peer review. A revised checklist is essential for re-review of the paper.

Please use the link below to submit your revised manuscript and related files:

[REDACTED]

Note: This URL links to your confidential home page and associated information about manuscripts you may have submitted, or that you are reviewing for us. If you wish to forward this email to co-authors, please delete the link to your homepage.

We hope to receive your revised manuscript within four to eight weeks. If you cannot send it within this time, please let us know. We will be happy to consider your revision so long as nothing similar has been accepted for publication at Nature Ecology & Evolution or published elsewhere.

Nature Ecology & Evolution is committed to improving transparency in authorship. As part of our efforts in this direction, we are now requesting that all authors identified as 'corresponding author' on published papers create and link their Open Researcher and Contributor Identifier (ORCID) with their account on the Manuscript Tracking System (MTS), prior to acceptance. ORCID helps the scientific community achieve unambiguous attribution of all scholarly contributions. You can create and link your ORCID from the home page of the MTS by clicking on 'Modify my Springer Nature account'. For more information please visit please visit www.springernature.com/orcid.

Please do not hesitate to contact me if you have any questions or would like to discuss these revisions further.

We look forward to seeing the revised manuscript and thank you for the opportunity to review your work.

[REDACTED]

Reviewer expertise:

as before

Reviewers' comments:

Reviewer #1 (Remarks to the Author):

I appreciate the authors' response to my comments and feel they have sufficiently addressed them.

Reviewer #2 (Remarks to the Author):

I'm generally less satisfied with the revised version of the submission than with the original one, now that I can make better sense of the materials. It all sounds to me like a game of predictions using impressive technology and massive data that are irrelevant to the predicted evolution of language vitality. All evolution is local, driven by local ecological conditions. In the case of languages, the relevant ecological factors involve primarily humans' behavioral responses to the environmental (natural) and socioeconomic pressures on them, though these are influenced by political decisions, imperial/colonial conquests, wars, climate changes, and the topographical positions of where they evolve. These broader ecological factors have no direct bearing on language vitality if the investigators do not factor in how humans react adaptively or maladaptively to them. The population structures in which speakers and signers evolve play a very critical role as they present frames within which speakers/signers respond to ecological pressures. The social human is missing from the submission, making every other consideration too speculative in the absence of discussions of concrete cases from

which the reader is invited to extrapolate.

Making predictions in abstraction based on "global factors" that are predicted by apparently wrong macroecological models inspired by animal biology rather than by virology or epidemiology misses the point. Languages are viruses in depending on the communicative behaviors of their hosts for their survival! Curiously, the authors want to make "global" or general predictions and avoid the complications and idiosyncrasies of specific local or regional cases. However, since the world is not uniform, how can one determine what the "global" factors are without comparing the local and/or regional factors?

It is true that in modeling on can use any number of factors and get correct results, even if the factors are not empirically valid. The question is how useful is the model in such cases when it does not enable the investigators to figure out what is actually going on or suggest to them what are the right questions to ask? If the predictions that the authors make are intended to help speakers interacting with each other in specific multilingual ecologies, within specific political and socioeconomic structures, avoid language loss, why should anyone accept the predictions as accurate if the model is based on factors that are remotely connected to local human behaviors? This is not a game of scoring success in predictions, which I actually think are not convincing if they have not been tested on specific cases, to which they are expected to apply?

We are dealing with predictions intended to influence speakers'/agents' responsive behaviors. What I have read sounds like health care providers making predictions that ignore how people live their lives on a daily basis and focusing on climate change and behavior on islands. Or perhaps is this reminiscent of Milton Friedman recommending an economic development program to Chile without factoring the local factors in it and leading to a disaster?

The inclusion of islands in the "analysis" is actually interesting, because it brings up the possibility--not usually discussed in the relevant literature--that a monolingual population isolated from other populations speaking different languages can lose their language. How? This needs explaining. For instance, I can imagine a natural disaster that causes them to die. However, if the population becomes connected to others by roads (with bridges) or waterways, under what conditions would the population lose their language? For instance, in the USA, the Ocracoke Island, inhabited by a White population, has experienced the endangerment of its brogue. On the other hand, the Gullah-speaking populations on islands on the same coast of South and North Carolina have not experienced the endangerment of Gullah in the same way. In the former case, the typically affluent White in-migrants from the mainland have mixed with the local population. In the latter cases, the White in-migrants have introduced or maintained residential segregation and have not mixed with the local Black islanders, the majority. If Gullah has been endangered at all, it is because of emigration, with the islanders leaving in search of better economic opportunities and finding themselves under pressure to speak differently in order to be competitive in the job market outside their islands. So, connection with the outside world is not necessarily dangerouns. POPULATION STRUCTURE is critical. It helps show that all "contacts" are not the same and do not produce the same effects.

After reading the paper, I cannot tell whether CONTACT is a "global" factor or not, why POPULATION MOVEMENT is apparently less important than LANGUAGE TRANSMISSION when in fact LANGUAGE TRANSMISSION itself is the explanandum (pace the literature on the subject matter) and not the explanans for language loss. The literature is generally descriptive and not explanatory. It's not enough to say that discontinued or decreased language transmission or language shift leads to

language loss. One must explain why these phenomena are happening. Reproducing factors that are often conflicting and not reliable, as the authors also recognize, doesn't help the model do the good job that the authors want and claim it to do. This modeling becomes an ill-conceived one in my view, although the technology is impressive.

I will save myself and the editors time and subordinate my reactions to those of other reviewers if their reactions are positive. I can respond to the published article if invited to, in order to generate more discussion in which other readers may engage. But my opinion on the usefulness of this article and its supplement is clear: A lot of reservations.

Salikoko Mufwene

Reviewer #3 (Remarks to the Author):

Most of the comments to reviewers have been addressed in this version. There are, however, a few points which still need some further discussion and clarification.

pp5-6: Just a comment on the reviewer's remark about urban contact ecologies. Another place where stable multilingualism obtains is where there is both language as a strong marker of ethnic identity and no single dominant group. In such cases, multiple languages seems to be pretty stable. There's anecdotal discussion of this in South Africa, townships, but it's also seen historically in Arnhem Land (Australia). I make this point to agree with the authors in point 2.5

I was surprised by Prof. Mufwene's claim that there is no language loss in Africa, apart from Khoi San. I'm not an Africanist but endangeredlanguages.com (data curated by regional experts) shows plenty of language endangerment on the continent.

I'm not satisfied with the comments on granularity of data. While I'm sympathetic to the authors' predicament -- that some key indicators are only available at the country level -- I'm not satisfied that there's nothing more to say than "this is the best we could do". I would like to see more validation checks to show that the country-level data is not distorting the findings. It's not sufficient to use data which is clearly problematic without some attempt to investigate whether the problematic aspects of the dataset are distorting the results.

Likewise, I think the authors misunderstood my point about the influence of history (their 3.4). My point is about uniformitarian assumptions. They use *current* data to infer endangerment associations, which is not valid for languages whose causes of endangerment directly associated with conditions which no longer apply. This can't be dealt with simply as a caveat, and by projecting language loss into the future. That just expands the uniformitarian problems into the future as well as into the past. It's also not addressed by claiming the model is a global one, since these point affect a substantial percentage of the languages in the sample (since so many languages in North America and Australia are endangered, those two countries alone must account for around a quarter of the data points).

3.8 The rationale for excluding sign languages isn't satisfactory. You could search for "spoken L1 language" + "sign languages", for example (I agree that the exclusion of other languages is justified). Second, I'm not at all convinced about the need for controlling for relatedness when geography is also

included in the model, unless relatedness is some proxy for another factor like morphological complexity. (See more on this below.) Is there any indication that population figures for many spoken languages are a lot more reliable than the sign language data? (In my experience such data are not very accurate for spoken languages either). Many spoken language communities for endangered languages are also embedded in the distribution of other spoken languages. Sign languages, like spoken languages, are affected by pressures around education (wasn't schooling one of the significant factors in the model?). Whether parents use the language or not is presumably also significant for spoken languages. That is, I'm not convinced that the reasons given for excluding sign languages are sufficient.

3.24 Why should related languages share patterns of endangerment that are separate from associations of geography, social position in the group, or other points that also have nothing to do with genetic relatedness?

Reviewer #4 (Remarks to the Author):

Overall, I'm happy with the edits to this paper. The prediction section seems much more reasonable now and I'm generally happy with the responses to my other comments. This is a thorough and important paper and I think the authors have done a great job.

I just have a few final, minor, things:

I think in the abstract it might be better to say something like "We predict that, based on demographics and without intervention, language loss is expected to triple…" rather than the current sentence at lines 14-15. Otherwise it's implied that the predictive model uses the variables from the explanatory model.

At Line 80, specify that the endangerment scale is EGIDS and spell it out. I know that you mention this in the methods, but most people reading the paper won't start with the methods, and given you use EGIDS in figures it's good to explain what it is without someone having to refer to methods.

At L125, you list "threatened languages" as one of the five predictors, again to stop the reader having to refer to elsewhere to understand this sentence, better to say "the number of threatened languages in the vicinity". Linking with this, you then describe this variable as "languages in the region are also endangered" at Line 154 . This inconsistency between threatened and endangered can be confusing, better to change to threatened here to be consistent.

Somewhere in L161-164 it could be good to say "as discussed earlier" just to help any reader that may be confused and think "hasn't this already been mentioned" (clearly I'm down to very minor comments now!)

L200, again to help the reader it would be good to start this paragraph with something like "We didn't find a correlation between education policy and language threat status, this may be because our data on education policy is necessarily coarse grained…"

L250-254. The reader may be left confused as to why you didn't include land-use and climate predictors in the main paper, I wonder whether at the start of the prediction section (e.g. somewhere

in the L226-229 paragraph) you could explain that you were not able to obtain adequate data for most predictors and so instead decided to focus on L1 speakers, and quality at L250 that though you can make predictions with climate and land-use, they were not major contributors to the global model and so are more relevant at a regional scale.

Figures.

Re Figure 1, I have attached a figure demonstrating what I was suggesting by grouping factors, where a square bracket is added to demonstrate which wider group each factor belongs to. It's just to help the reader be able to interpret the figure without needing to refer to Table 1 excessively. If possible, I would recommend reordering the variables so that they are in the same order as given in Table 1. Also, L1 Prop should be grouped with the other Diversity factors, no?

Re Figure 3: By excessive use of colour, I mean there is extra colour in this figure that does not convey extra information, which can be confusing. I would recommend removing all colour from A) (or, alternatively, remove the side bars saying "endangered" and "sleeping" and instead have a legend for blue and red.) and in B), I would remove the red colour on "No L1 Speakers (9-10)" and make this the same grey as "Living L1 speakers (6b-8b)" (red already indicates "little" so is confusing to have it twice). It's also confusing here that in A) the two panels use the same y axis (ranging from 0.1-0.4), while in B) the axis is repeated. Maybe in A) remove the small bit of white space between the upper and lower panels? Finally, I'm not convinced of the utility of the dashed lines in an already busy figure, perhaps it would be better to specify in the caption that the "current" estimates are modelled, but roughly align with actual values?

********************END********************

Author Rebuttal, first revision:

Reviewers' comments for "Global predictors of language endangerment and the future of linguistic diversity"

Reviewer #1 (Remarks to the Author):

I appreciate the authors' response to my comments and feel they have sufficiently addressed them.

Reviewer #2 (Remarks to the Author):

I'm generally less satisfied with the revised version of the submission than with the original one, now that I can make better sense of the materials. It all sounds to me like a game of predictions using impressive technology and massive data that are irrelevant to the predicted evolution of language vitality. All evolution is local, driven by local ecological conditions. In the case of languages, the relevant ecological factors involve primarily humans' behavioral responses to the environmental (natural) and socioeconomic pressures on them, though these are influenced by political decisions, imperial/colonial conquests, wars, climate changes, and the topographical positions of where they evolve. These broader ecological factors have no direct bearing on language vitality if the investigators do not factor in how humans react adaptively or maladaptively to them. The population structures in which speakers and signers evolve play a very critical role as they present frames within which speakers/signers respond to ecological pressures. The social human is missing from the submission, making every other consideration too speculative in the absence of discussions of concrete cases from which the reader is invited to extrapolate.

> *2.1 We agree that it is local forces that determine whether a language persists or is lost. A global analysis of this kind cannot capture the nuance of change "on the ground" for each endangered language. We make this point clearly throughout the manuscript. For example, the second line of the abstract states that "each language is the product of a unique history, subject to specific social, demographic and political pressures". The same point is made on page 3: 'Clearly any list of threatening processes will be incomplete, and the requirement for globally consistent data will fail to capture significant influences on language vitality that operate on regional or local levels" – and on page 12 "Every language represents a unique expression of human culture, and each will be subject to idiosyncratic influences of their specific history and local sociopolitical environment".*
>
> *What this study does is to ask whether, in addition to those specific local factors, we can identify any factors that appear act on many different languages. Finding global or regional patterns does not imply special global mechanisms, it means that there are factors that that occur in the local ecology of many different languages that can be identified as associated with endangerment levels in many different places – for example, national-level language policy might influence many minority languages within the same jurisdiction. While some local processes are idiosyncratic, found in one or few areas, we set out to see if there are any additional factors that are commonly associated with language endangerment at the regional or global level. Our analysis is designed so that, if there were no such general factors, we would not identify any significant predictors. However, we do find that a small number of our variables are significantly associated with language endangerment at regional or global level, beyond the level that we would expect to occur by chance.*
>
> *This approach does not deny the importance of social factors and individual decisions – it asks whether, in addition to those fine scale processes, we can recognise other broader scale factors that are associated with areas of greater relative language endangerment. We cannot say directly whether these 'ecological' factors are causing language endangerment, but our analysis does allow us to say they are significantly associated with levels of endangerment, and this is useful information that may stimulate more detailed investigation.*

Making predictions in abstraction based on "global factors" that are predicted by apparently wrong macroecological models inspired by animal biology rather than by virology or epidemiology misses the point. Languages are viruses in depending on the communicative behaviors of their hosts for their survival! Curiously, the authors want to make "global" or general predictions and avoid the complications and idiosyncrasies of specific local or regional cases. However, since the world is not uniform, how can one determine what the "global" factors are without comparing the local and/or regional factors?

> *2.2 We investigate both global and regional factors, as described in the methods, and shown in Figure 1 – and we find that while some factors are associated with endangerment across all regions, some are specific to particular regions (examples given on page 10, and Figure 1). We are not trying to avoid the*

complications and idiosyncrasies of specific cases, nor do we deny that the level of endangerment of any particular language is the result of its unique local situation and set of circumstances, but we seek general patterns that exist alongside those specific influences on individual languages. Exactly the same is true of the study of endangerment of animal or plant species, which is ultimately driven by local factors and specific threatening processes, just as for languages. Yet global predictive models of species extinction risk have been widely applied in biology because they are often able to reveal broader patterns, shared across many species, that are not easily discernible from single-species studies, such as associations of greater extinction risk with body size, reproductive rate or dietary specialisation. These broad scale predictions in no way replace the "on the ground" study of local factors impacting on species and populations under risk of extinction, nor do they suggest that any variables not included in the study are unimportant (a point we make on pages 3 and 5). We say on page 5: "These variables cannot provide a full picture of the processes threatening language diversity, as there will be many other important factors that cannot be included due to lack of appropriate and consistent data with global coverage, or because of the idiosyncratic nature of processes of language endangerment and the influence of historical factors that cannot be captured in a broad-scale model". We have also altered a statement on page 4 to emphasize that we are studying only a subset of influences: "Here we use global analysis to illuminate some of the complex interaction of extrinsic factors threatening language diversity"

Clearly, languages are not species, but the same analytical tools are often useful for both, and in our study the analysis is applied only to variables that have previously been identified as relevant to language diversity and endangerment (see pages Supplementary Information for justification of each variable selected). We are not clear on exactly which aspects of the models Prof Mufwene identifies as wrong for language endangerment, but we would be happy to respond to more specific criticisms of aspects of the model, methods or variables.

It is true that in modeling on can use any number of factors and get correct results, 1.even if the factors are not empirically valid. The question is how useful is the model in such cases when it does not enable the investigators to figure out what is actually going on or suggest to them what are the right questions to ask? If the predictions that the authors make are intended to help speakers interacting with each other in specific multilingual ecologies, within specific political and socioeconomic structures, avoid language loss, why should anyone accept the predictions as accurate if the model is based on factors that are remotely connected to local human behaviors? This is not a game of scoring success in predictions, which I actually think are not convincing if they have not been tested on specific cases, to which they are expected to apply?

2.3 The analysis is designed to detect factors that have global and regional association with language endangerment patterns. If there were no factors that are consistently identified as associated with language endangerment, then the model would find no significant predictors. We report only those predictors that have consistent association with language endangerment for which the association cannot be explained by chance alone or by these predictors' correlations with other variables included in the model. We think that this kind of modelling does indeed suggest some interesting questions to ask. For example, our analysis suggests that average number of years of schooling is associated with increased risk of language loss. We acknowledge on pages 8-9 and 13-14 that our analysis cannot untangle the possible causes of this pattern, because we are unable to capture the local and regional processes. Yet our result is consistent with smaller-scale studies on particular communities where individual behaviours, local conditions and regional policies are captured (see page 9-10 for references). So while our broadscale analysis cannot provide that level of detail, it is consistent with smaller scale studies and does suggest valuable areas for investigation at finer detail.

We are dealing with predictions intended to influence speakers'/agents' responsive behaviors. What I have read sounds like health care providers making predictions that ignore how people live their lives on a daily basis and focusing on climate change and behavior on islands. Or perhaps is this reminiscent of Milton Friedman recommending an economic development program to Chile without factoring the local factors in it and leading to a disaster?

2.4 As for point 2.3 above, it is not our intention that a global analysis should be used to inform local decision making, but it provides useful indicators of potentially important factors that impact on many (though not necessarily all) languages. Identifying years of schooling as a global correlate of language endangerment may prompt investigation of the influence of schools on language vitality at local level – in some cases, closer investigation may reveal current educational practices that are harmful to Indigenous language vitality, but in other cases it may be reveal particular local practices or forms of education are helping to maintain local language vitality. Global correlates don't provide local solutions, but they may help to focus attention on potential risk factors.

The inclusion of islands in the "analysis" is actually interesting, because it brings up the possibility--not usually discussed in the relevant literature--that a monolingual population isolated from other populations speaking different languages can lose their language. How? This needs explaining. For instance, I can imagine a natural disaster that causes them to die. However, if the population becomes connected to others by roads (with bridges) or waterways, under what conditions would the population lose their language? For instance, in the USA, the Ocracoke Island, inhabited by a White population, has experienced the endangerment of its brogue. On the other hand, the Gullah-speaking populations on islands on the same coast of South and North Carolina have not experienced the endangerment of Gullah in the same way. In the former case, the typically affluent White in-migrants from the mainland have mixed with the local population. In the latter cases, the White in-migrants have introduced or maintained residential segregation and have not mixed with the local Black islanders, the majority. If Gullah has been endangered at all, it is because of emigration, with the islanders leaving in search of better economic opportunities and finding themselves under pressure to speak differently in order to be competitive in the job market outside their islands. So, connection with the outside world is not necessarily dangerouns. POPULATION STRUCTURE is critical. It helps show that all "contacts" are not the same and do not produce the same effects.

2.5 We agree, and this is one key way in which language endangerment differs from species endangerment. We emphasize on page 6 that language endangerment need not involve the extirpation of a population: "It is important to emphasize that not all small languages are endangered, and that language loss does not necessarily result from a reduction in number of people in a particular culture or population, but often occurs when people shift from using their heritage language to a different language. Therefore the multilingual setting in which each language is embedded (referred to as the language ecology) plays a key role in endangerment, by influencing whether speakers shift to another language or adopt additional languages in their multilingual repertoires". We also emphasize this point in the predictive model, for example on page 25: "We emphasize that this procedure is specifically modelling the shift in number of first language (L1) speakers of a language, not the population they belong to. A population may thrive and its ethnic identity remain strong even while speakers are shifting to a different language". In the Supplementary Information we give specific examples of shift in language without reduction in the associated population from Europe, North America and Australia (page S36-37).

We included island in the analysis because it has previously been identified as a predictor of language endangerment, though our analysis does not offer support for island status being a significant predictor of endangerment status when other factors are taken into consideration.

After reading the paper, I cannot tell whether CONTACT is a "global" factor or not, why POPULATION MOVEMENT is apparently less important than LANGUAGE TRANSMISSION when in fact LANGUAGE TRANSMISSION itself is the explanandum (pace the literature on the subject matter) and not the explanans for language loss. The literature is generally descriptive and not explanatory. It's not enough to say that discontinued or decreased language transmission or language shift leads to language loss. One must explain why these phenomena are happening. Reproducing factors that are often conflicting and not reliable, as the authors also recognize, doesn't help the model do the good job that the authors want and claim it to do. This modeling becomes an ill-conceived one in my view, although the technology is impressive.

2.6 Indeed in our analysis language transmission is the explanandum. Our dependent (response) variable is endangerment level, based on the EGIDS score which is largely, though not wholly, reflects patterns of language transmission (see page 5, and Table 1). We agree that population movement is a critical factor in language endangerment patterns in space and time. While we are not able to test this directly, due to lack of appropriate global data, we offer a possible interpretation of the significant association between road density and language endangerment (pages 7-8). We have added a statement to page 5 acknowledging that we cannot include data on population movement, although it is undoubtedly a major factor in patterns of language diversity and endangerment: ". For example, patterns of human migration and expansion and contraction of speaker populations will not be captured fully in contemporary language distribution data."

We define our localised measure of language contact on page 6-7. We have checked the use of the word "contact" in other parts of the ms to remove any ambiguity. For example, we have modified the statement on age 6 to read "Our results show that direct language contact with neighbouring languages, as reflected in the number languages with overlapping or touching distributions, is not in itself a threatening process."

We acknowledge throughout the ms that our set of factors are limited to those which we can obtain appropriate data on, and this is unavoidably is a limited set of possible influences on language. We do not claim that his approach can provide the whole picture of global language endangerment, but we do feel it can add some interesting aspects our understanding of language loss.

I will save myself and the editors time and subordinate my reactions to those of other reviewers if their reactions are positive. I can respond to the published article if invited to, in order to generate more discussion in which other readers may engage. But my opinion on the usefulness of this article and its supplement is clear: A lot of reservations.
Salikoko Mufwene

2.7 We appreciate that Prof Mufwene has reservations about a quantitative, large-scale approach to language endangerment and loss, and we again emphasize that we think a plurality of approaches is valuable for the field. We do not suggest this approach is superior but we do think that it is useful. Global, quantitative analyses are valuable for identifying broadscale patterns and for emphasizing the potentially catastrophic loss of language diversity, adding to a diverse toolkit of approaches to understanding language endangerment and loss. We certainly welcome an ongoing debate about the value of this approach, and we hope that publication of this global analysis will contribute to that discussion.

Reviewer #3 (Remarks to the Author):

Most of the comments to reviewers have been addressed in this version. There are, however, a few points which still need some further discussion and clarification.

pp5-6: Just a comment on the reviewer's remark about urban contact ecologies. Another place where stable multilingualism obtains is where there is both language as a strong marker of ethnic identity and no single dominant group. In such cases, multiple languages seems to be pretty stable. There's anecdotal discussion of this in South Africa, townships, but it's also seen historically in Arnhem Land (Australia). I make this point to agree with the authors in point 2.5

I was surprised by Prof. Mufwene's claim that there is no language loss in Africa, apart from Khoi San. I'm not an Africanist but endangeredlanguages.com (data curated by regional experts) shows plenty of language endangerment on the continent.

I'm not satisfied with the comments on granularity of data. While I'm sympathetic to the authors' predicament -- that some key indicators are only available at the country level -- I'm not satisfied that there's nothing more to say than "this is the best we could do". I would like to see more validation checks to show that the country-level data is not distorting the findings. It's not sufficient to use data which is clearly problematic without some attempt to investigate whether the problematic aspects of the dataset are distorting the results.

3.1 We understand the reviewer's concerns, and we have added a statement to page 9 acknowledging that granularity of data may influence outcomes of the analysis.

However, we can show that for this study, it does not create the kind of distortion they are concerned about. This issue only applies to 4 out of 51 variables in our dataset, and we can detect no evidence of influence of granularity on the results in these four variables, as we discuss in detail here.

Of the 51 variables included in this study, 31 are directly associated with specific languages and do not use country level information, instead they are based on information available for each language (e.g. L1 speaker population size), estimated from gridded data either within the distribution of the language (e.g. mean growing season), or for the immediate neighbourhood of the language location (e.g. proportion of threatened languages in the area).

There are ten variables that reflect official national languages: obviously these have to be country-level, as it is inherent in the variable itself. Similarly, the variables "Policy affirming minority language education" and "Education spending as % of GDP" are national-level variables because these variables specifically refer to policies that are enacted at national level (see discussion on page 9), which might influence language vitality within the country (though we do not find evidence that they do). Clearly, we cannot measure national policy at a finer scale than country-level.

One of the national level variables, "Increase in urbanisation", is included because it provides the potential to reflect population movement, which cannot be captured on a within-language scale (for

example, we want to capture rural populations moving to capital cities). However, this variable is also reflected in a finer-scaled variable "Change in built environment", so in this sense this variable is tested at two levels of granularity.

There are only four variables that are measured at country level that could conceivably be measured at a larger or smaller scale, if appropriate data were available: Average years of schooling, Gross Domestic Product per capita, GINI, Life Expectancy at age 60. For all four of these country-level variables, each language is given the weighted average value of the countries that it overlaps – therefore two nearby languages could have different values if they run over national borders. Of these four variables, the only one with a significant association with language endangerment is 'Average years of schooling'. The problem of granularity of data for this variable is discussed extensively on pages 8-9. However, we note that smaller scale studies support this conclusion (see pages 8 and 12-13 for references and both local and within-country scale), which suggests that granularity is not a major problem here because the same pattern is reflected at global, regional and local scales. We have added this statement to page 9: "The spatial scale of the variables reflecting education policy and outcomes may fail to capture variation within countries. Reliable statistics on average years of schooling are, for most parts of the world, only available as national averages, even though years of schooling may vary within a country, particularly between socioeconomic groups, or when comparing rural and urban populations. However, we note that the same effects have been reported in local scale studies: for example, in a remote northern Australian Indigenous community, number of years schooling is associated with Indigenous language use across all generations, from elders to children. Collection of data on spatial variation in number of years of schooling across larger areas would allow the generality of this relationship to be tested at a range of spatial scales."

For the remaining three variables, sub-national data is available only for a handful of large countries (e.g. USA) or for smaller countries that have relatively low diversity of autochthonous languages (e.g. United Kingdom). For example, GDP is available for subnational regions of USA, Japan, China, India, Australia, and Gini for states of Australia and USA. While life expectancy is available for states in these countries plus India, the measure we use in this study LE60 is not generally available at subnational level. Because of this geographically and culturally skewed availability of subnational data on socioeconomic indicators, we cannot use it to test the influence of data granularity on global language endangerment, because it would represent a very limited and biased proportion of the world's languages (in other words, the test itself could be a greater distortion of the data due to a geographically and culturally biased sample).

We note also that these three variables are not significant in any region: if the lack of significance was due to granularity we would expect to see stronger associations in regions where countries have smaller geographic extent and less within-country variation in socioeconomic variables, for example in Oceania. Additionally, some of our variables are intended to address variation in the socioeconomic status of languages within each country, even though they are collected at a national level. These include the Gini index, which describes economic inequality within each country, as well as the world language and minority language education policy variables, which describe aspects of social inequality of languages within countries. We have added a discussion of these points to page S21.

In conclusion, we cannot see a clear way that country-level data could be distorting the findings, because it applies to a small proportion of the dataset. All of these are consistent with finer scale studies, or across regions where countries are smaller and more uniform in socioeconomic indicators. In the case of the single significant national-level variable (average years of schooling), we think that it points to an important effect that needs to be investigated with finer scale data, and we highlight some intra-country and local-scale analyses that seem to confirm the result at finer levels of investigation (pages 8-9). For the remaining country-level variables that are not significantly associated with language endangerment – GDP, GINI, LE60 - we cannot rule out that a finer resolution would reveal significant associations that are not evident from national level statistics, a point acknowledged on page 9 and discussed in more detail on page S21. But we do not believe that our use of country level statistics for these four variables could lead to misleading results. The variables that do allow comparison across levels of resolution (e.g. our two variables on urbanisation at different scales, or between regions that vary in the degree of resolution of GPD and LE60 data) do not show any difference in results between variables or regions depending on resolution.

Likewise, I think the authors misunderstood my point about the influence of history (their 3.4). My point is about uniformitarian assumptions. They use *current* data to infer endangerment associations, which is not valid for languages whose causes of endangerment directly associated with conditions which no longer apply. This can't

be dealt with simply as a caveat, and by projecting language loss into the future. That just expands the uniformitarian problems into the future as well as into the past. It's also not addressed by claiming the model is a global one, since these point affect a substantial percentage of the languages in the sample (since so many languages in North America and Australia are endangered, those two countries alone must account for around a quarter of the data points).

3.2 Thank you for this clarification. We acknowledge this point on page 5: "Furthermore, language endangerment and loss is an ongoing process, and there may be historical factors that caused dramatic reduction in L1 speakers that will not be captured in current values of socioeconomic variables, such as massacres of Indigenous populations or ethnic groups, punishing people for speaking their language and separating children from parents."

We have made an additional statement to emphasize this point, and added a further reference to this issue: "Patterns of language endangerment may at least partially reflect past influences, such that current predictors might not fully capture important processes that resulted in the current endangerment status (a phenomenon known in conservation biology as an extinction filter effect)."

Our statistical approach specifically addresses regional differences in patterns of endangerment. We include separate independent variables for global effect and regional effects of each predictor and find the best model after accounting for the trade-off between the explanatory power and the complexity of the model. So if the endangerment status of languages in North America and Australia are affected by different processes to other regions, then a model that allows two regional effects reflecting languages in North America and Australia and one "global" effect representing all other languages in all other regions will have the same explanatory power but fewer parameters than a model with global effect reflecting languages in North America and Australia and many regional effects for the other regions. Therefore, our statistical approach reduces the influence of any region with more languages than the other regions on biasing the global effect.

Predictions are based on the full model which includes region-specific factors, so that, for example, the particular pattern of influences in North America is used to project future values of endangerment of North American languages, while a different set of influences will be used to project future values of South American languages.

3.8 The rationale for excluding sign languages isn't satisfactory. You could search for "spoken L1 language" + "sign languages", for example (I agree that the exclusion of other languages is justified). Second, I'm not at all convinced about the need for controlling for relatedness when geography is also included in the model, unless relatedness is some proxy for another factor like morphological complexity. (See more on this below.) Is there any indication that population figures for many spoken languages are a lot more reliable than the sign language data? (In my experience such data are not very accurate for spoken languages either). Many spoken language communities for endangered languages are also embedded in the distribution of other spoken languages. Sign languages, like spoken languages, are affected by pressures around education (wasn't schooling one of the significant factors in the model?). Whether parents use the language or not is presumably also significant for spoken languages. That is, I'm not convinced that the reasons given for excluding sign languages are sufficient.

3.3 We wish to stress that we think that endangerment patterns of sign languages is very important, and we would love to see a separate analysis of this, but that it is not possible to include signed languages in this analysis for several reasons. We have added a new section to the Supplementary Information (Section 2.1.6, pages S15 to S18) to give this important point the detail it deserves. This new section provides an extensive discussion of the barriers to inclusion of signed languages in the same analysis as L1 spoken languages at this point in time.
To summarize:
(1) there is insufficient information on signed languages in current databases, with incomplete lists of global sign languages, and with half of the listed languages without any information on L1 population size or endangerment status;
(2) Distribution data is available for less than 5% of signed languages in the database, so that we cannot derive the majority of variables included in this study for signed languages;
(3) Signed languages are threatened by a largely non-overlapping set of threatening processes to spoken L1 languages (such as declining rates of infection-related hearing loss), so cannot meaningfully be combined in the same analysis as spoken L1 languages;
(4) There is essentially no phylogenetic information for signed languages as they are all grouped into "pseudo-families" that represent modality not relationships. For spoken languages, relatedness accounts for 35% of the autocorrelation between languages, so by using an unresolved "star phylogeny" for signed languages we would either be failing to capture this source of non-independence, or, if signed

languages do not show any similarity to related signed or spoken languages, then it would be inappropriate to use the same model for both signed and spoken languages.

We hope that this study will prompt other researchers to develop databases for signed languages that would allow a similar study of patterns of endangerment as we have applied to spoken languages, but the current state of available data does not permit their inclusion in this global analysis, nor is there is sufficient data to allow a separate analysis of endangerment in signed languages. We hope this situation will change in future.

3.24 Why should related languages share patterns of endangerment that are separate from associations of geography, social position in the group, or other points that also have nothing to do with genetic relatedness?

3.4 Our model is designed to evaluate whether related languages do share patterns of endangerment, and to allow for these patterns if they do. If relatedness does not predict patterns of endangerment, then the contribution of phylogeny to the results will be very low or effectively zero (see page 19, page 22). So we are able to ask, without prior assumptions, whether there is a signal of phylogeny in endangerment patterns. In this case, 65% autocorrelation among languages' endangerment levels is due to spatial proximity and 35% autocorrelation is due to phylogenetic relatedness. Therefore we show that while spatial distribution is a stronger predictor of endangerment status than phylogeny, language endangerment is at least partially associated with relationships between languages (ie related languages are more likely to be more similar in endangerment status than they would to a randomly chosen language).

It is important to note that identifying a phylogenetic signal in endangerment status is not the same as saying that endangerment status is heritable or directly linked to genetic relationships (see statement on page 19). It means that related languages are more likely to share endangerment status, even if we don't know the cause of that shared endangerment. For example, in biology, showing that there is phylogenetic signal in threat status does not suggest species inherit extinction risk, but that species can share traits with their relatives that make them more vulnerable to extinction. For example, in mammals, threat caused by harvesting shows phylogenetic signal (e.g Fritz S & Purvis A 2010 Selectivity in mammalian extinction risk and threat types: a new measure of phylogenetic signal strength in binary traits. Conservation Biology 24:1042). This does not mean that species inherit being harvested from their ancestors, but that related species are likely to be more similar in their response to being harvested than randomly chosen species will be.

We now make this more clearly point on pages 19 - 20: "However, if we do detect a significant contribution of phylogeny, this tells us that languages likely to be more similar in their endangerment status to related languages than they are to randomly selected languages. This observation does not imply that species inherit either their endangerment status or threatening processes from their ancestors, but that relatives show patterns of similarity of endangerment. If this is the case then we need to account for this phylogenetic non-independence in our analysis, so that we can identify factors that are significantly associated with endangerment above and beyond the patterns caused by language groups having more similar patterns of endangerment than expected by chance.

Reviewer #4 (Remarks to the Author):

Overall, I'm happy with the edits to this paper. The prediction section seems much more reasonable now and I'm generally happy with the responses to my other comments. This is a thorough and important paper and I think the authors have done a great job.

I just have a few final, minor, things:

I think in the abstract it might be better to say something like "We predict that, based on demographics and without intervention, language loss is expected to triple…" rather than the current sentence at lines 14-15. Otherwise it's implied that the predictive model uses the variables from the explanatory model.

4.1 Actually the predictive model and the explanatory model are the same thing – we use the full model to predict future status, but we only change the value of variables for which we have reasonable future projections, and we give all other variables their current value. We have modified this sentence to read "We use our model to forecast future patterns of language endangerment, and show that, without

intervention, language loss is expected to triple within forty years, with more than one language lost per month for the rest of this century."

At Line 80, specify that the endangerment scale is EGIDS and spell it out. I know that you mention this in the methods, but most people reading the paper won't start with the methods, and given you use EGIDS in figures it's good to explain what it is without someone having to refer to methods.

4.2 We have changed this line to "We use an endangerment scale based on the Expanded Graded Intergenerational Disruption Scale (EGIDS), which incorporates on a range of factors including domains of use and intergenerational transmission"

At L125, you list "threatened languages" as one of the five predictors, again to stop the reader having to refer to elsewhere to understand this sentence, better to say "the number of threatened languages in the vicinity". Linking with this, you then describe this variable as "languages in the region are also endangered" at Line 154 . This inconsistency between threatened and endangered can be confusing, better to change to threatened here to be consistent.

4.3 Changed to "the number of endangered languages in the immediate neighbourhood"

Somewhere in L161-164 it could be good to say "as discussed earlier" just to help any reader that may be confused and think "hasn't this already been mentioned" (clearly I'm down to very minor comments now!)

4.4 Sorry but we are not entirely clear what the required edit is here – this is, in fact, the first discussion of this point in the ms so it's not obvious where we should refer back to. Happy to incorporate this suggestion if a more detailed direction is given.

L200, again to help the reader it would be good to start this paragraph with something like "We didn't find a correlation between education policy and language threat status, this may be because our data on education policy is necessarily coarse grained…"

4.5 We have added the following statement: "Similarly, our data on education policy is necessarily coarse grained, which may mask some patterns at local scales"

L250-254. The reader may be left confused as to why you didn't include land-use and climate predictors in the main paper, I wonder whether at the start of the prediction section (e.g. somewhere in the L226-229 paragraph) you could explain that you were not able to obtain adequate data for most predictors and so instead decided to focus on L1 speakers, and quality at L250 that though you can make predictions with climate and land-use, they were not major contributors to the global model and so are more relevant at a regional scale.

4.6 We removed these predictions from the main paper at the request of Referee 4 in the previous round of review, not because there was no available data. Similarly, it is not a problem that climate and land-use variables are significant at regional rather than global level – our analysis uses a spatially explicit model and models endangerment using the predictors identified in each region, so change in climate or land use will only influence future predictions for languages in areas where those factors have been identified as significant predictors of endangerment.

To avoid any confusion on this point, we have now expanded this paragraph on page 11-12 as follows: "In addition to demographic shift, our model also identifies predictors of language endangerment that are likely to change over time. For some of the variables associated with language endangerment, such as average years of schooling, we lack an adequate predictive model that is global in extent but would allow for regional variation. But there are some variables identified as significant predictors of language endangerment at regional levels, such as land-use and climate, that we can predict future values based on current trends (see Supplementary Information 5.2). For example, we can use climate change models to predict future values of climate variables at all points of the globe, and we can use information on rates of change in land use in each grid cell to project possible future values in that grid cell. Clearly, such predictions should be regarded as possible values only, and all such future projections are subject to caveats: for example, we chose a mid-range climate model so the future values could show a sharper rate of increase if global carbon emissions are not reduced, and the land-use projections are based on the future rate of change mirroring the average rate of change in the last few decades, when local factors may cause those rates of change to either increase or decrease. But it is a useful exercise to add climate and landuse to the predictive model to illustrate the potential for forward prediction of variables impacting endangerment status. The results of the predictions based on generational shift and

demographic transition are shown in Figure 2 & 3. Predictions which are additionally adjusted for change in environmental and land-use variables show qualitatively the same results (Figure S8-S11)."

Figures.

Re Figure 1, I have attached a figure demonstrating what I was suggesting by grouping factors, where a square bracket is added to demonstrate which wider group each factor belongs to. It's just to help the reader be able to interpret the figure without needing to refer to Table 1 excessively. If possible, I would recommend reordering the variables so that they are in the same order as given in Table 1. Also, L1 Prop should be grouped with the other Diversity factors, no?

4.7 Unfortunately, we did not receive this attachment with the review, so we have to interpret this using the written comment alone. We have now modified this figure to have boxes around the variables according to the categories in Table 1 and recoloured some variables in the bar plots, so that the variables in the same category have more similar colours.

Re Figure 3: By excessive use of colour, I mean there is extra colour in this figure that does not convey extra information, which can be confusing. I would recommend removing all colour from A) (or, alternatively, remove the side bars saying "endangered" and "sleeping" and instead have a legend for blue and red.) and in B), I would remove the red colour on "No L1 Speakers (9-10)" and make this the same grey as "Living L1 speakers (6b-8b)" (red already indicates "little" so is confusing to have it twice). It's also confusing here that in A) the two panels use the same y axis (ranging from 0.1-0.4), while in B) the axis is repeated. Maybe in A) remove the small bit of white space between the upper and lower panels? Finally, I'm not convinced of the utility of the dashed lines in an already busy figure, perhaps it would be better to specify in the caption that the "current" estimates are modelled, but roughly align with actual values?

4.8 We have made changes to Figure 3 to improve it, following these comments. We have removed the side bars and central white line from Panel A, and split Panel B into two separate panels (B and C). We have changed the colour of the side bar on B to avoid using red for two different categories of information. Colour is now only used to distinguish different information. In A), the red violins are a different set of languages than the blue violins. The dashed lines provide important information (current levels) that would otherwise not be available for comparison, so we think they should stay.

We are happy to take editorial direction on these suggested adjustments to the figure and if the editor feels it would be better to have more panels or monochrome figures we can make the adjustment.

********************END********************

natureresearch

Decision Letter, second revision:

21st September 2021

Dear Lindell,

Thank you for submitting your revised manuscript "Global predictors of language endangerment and the future of linguistic diversity" (NATECOLEVOL-210513613B). It has now been seen again by reviewer 3, whose comments are below. The reviewer find that the paper has improved in revision, and therefore we'll be happy in principle to publish it in Nature Ecology & Evolution, pending minor revisions to comply with our editorial and formatting guidelines.

If the current version of your manuscript is in a PDF format, please email us a copy of the file in an editable format (Microsoft Word or LaTex)-- we can not proceed with PDFs at this stage.

We are now performing detailed checks on your paper and will send you a checklist detailing our editorial and formatting requirements in about a week. Please do not upload the final materials and make any revisions until you receive this additional information from us.

Thank you again for your interest in Nature Ecology & Evolution. Please do not hesitate to contact me if you have any questions.

[REDACTED]

Reviewer 3 comments:

I've now reread the revised submission and I think it's good to go. The authors have now made a serious effort to both qualify their conclusions, where warranted, and to discuss some of the complexifying factors in more detail.

Our ref: NATECOLEVOL-210513613B

24th September 2021

Dear Dr. Bromham,

Thank you for your patience as we’ve prepared the guidelines for final submission of your Nature Ecology & Evolution manuscript, "Global predictors of language endangerment and the future of linguistic diversity" (NATECOLEVOL-210513613B). Please carefully follow the step-by-step instructions

provided in the attached file, and add a response in each row of the table to indicate the changes that you have made. Please also check and comment on any additional marked-up edits we have proposed within the text. Ensuring that each point is addressed will help to ensure that your revised manuscript can be swiftly handed over to our production team.

We would like to start working on your revised paper, with all of the requested files and forms, as soon as possible (preferably within two weeks). Please get in contact with us immediately if you anticipate it taking more than two weeks to submit these revised files.

When you upload your final materials, please include a point-by-point response to any remaining reviewer comments.

If you have not done so already, please alert us to any related manuscripts from your group that are under consideration or in press at other journals, or are being written up for submission to other journals (see: https://www.nature.com/nature-research/editorial-policies/plagiarism#policy-on-duplicate-publication for details).

In recognition of the time and expertise our reviewers provide to Nature Ecology & Evolution's editorial process, we would like to formally acknowledge their contribution to the external peer review of your manuscript entitled "Global predictors of language endangerment and the future of linguistic diversity". For those reviewers who give their assent, we will be publishing their names alongside the published article.

Nature Ecology & Evolution offers a Transparent Peer Review option for new original research manuscripts submitted after December 1st, 2019. As part of this initiative, we encourage our authors to support increased transparency into the peer review process by agreeing to have the reviewer comments, author rebuttal letters, and editorial decision letters published as a Supplementary item. When you submit your final files please clearly state in your cover letter whether or not you would like to participate in this initiative. Please note that failure to state your preference will result in delays in accepting your manuscript for publication.

Cover suggestions

As you prepare your final files we encourage you to consider whether you have any images or illustrations that may be appropriate for use on the cover of Nature Ecology & Evolution.

Covers should be both aesthetically appealing and scientifically relevant, and should be supplied at the best quality available. Due to the prominence of these images, we do not generally select images featuring faces, children, text, graphs, schematic drawings, or collages on our covers.

We accept TIFF, JPEG, PNG or PSD file formats (a layered PSD file would be ideal), and the image should be at least 300ppi resolution (preferably 600-1200 ppi), in CMYK colour mode.

If your image is selected, we may also use it on the journal website as a banner image, and may need to make artistic alterations to fit our journal style.

Please submit your suggestions, clearly labeled, along with your final files. We'll be in touch if more information is needed.

natureresearch

Nature Ecology & Evolution has now transitioned to a unified Rights Collection system which will allow our Author Services team to quickly and easily collect the rights and permissions required to publish your work. Approximately 10 days after your paper is formally accepted, you will receive an email in providing you with a link to complete the grant of rights. If your paper is eligible for Open Access, our Author Services team will also be in touch regarding any additional information that may be required to arrange payment for your article.

Please note that <i>Nature Ecology & Evolution</i> is a Transformative Journal (TJ). Authors may publish their research with us through the traditional subscription access route or make their paper immediately open access through payment of an article-processing charge (APC). Authors will not be required to make a final decision about access to their article until it has been accepted. Find out more about Transformative Journals

<B>Authors may need to take specific actions to achieve compliance with funder and institutional open access mandates. For submissions from January 2021, if your research is supported by a funder that requires immediate open access (e.g. according to Plan S principles) then you should select the gold OA route, and we will direct you to the compliant route where possible. For authors selecting the subscription publication route our standard licensing terms will need to be accepted, including our self-archiving policies. Those standard licensing terms will supersede any other terms that the author or any third party may assert apply to any version of the manuscript.

Please note that you will not receive your proofs until the publishing agreement has been received through our system.

For information regarding our different publishing models please see our Transformative Journals page. If you have any questions about costs, Open Access requirements, or our legal forms, please contact ASJournals@springernature.com.

Please use the following link for uploading these materials:
[REDACTED]

If you have any further questions, please feel free to contact me.

[REDACTED]

Final Decision Letter:

natureresearch

27th October 2021

Dear Professor Bromham,

I am writing in the temporary absence of my colleague Dr. Luiseach Nic Eoin.

We are pleased to inform you that your Article entitled "Global predictors of language endangerment and the future of linguistic diversity", has now been accepted for publication in Nature Ecology & Evolution.

Before your manuscript is typeset, we will edit the text to ensure it conforms to house style.

Once your manuscript is typeset you will receive a link to your electronic proof via email, with a request to make any corrections as soon as possible. If you have queries at any point during the production process then please contact the production team at rjsproduction@springernature.com. Once your paper has been scheduled for online publication, the Nature press office will be in touch to confirm the details.

Acceptance of your manuscript is conditional on all authors' agreement with our publication policies (see www.nature.com/authors/policies/index.html). In particular your manuscript must not be published elsewhere and there must be no announcement of the work to any media outlet until the publication date (the day on which it is uploaded onto our web site).

Please note that <i>Nature Ecology & Evolution</i> is a Transformative Journal (TJ). Authors may publish their research with us through the traditional subscription access route or make their paper immediately open access through payment of an article-processing charge (APC). Authors will not be required to make a final decision about access to their article until it has been accepted. Find out more about Transformative Journals

<B>Authors may need to take specific actions to achieve compliance with funder and institutional open access mandates. For submissions from January 2021, if your research is supported by a funder that requires immediate open access (e.g. according to Plan S principles) then you should select the gold OA route, and we will direct you to the compliant route where possible. For authors selecting the subscription publication route our standard licensing terms will need to be accepted, including our self-archiving policies. Those standard licensing terms will supersede any other terms that the author or any third party may assert apply to any version of the manuscript.

In approximately 10 business days you will receive an email with a link to choose the appropriate publishing options for your paper and our Author Services team will be in touch regarding any additional information that may be required.

You will not receive your proofs until the publishing agreement has been received through our system.

natureresearch

If you have any questions about our publishing options, costs, Open Access requirements, or our legal forms, please contact ASJournals@springernature.com

An online order form for reprints of your paper is available at https://www.nature.com/reprints/author-reprints.html. All co-authors, authors' institutions and authors' funding agencies can order reprints using the form appropriate to their geographical region.

We welcome the submission of potential cover material (including a short caption of around 40 words) related to your manuscript; suggestions should be sent to Nature Ecology & Evolution as electronic files (the image should be 300 dpi at 210 x 297 mm in either TIFF or JPEG format). Please note that such pictures should be selected more for their aesthetic appeal than for their scientific content, and that colour images work better than black and white or grayscale images. Please do not try to design a cover with the Nature Ecology & Evolution logo etc., and please do not submit composites of images related to your work. I am sure you will understand that we cannot make any promise as to whether any of your suggestions might be selected for the cover of the journal.

You can now use a single sign-on for all your accounts, view the status of all your manuscript submissions and reviews, access usage statistics for your published articles and download a record of your refereeing activity for the Nature journals.

To assist our authors in disseminating their research to the broader community, our SharedIt initiative provides you with a unique shareable link that will allow anyone (with or without a subscription) to read the published article. Recipients of the link with a subscription will also be able to download and print the PDF.

You can generate the link yourself when you receive your article DOI by entering it here: <a href="http://authors.springernature.com/share">http://authors.springernature.com/share<a>.

[REDACTED]

P.S. Click on the following link if you would like to recommend Nature Ecology & Evolution to your librarian http://www.nature.com/subscriptions/recommend.html#forms

** Visit the Springer Nature Editorial and Publishing website at www.springernature.com/editorial-and-publishing-jobs for more information about our career opportunities. If you have any questions please click here.**
